# Curcumin inhibits chondrocyte apoptosis and inflammation in osteoarthritis via the miR-338-3p/EIF4A1 signaling axis

**DOI:** 10.1186/s41065-026-00674-x

**Published:** 2026-04-09

**Authors:** Na Dai, Bin Shi

**Affiliations:** Department of TCM Orthopedics and Traumatology, Waist and Leg Pain, Shandong First Medical University Affiliated Hospital for Neck, Shoulder, Waist and Leg Pain , No.18877 Jingshi Road, Lixia District, Jinan City, Shandong Province 250000 China

**Keywords:** Osteoarthritis (OA), Curcumin, miR-338-3p, EIF4A1, IL-6, MMP-13, ADAMTS

## Abstract

**Objective:**

Osteoarthritis (OA) is a degenerative joint disorder mainly characterized by articular cartilage degradation. Curcumin, the primary bioactive compound derived from turmeric, exhibits anti-inflammatory, antioxidant, and anti-catabolic activities. This study aimed to explore the therapeutic potential of curcumin in OA and its underlying mechanism involving the microRNA-338-3p (miR-338-3p)/Eukaryotic Translation Initiation Factor 4A1 (EIF4A1) signaling pathway.

**Methods:**

Mouse models of OA were established, including groups treated with curcumin or intra-articular injection of miR-338-3p agomir, along with an in vitro chondrocyte model. Modulation of miR-338-3p and EIF4A1 expression was achieved through siRNA interference. Gene expression was quantified via quantitative real-time polymerase chain reaction and Western blot; the targeting interaction between miR-338-3p and EIF4A1 was confirmed using a luciferase reporter assay; and cellular viability and inflammatory mediator levels were evaluated with Cell Counting Kit-8, flow cytometry, and enzyme-linked immunosorbent assay. Cartilage tissue pathology was also examined.

**Results:**

EIF4A1 was markedly upregulated in OA cartilage, whereas miR-338-3p targeted and suppressed EIF4A1 expression. Curcumin administration elevated miR-338-3p levels and reduced EIF4A1 expression. Moreover, curcumin, miR-338-3p overexpression, and EIF4A1 knockdown attenuated chondrocyte apoptosis, enhanced proliferation, and modulated apoptosis-related protein expression (decreasing cleaved caspase-3 and Bax, increasing Bcl-2). Curcumin also suppressed the secretion of inflammatory cytokines (IL-6, TNF-α, IL-1β, etc.), decreased synthesis of matrix-degrading enzymes (Matrix Metalloproteinase-13 (MMP13), A Disintegrin and Metalloproteinase with Thrombospondin Motifs (ADAMTS)), and reduced OARSI scores, ultimately ameliorating OA progression.

**Conclusion:**

These results demonstrate that curcumin mitigates chondrocyte apoptosis, attenuates cartilage inflammation, and downregulates joint matrix-degrading enzyme expression in OA through the miR-338-3p/EIF4A1 axis, suggesting this pathway as a promising target for gene therapy in OA.

**Supplementary Information:**

The online version contains supplementary material available at 10.1186/s41065-026-00674-x.

## Introduction

Osteoarthritis (OA) represents a degenerative joint disorder associated with the highest global disability rate, defined by gradual degradation of the cartilage matrix, chondrocyte apoptosis, and synovial inflammation [1]. As reported in statistics from the World Health Organization, the prevalence of OA among individuals aged 60 years and above reaches 18% across the globe, and the condition is expected to become the fourth primary cause of disability [2]. Currently available first-line pharmacological interventions—including acetaminophen and Cyclooxygenase-2 (COX-2) inhibitors—only deliver short-term pain alleviation without preventing the progression of the disease. In contrast, joint replacement surgery is accompanied by potential risks such as infection and prosthetic loosening [3–5]. In recent years, biological therapeutic strategies targeting inflammatory factors (e.g., antibodies against interleukin-1 beta [IL-1β]) or matrix-degrading enzymes (e.g., inhibitors of Matrix Metalloproteinase-13 [MMP-13]) have demonstrated limited effectiveness and high costs in clinical trial settings [6]. Therefore, developing natural compounds with low cost, multi-target regulatory capacity, and high safety has become an important direction in OA treatment research.

Curcumin, the primary bioactive component extracted from turmeric (Curcuma longa), has been widely reported to exert protective effects in the context of OA [7, 8]. Research by Henrotin et al. [9–11]. demonstrated that curcumin suppresses nuclear translocation of Nuclear Factor kappa-light-chain-enhancer of activated B cells (NF-κB) by inhibiting phosphorylation of Inhibitor of κB alpha, thereby reducing the expression of inflammatory cytokines including Tumor Necrosis Factor-alpha (TNF-α) and interleukin-6 (IL-6). Beyond its anti-inflammatory properties, curcumin has also been shown to activate the Nuclear factor erythroid 2-related factor 2/antioxidant response element (Nrf2/ARE) signaling cascade. This activation helps decrease reactive oxygen species (ROS) production and mitigates oxidative damage in chondrocytes [8, 12, 13]. Additionally, curcumin modulates metabolic activity by regulating phosphorylation events in the Wnt/β-catenin pathway, influencing critical components such as β-catenin and Glycogen Synthase Kinase-3 beta (GSK-3β), which in turn stimulates synthesis of type II collagen and aggrecan [14]. It is noteworthy that current research has largely focused on protein-related mechanisms [14], while epigenetic regulation—especially processes mediated by miRNAs—remains comparatively underexplored. As a result, elucidating miRNA-involved pathways through which curcumin may ameliorate OA has become a topic of growing interest in recent scientific investigations.

Recent progress in epigenetic research, especially the characterization of microRNA-338-3p (miR-338-3p) and Eukaryotic Translation Initiation Factor 4A1 (EIF4A1), has provided new perspectives for OA therapy. Li et al. [15]. observed a downregulation of miR-338-3p in cartilage specimens from OA patients relative to normal tissues, using high-throughput sequencing. Subsequent integration of bioinformatic prediction and luciferase reporter assays verified that miR-338-3p specifically targets the 3’-untranslated region (3′UTR) of EIF4A1 mRNA [16]. EIF4A1, which functions as a translation initiation factor, exhibits elevated expression in osteoarthritic cartilage and facilitates Cysteine-dependent aspartate-specific protease 3 (Caspase-3) activation via suppression of AKT phosphorylation—particularly at the Ser473 residue [17]. In vivo studies further demonstrated that administration of miR-338-3p mimics via intra-articular injection attenuates cartilage erosion and ameliorates OA manifestations in murine models [18, 19]. These results underscore the significance of both miR-338-3p and EIF4A1 as potential therapeutic targets for mitigating cartilage degradation in OA. Nevertheless, the relationship between the miR-338-3p/EIF4A1 pathway and the ameliorative actions of curcumin in OA remains inadequately explored.​.

Based on the identified research void and recent advancements in the field, this study introduces a novel hypothesis focusing on a regulatory mechanism involving “curcumin–miR-338-3p/EIF4A1”. Alterations in miR-338-3p expression and EIF4A1 protein abundance upon administration of different doses of curcumin were evaluated via quantitative real-time polymerase chain reaction (qRT-PCR) and Western blot. To examine chondrocyte proliferation and possible combinatory effects with curcumin, EIF4A1 was knocked down using siRNA, in conjunction with miR-338-3p overexpression. Moreover, an OA animal model generated by destabilization of the medial meniscus (DMM) was employed to validate the role of curcumin and the miR-338-3p/EIF4A1 axis in OA development. Subsequent analyses aimed to clarify the underlying molecular mechanisms.

## Materials and methods

### OA animal models

A cohort of 40 male C57BL/6J mice aged seven weeks and weighing between 20 and 25 g was supplied by Guangdong Vital River Laboratory Animal Technology Co., Ltd. Only male mice were used in this study to eliminate the confounding protective effects of estrogen on osteoarthritis progression; however, we acknowledge that this constitutes a limitation regarding sex differences, and future studies will include female cohorts to address this gap. All animals were maintained under specific pathogen-free conditions using individually ventilated caging systems, with a standardized 12-h light/dark cycle. Ambient temperature was controlled at 22–24 °C, and relative humidity was kept at approximately 60%. Food and water were available without restriction. The mice were randomly assigned to one of four groups (*n* = 10 per group): Sham-operated (Sham), OA model, OA + Curcumin, and OA + Curcumin + miR-338-3p antagomir. OA was induced via surgical destabilization of the medial meniscus (DMM). Specifically, anesthesia was administered by intraperitoneal injection of sodium pentobarbital at a dosage of 40 mg/kg. A medial parapatellar incision was made to expose the knee joint, followed by dislocation of the patella and sectioning of the medial meniscotibial ligament. Mice in the Sham group underwent an identical surgical procedure but without transection of the ligament [20]. Immediately after DMM surgery, mice in the OA + Curcumin group and OA + Curcumin + miR-338-3p antagomir group received daily oral gavage of curcumin (purchased from Sigma, dissolved in corn oil) at a dose of 50 mg/kg, which lasted for 8 weeks. Four weeks after the start of curcumin treatment, these two groups further received weekly intra-articular injections: one group was injected with 100 µL antagomir-negative control (antagomir-NC), and the other with 100 µL miR-338-3p antagomir (supplied by Ribobio, China) [21]; this injection regimen continued for 4 consecutive weeks [22, 23]. Sham and OA model groups were given equivalent volumes of corn oil via oral gavage daily for 8 weeks. All mice were euthanized 8 weeks post-surgery. Synovial fluid and articular cartilage samples were harvested through joint space aspiration. qRT-PCR analysis was performed using primary chondrocytes or medial tibial plateau cartilage, whereas Western blot was conducted on extracts from medial tibial plateau cartilage or primary chondrocytes. Histological evaluation was carried out on tissues from the right knee joint, medial tibial plateau, and medial femoral condyle. Detailed methodological descriptions are provided in subsequent sections. All procedures were approved by the Animal Care and Use Committee of the Shandong First Medical University Affiliated Hospital for Neck, Shoulder, Waist and Leg Pain and conducted in compliance with institutional ethical standards for animal research.

### Chondrocyte culture and treatment

The murine chondrocyte line ATDC5 was obtained from the Cell Bank of the Chinese Academy of Sciences (China). After detachment with trypsin and collection via centrifugation, the cells were resuspended in Roswell Park Memorial Institute-1640 medium (Thermo Fisher Scientific, USA) containing 10% fetal bovine serum (FBS; Gibco, USA) and 1% penicillin–streptomycin. Cell cultures were incubated at 37 °C in a humidified environment with 5% CO₂, and the medium was refreshed every 48 h. A curcumin stock solution was prepared using dimethyl sulfoxide (DMSO) and diluted to achieve final concentrations of 5, 10, 15, and 20 µM [24]. A vehicle control group, treated with an equivalent volume of DMSO corresponding to the highest curcumin concentration, was included to account for potential solvent effects. Following 48 h of treatment exposure, culture supernatants were harvested for further analysis.

To simulate osteoarthritic conditions in vitro, ATDC5 chondrocytes were stimulated with 10 ng/mL IL-1β for 24 h, constituting the IL-1β group. In the intervention group (IL-1β + Curcumin), cells were co-treated with 20 µM curcumin and IL-1β for the same duration. Untreated cells served as the control group.

### Cell transfection

ATDC5 chondrocytes in the logarithmic growth phase were trypsinized to obtain single-cell suspensions and seeded into 6-well plates at a density of 2 × 10⁵ cells per well. Transfection was initiated when cellular confluence reached approximately 70%. The nucleic acid constructs and regulators used included: an EIF4A1 overexpression plasmid (pcDNA3.1-EIF4A1), EIF4A1-specific siRNA (si-EIF4A1), miR-338-3p mimic, miR-338-3p inhibitor, along with their respective controls—empty pcDNA3.1 vector, non-targeting siRNA (si-NC), scrambled mimic (mimic-NC), and scrambled inhibitor (inhibitor-NC). All oligonucleotides and plasmids were sourced from RiboBio (China). Transfection procedures were performed using Lipofectamine™ 3000 (Invitrogen) in accordance with the manufacturer’s guidelines. Following transfection, cells were cultured for an additional 48 h [25]. Transfection efficiency was subsequently assessed through qRT-PCR or Western blot analysis.

### Cytotoxicity assay

Cellular cytotoxicity was evaluated using a Cell Counting Kit-8 (CCK-8; Dojindo, Japan). Transfected ATDC5 chondrocytes—comprising untransfected controls, miR-338-3p overexpressing, miR-338-3p inhibiting, and EIF4A1-silenced groups—were seeded into 96-well plates (Corning, USA) at a density of 5,000 cells per well in 200 µL of culture medium. Following a 24-h incubation to facilitate attachment, 20 µM curcumin was administered to the designated treatment groups. The cells were subsequently cultured for another 48 h. Cytotoxicity assessment was conducted by quantifying absorbance at 450 nm with a microplate reader (Thermo Fisher Scientific) [26]. Each experimental condition was performed in five replicate wells to ensure reproducibility and reduce random error.

Cell viability was calculated using the formula: $\mathrm{Cell}\;\mathrm{viability}\;\left(\%\right)=\left(\mathrm{OD}\;\mathrm{of}\;\mathrm{treated}\;\mathrm{cells}/\mathrm{OD}\;\mathrm{of}\;\mathrm{control}\;\mathrm{cells}\right)\times100$. 

### qRT-PCR

Total RNA was extracted from mouse cartilage tissues and curcumin-treated chondrocytes (ATDC5) using Trizol reagent (Life Technologies, USA) in accordance with the manufacturer’s instructions. Complementary DNA (cDNA) synthesis was performed through two distinct reverse transcription processes: mRNA was reverse transcribed with the M-MLV RTase cDNA Synthesis Kit (Takara, Japan), and miRNA was converted using the Mir-X miRNA First-Strand Synthesis Kit (Takara). Quantitative real-time PCR was carried out on an ABI PRISM 7700 system (Thermo Fisher Scientific) with SYBR GreenER qPCR SuperMix (Thermo Fisher Scientific) for fluorescence detection. Each 25 µL reaction system comprised 0.2 µg cDNA, 10 µM each of forward and reverse primers (designed by Invitrogen and synthesized by Takara, China; Table 1), and an appropriate volume of SuperMix. The amplification protocol included an initial step of 95 °C for 5 min, followed by 40 cycles of 95 °C for 15 s and 60 °C for 45 s. For data normalization, U6 snRNA and glyceraldehyde-3-phosphate dehydrogenase (GAPDH) were used as internal controls for miR-338-3p and EIF4A1 mRNA, respectively. Relative gene expression was calculated via the 2^⁻ΔΔCt^ method, and data analysis was conducted using ABI Prism 7700 SDS Software (Thermo Fisher Scientific) [27].

**Table 1:** Primer Sequences

Gene	Primer Sequence (5’–3’)
miR-338-3p	Forward: 5’-GGGTCCAGCATCAGTGATT-3’
	Reverse: 5’-GCGTTGTGTTGTGTTGTGTT-3’
EIF4A1	Forward: 5’-ATCCCAGAGGCTCTCCTCAC-3’
	Reverse: 5’-CTACCATTTTCTCTCCCCTGCTT-3’
ATF3	Forward: 5’- TTTGCTAACCTGACGCCCTT-3’
	Reverse: 5’- TGACTGATTCCAGCGCAGAG-3’
DDX3X	Forward: 5’- ATATAAGGAAAAACTCAAGTGGTAGCC − 3’
	Reverse: 5’- ATCCAGAGCCCTAGGTGAGG − 3’
JUN	Forward: 5’- TCCAAGTGCCGAAAAAGGAAG -3’
	Reverse: 5’- CGAGTTCTGAGCTTTCAAGGT − 3’
KDELR3	Forward: 5’- CTACAACACAGTAATGAAGGTGG − 3’
	Reverse: 5’- CAGAAGAAACTCCAGGCGGA − 3’
VEGFA	Forward: 5’- GGGCAGAATCATCACGAAGT − 3’
	Reverse: 5’- TGGTGATGTTGGACTCCTCA − 3’
U6	Forward: 5’-CTCGCTTCGGCAGCACA-3’
	Reverse: 5’-AACGCTTCACGAATTTGCGT-3’
GAPDH	Forward: 5’-GGGTGTGAACCACGAGAAAT-3’
	Reverse: 5’-ACTGTGGTCATGAGCCCTTC-3’

*Abbreviation*: *miR-338-3p* microRNA-338-3p, *EIF4A1* Eukaryotic Translation Initiation Factor 4 A Isoform 1, *ATF3* Activating Transcription Factor 3, *DDX3X* DEAD-box Helicase 3, X-linked, *JUN* AP-1 Transcription Factor Subunit, *KDELR3* KDEL Endoplasmic Reticulum Protein Retention Receptor 3, *VEGFA* Vascular Endothelial Growth Factor A, *U6* U6 Small Nuclear RNA, *GAPDH* Glyceraldehyde-3-Phosphate Dehydrogenase

### Western blot analysis

Protein lysates were isolated from chondrocytes (ATDC5) under various experimental conditions with radioimmunoprecipitation assay buffer (Beyotime, China), following the supplier’s instructions. Protein concentration was quantified using a bicinchoninic acid protein assay kit (Beyotime). Equivalent protein quantities were resolved via sodium dodecyl sulfate–polyacrylamide gel electrophoresis and subsequently transferred onto polyvinylidene fluoride membranes. After blocking with 5% skim milk, the membranes were incubated at 4 °C overnight with primary antibodies targeting: Cleaved Caspase-3 (Abcam, ab32042, 1:1000), BCL2-associated X protein (Bax; Abcam, ab32503, 1:1000), B-cell lymphoma 2 (Bcl-2; Abcam, ab182858, 1:1000), MMP-13 (Abcam, ab315267, 1:1000), ADAMTS-5 (Abcam, ab41037, 1:1000), EIF4A1 (Abcam, ab185946, 1:1000), and GAPDH (Abcam, ab8245, 1:1000). Following incubation with primary antibodies, the membranes were washed and probed with horseradish peroxidase (HRP)-labeled secondary antibodies. Protein signal detection was performed using an enhanced chemiluminescence substrate (Thermo Scientific Pierce, USA) [27].

### Enzyme-Linked Immunosorbent Assay (ELISA)

Synovial fluid samples isolated from murine cartilage tissue and culture supernatants harvested from chondrocytes ATDC5 following curcumin treatment were analyzed. Prior to supernatant collection, cells were washed three times with PBS to remove residual exogenous IL-1β and then incubated in fresh medium for 24 h to assess endogenous cytokine secretion. The concentrations of IL-1β (ml037361), IL-6 (ml106838), TNF-α (ml002953), COX-2 (ml106869), and inducible nitric oxide synthase (iNOS) (ml059054) were measured using commercial ELISA kits (EnzymeLink, China) in strict accordance with the manufacturer’s protocols [28].

### Cell cycle and apoptosis analysis by flow cytometry

Chondrocytes ATDC5 were subjected to various treatments, including IL-1β stimulation alone, IL-1β combined with curcumin, as well as transfected cells exposed to IL-1β or IL-1β plus curcumin. After 72 h of culture, cells were harvested and counted. Cell cycle distribution was analyzed using a commercial Cell Cycle Detection Kit (E-CK-A351, Elabscience, China), and apoptosis was evaluated with an Annexin V-FITC/PI Apoptosis Detection Kit (E-CK-A211, Elabscience), both performed according to the manufacturer’s instructions. Samples were analyzed on a BD flow cytometer (Becton, Dickinson and Company, USA), and data were processed using FlowJo software (BD, USA).

### Histomorphometric analysis​

Knee joint specimens from the tibia and femur were decalcified, paraffin-embedded, and sectioned for evaluation of synovial alterations such as synovitis. Safranin-O Fast Green staining (3-H Biomedical, Uppsala, Sweden) was applied to visualize morphological features of articular cartilage in the proximal tibia, following the manufacturer’s recommended protocol. For each sample, six randomly chosen sections within a 200 μm area were subjected to morphometric analysis using a microscope equipped with an image processing system (Carl Zeiss, Germany). Apoptosis in chondrocytes was examined on tissue sections via the TUNEL method with a commercial assay kit (ab206386; Abcam, China). Additionally, hematoxylin and eosin (H&E) staining was performed on sections from all experimental groups. The number of TUNEL-positive cells and H&E-stained chondrocytes was quantified per visual field [29, 30], with three random sections per animal used for statistical evaluation.

Cartilage degradation was evaluated based on the Osteoarthritis Research Society International (OARSI) scoring system, which accounts for lesion depth, area of involvement, and osteophyte formation. The OARSI scale ranges from 0.5 (minimal) to 6 (extensive), with composite scores reflecting overall cartilage damage. Additionally, the Mankin score was applied to evaluate structural integrity (0–6, normal to severe disruption), cellular abnormalities (0–3, normal to marked hypocellularity), and loss of Safranin-O staining (0–4, normal to no staining). To assess synovial inflammation, synovitis scoring was performed on H&E-stained sections according to a standardized histological grading system. This scoring system evaluates three key parameters: synovial lining cell hyperplasia (0–3), stromal cellularity/inflammatory infiltration (0–3), and presence of inflammatory exudate (0–2). The total synovitis score ranges from 0 (normal) to 8 (severe synovitis). All histopathological scoring was conducted independently by five trained evaluators under blinded conditions to ensure objectivity and enhance assessment reliability [31].

### Immunohistochemistry

Immunohistochemical staining was conducted utilizing the CSAII Biotin-Free Tyramide Signal Amplification System (Agilent Technologies, USA). Paraffin-embedded tissue samples were sectioned at a thickness of 5 μm and affixed to glass slides. After deparaffinization with xylene (Beyotime) and subsequent rinsing in phosphate-buffered saline (PBS), endogenous peroxidase activity was suppressed through treatment with 0.3% hydrogen peroxide for 10 min at room temperature. To minimize nonspecific antibody binding, sections were blocked for one h using a solution composed of PBS containing 5% fetal bovine serum and 0.3% Triton X-100. The sections were then incubated overnight at 4 °C with primary antibodies targeting ADAMTS5 (dilution 1:100; ab41037, Abcam) and MMP-13 (dilution 1:150; ab39012, Abcam). Control samples received an isotype-matched nonspecific immunoglobulin (Biocare Medical) under identical conditions. Following PBS washes, an HRP-conjugated secondary antibody (ab6720, Abcam) was applied and allowed to incubate for 60 min at ambient temperature. Chromogenic detection was performed using 3,3′-diaminobenzidine (Vector Labs, USA) as the substrate. Knee joint sagittal sections were visualized under a light microscope at 200 × magnification, and the percentages of cells positive for ADAMTS5 and MMP-13 were quantitatively assessed using BZ-II Analyzer software (Keyence, Japan) [30].

### Dual-luciferase reporter assay

To perform the dual-luciferase reporter assay, PCR-amplified DNA fragments encompassing either the wild-type (WT) or mutated (MUT) miR-338-3p binding site within the EIF4A1 3′UTR were inserted into the psiCHECK-2 vector (Promega, USA), generating the recombinant plasmids EIF4A1-3′UTR-WT and EIF4A1-3′UTR-MUT. Chondrocytes (ATDC5) were co-transfected with these luciferase reporter plasmids along with miR-338-3p mimic or a non-targeting control mimic using Lipofectamine 3000. After 48 h, luminescence signals were quantified with the Dual-Luciferase^®^ Reporter Assay System (E1910, Promega, USA) following the supplier’s guidelines [32].

### RNA Immunoprecipitation (RIP) assay

RNA immunoprecipitation was performed with the Magna RIP™ RNA-Binding Protein Immunoprecipitation Kit (Millipore, USA). Cultured chondrocytes (ATDC5) were lysed in RIP lysis buffer (Solarbio) to prepare whole-cell lysates. The lysates were incubated overnight at 4 °C with magnetic beads conjugated to an anti-Ago2 antibody (Millipore). Control samples included input lysate (total lysate without immunoprecipitation) and normal immunoglobulin G [33]. After immunoprecipitation, RNA was extracted using TRIzol reagent. The relative enrichment of EIF4A1 mRNA and miR-338-3p in the precipitates was subsequently quantified.

### Statistical analysis

Data are expressed as mean ± standard deviation and were analyzed using GraphPad Prism v.10.12 (GraphPad Software, USA). Differences between two groups were assessed using unpaired two-tailed Student’s *t*-test. Comparisons among multiple groups were performed using one-way analysis of variance followed by Tukey’s post hoc test. All data met the assumptions of the statistical tests applied. A two-sided *P*-value < 0.05 was considered statistically significant (* *P* < 0.05, ** *P* < 0.01, *** *P* < 0.001).

## Results

### Curcumin inhibits chondrocyte apoptosis and inflammatory response​

**Fig. 1:**
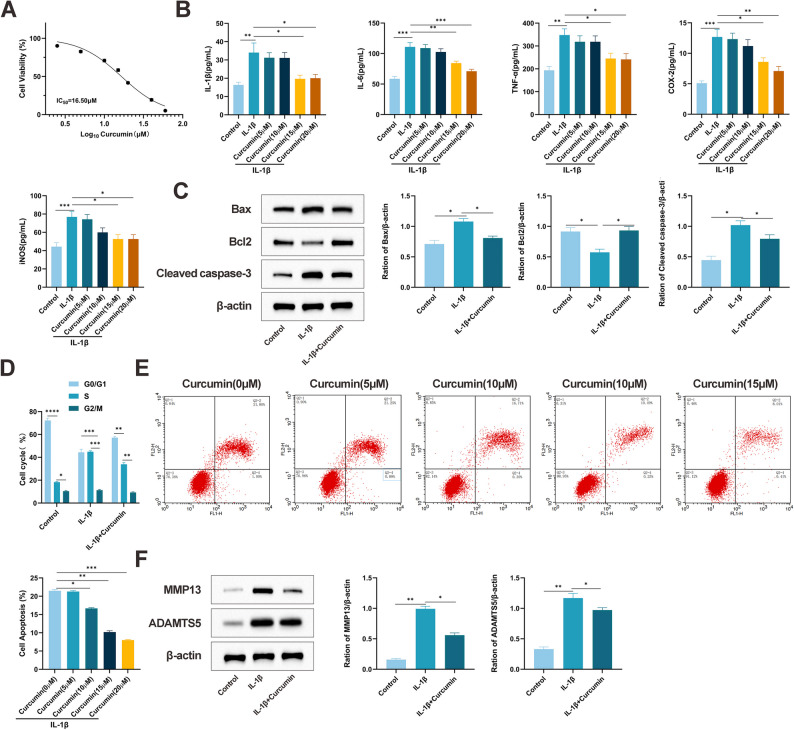
Curcumin alleviates cartilage tissue damage and inhibits chondrocyte apoptosis, inflammatory response, and expression of cartilage-related hydrolases. **A** IC₅₀ of curcumin at different concentrations on IL-1β-stimulated chondrocytes detected by CCK-8 assay. **B** Changes in inflammatory cytokines in IL-1β-stimulated ATDC5 chondrocytes after treatment with different concentrations of curcumin. **C** Expression of apoptosis-related proteins cleaved caspase-3 and Bax in chondrocytes detected by Western blot. **D** Cell cycle changes in chondrocytes detected by flow cytometry. **E** Apoptosis of chondrocytes detected by flow cytometry. **F** Expression levels of the major hydrolases ADAMTS5 and MMP-13 in chondrocytes detected by Western blot. All multiple group comparisons were performed using One-way analysis of variance (One-way ANOVA) followed by Tukey’s post hoc test. Connecting lines in the figure indicate the specific groups being compared. Data are presented as mean ± SD (*n* = 6). * *P* < 0.05, ** *P* < 0.001, *** *P* < 0.0001

To evaluate the therapeutic effects of curcumin on OA, an in vitro model of chondrocyte injury was established by stimulating ATDC5 cells with IL-1β. Cellular viability was examined via CCK-8 assay following treatment with IL-1β and a gradient of curcumin concentrations (0–60 µM). Curcumin treatment resulted in a dose-dependent decrease in viability, with a half-maximal inhibitory concentration (IC₅₀) determined to be 16.50 µM (Fig. 1A). ELISA results further demonstrated that curcumin (5–20 µM) suppressed the secretion of IL-1β-induced inflammatory mediators in a concentration-dependent fashion, with the strongest inhibition occurring at 20 µM (Fig. 1B). Consequently, a concentration of 16.5 µM was selected for further studies. The influence of curcumin on apoptosis was subsequently examined. Western blot analysis indicated that curcumin significantly attenuated IL-1β-induced apoptosis, as evidenced by decreased expression of cleaved caspase-3 and Bax, along with increased Bcl-2 levels (Fig. 1C). Flow cytometric analysis revealed that IL-1β induced G0/G1 phase cell cycle arrest, which was markedly alleviated by curcumin administration (Fig. 1D). Apoptosis assays further confirmed the concentration-dependent suppression of IL-1β-induced chondrocyte apoptosis by curcumin (Fig. 1E). Moreover, Western blot results showed that curcumin downregulated the expression of key cartilage-degrading enzymes, including MMP-13 and ADAMTS5 (Fig. 1F). Together, these results indicate that curcumin protects chondrocytes through the inhibition of inflammatory responses and attenuation of apoptotic pathways.

### Curcumin exerts chondroprotective effects by upregulating miR-338-3p expression

**Fig. 2:**
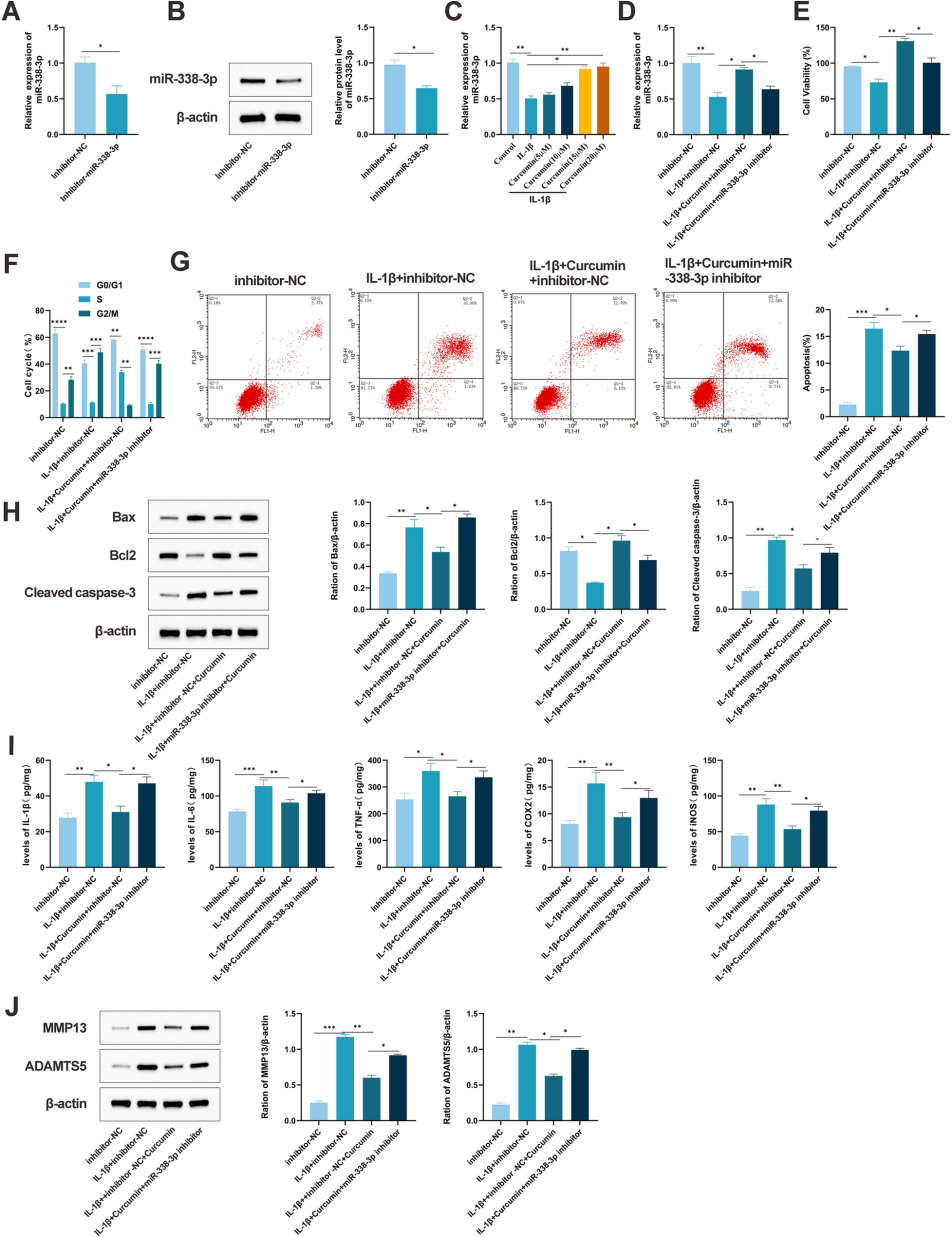
Curcumin upregulates miR-338-3p expression to exert chondroprotective effects, which is reversed by miR-338-3p knockdown. **A**, **B** Transfection efficiency of miR-338-3p inhibitor in chondrocytes detected by qRT-PCR and Western blot. **C** Effect of different concentrations of curcumin on miR-338-3p expression in chondrocytes (ATDC5) detected by qRT-PCR. **D** Effect of miR-338-3p knockdown on miR-338-3p expression in chondrocytes detected by qRT-PCR. **E** Proliferation ability of chondrocytes after miR-338-3p knockdown detected by CCK-8 assay. **F** Cell cycle changes in chondrocytes detected by flow cytometry. **G** Apoptosis of chondrocytes detected by flow cytometry. **H** Changes in apoptosis-related proteins cleaved caspase-3 and Bax in chondrocytes overexpressing miR-338-3p detected by Western blot. **I** Changes in inflammatory cytokines in chondrocytes detected by ELISA. **J** Expression levels of the major hydrolases ADAMTS5 and MMP-13 in chondrocytes detected by Western blot. All multiple group comparisons were performed using One-way analysis of variance (One-way ANOVA) followed by Tukey’s post hoc test. Connecting lines in the figure indicate the specific groups being compared. Data are presented as mean ± SD (*n* = 6). * *P* < 0.05, ** *P* < 0.001, *** *P* < 0.0001

Emerging evidence highlights the involvement of epigenetic regulators, particularly non-coding RNAs such as microRNAs, in the pathogenesis of OA. Previous studies have suggested that miR-338-3p modulates SOX9 expression and suppresses apoptotic processes in IL-1β-stimulated chondrocytes [34]. To further investigate the functional relevance of miR-338-3p in OA, an in vitro injury model was established using IL-1β-treated ATDC5 cells. A chondrocyte model with silenced miR-338-3p expression was initially generated (Fig. 2A, B). Subsequent analysis revealed that curcumin treatment significantly elevated miR-338-3p levels in a dose-dependent manner (Fig. 2C, *P* < 0.05), an effect that was effectively abolished upon transfection with a miR-338-3p inhibitor (Fig. 2D). Functional assessments demonstrated that curcumin markedly enhanced the proliferation of IL-1β-induced chondrocytes (Fig. 2E); however, this pro-proliferative influence was substantially compromised when miR-338-3p was inhibited. Cell cycle analysis via flow cytometry indicated that IL-1β induced G0/G1 phase arrest, which was mitigated by curcumin administration. In contrast, miR-338-3p suppression reinstated the cell cycle arrest phenotype (Fig. 2F). Apoptosis assays further confirmed that curcumin reduced IL-1β-mediated chondrocyte apoptosis, while knockdown of miR-338-3p counteracted this anti-apoptotic activity (Fig. 2G). Consistent with these observations, Western blot analysis showed that curcumin downregulated the expression of the pro-apoptotic proteins cleaved caspase-3 and Bax, and elevated levels of the anti-apoptotic protein Bcl-2. These regulatory effects were reversed upon miR-338-3p inhibition (Fig. 2H). In terms of inflammatory modulation, ELISA results indicated that curcumin significantly suppressed the IL-1β-induced secretion of multiple inflammatory mediators, including IL-1β, IL-6, TNF-α, COX-2, and iNOS (Fig. 2I). This anti-inflammatory action was considerably attenuated when miR-338-3p expression was suppressed. Additionally, Western blot analysis revealed that curcumin downregulated the cartilage catabolic enzymes ADAMTS5 and MMP-13 (Fig. 2J), an effect that was partially reversed under miR-338-3p knockdown conditions. Collectively, these results suggest that curcumin exerts its chondroprotective effects primarily through upregulating miR-338-3p, leading to enhanced cell proliferation, attenuation of inflammatory responses, and preservation of extracellular matrix integrity. Conversely, inhibition of miR-338-3p expression abrogates the beneficial effects of curcumin.

### Overexpression of miR-338-3p synergizes with curcumin to inhibit chondrocyte apoptosis and inflammatory response

**Fig. 3:**
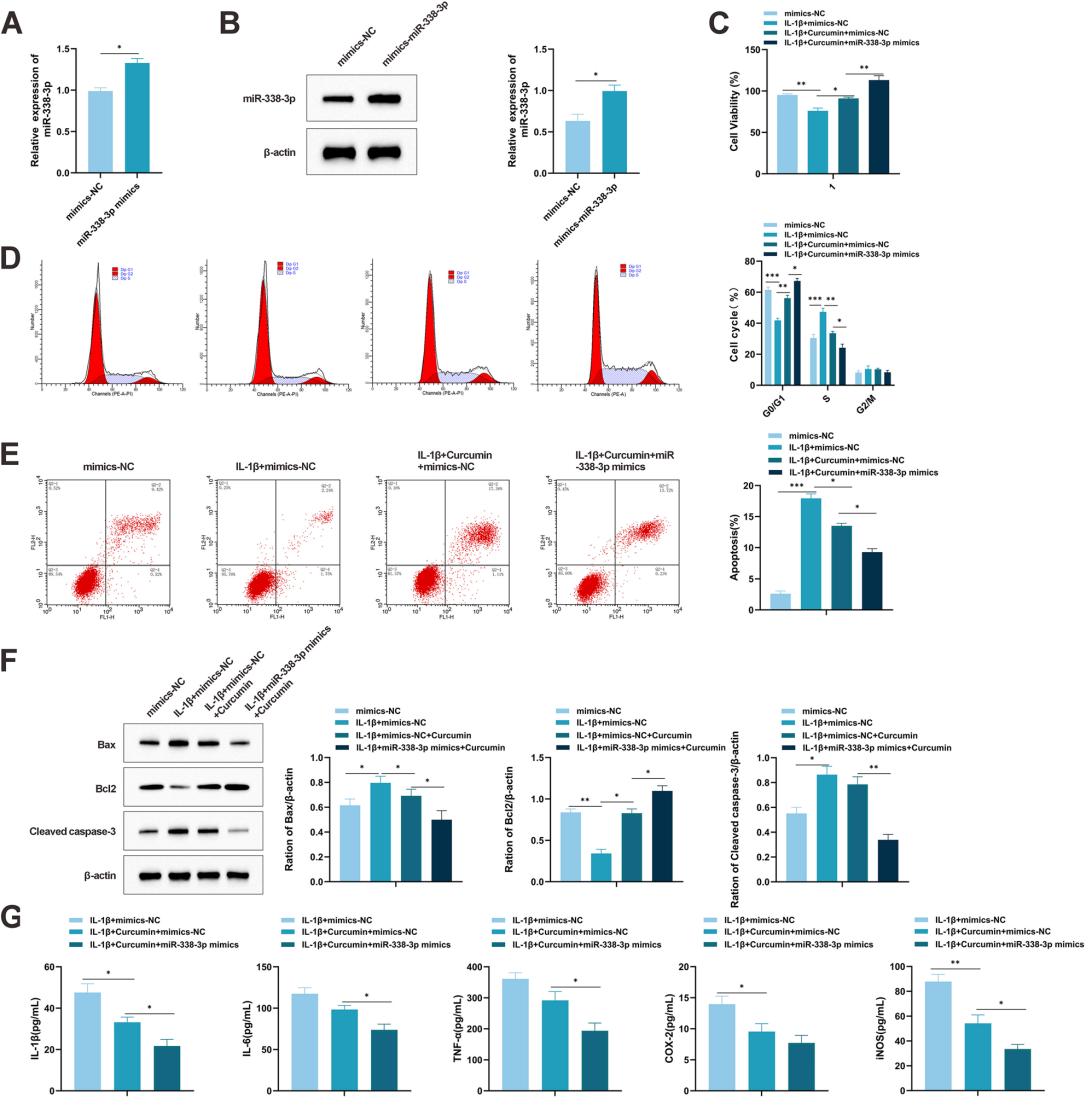
Overexpression of miR-338-3p inhibits apoptosis in cartilage tissue and chondrocytes. **A**, **B** Transfection efficiency of miR-338-3p mimics in chondrocytes (ATDC5) detected by qRT-PCR and Western blot. **C** Viability of chondrocytes overexpressing miR-338-3p detected by CCK-8 assay. **D** Cell cycle changes in chondrocytes detected by flow cytometry. **E** Apoptosis of chondrocytes and miR-338-3p-overexpressing chondrocytes detected by flow cytometry. **F** Changes in apoptosis-related proteins cleaved caspase-3 and Bax in chondrocytes overexpressing miR-338-3p detected by Western blot. **G** Changes in inflammatory cytokines in chondrocytes detected by ELISA. All multiple group comparisons were performed using One-way analysis of variance (One-way ANOVA) followed by Tukey’s post hoc test. Connecting lines in the figure indicate the specific groups being compared. Data are presented as mean ± SD (*n* = 6). * *P* < 0.05, ** *P* < 0.001, *** *P* < 0.0001

To further explore the functional interplay between curcumin and miR-338-3p, miR-338-3p mimics were transfected into IL-1β-stimulated chondrocytes to induce overexpression (Fig. 3A, B). The CCK-8 assay revealed that miR-338-3p mimic transfection enhanced chondrocyte proliferation (Fig. 3C). Furthermore, combined treatment with miR-338-3p mimics and curcumin produced a stronger pro-proliferative effect than each intervention alone. Flow cytometry analysis demonstrated that IL-1β exposure led to G0/G1 phase cell cycle arrest, which was partially reversed by miR-338-3p overexpression. The combination of miR-338-3p mimics and curcumin further alleviated this arrest (Fig. 3D). Apoptosis assays indicated that the co-administration of miR-338-3p mimics and curcumin significantly reduced chondrocyte apoptosis (Fig. 3E). Western blot analysis showed that miR-338-3p mimics downregulated the expression of cleaved caspase-3 and Bax, while upregulating Bcl-2. These modulatory effects on apoptotic markers were enhanced when miR-338-3p mimics were applied together with curcumin (Fig. 3F). ELISA results indicated that the combined treatment synergistically suppressed IL-1β-induced secretion of inflammatory mediators, including IL-1β, IL-6, TNF-α, COX-2, and iNOS (Fig. 3G). In summary, these results demonstrate that miR-338-3p overexpression acts synergistically with curcumin to inhibit apoptosis and attenuate inflammatory responses in chondrocytes and cartilage.

### miR-338-3p targets and regulates EIF4A1

**Fig. 4:**
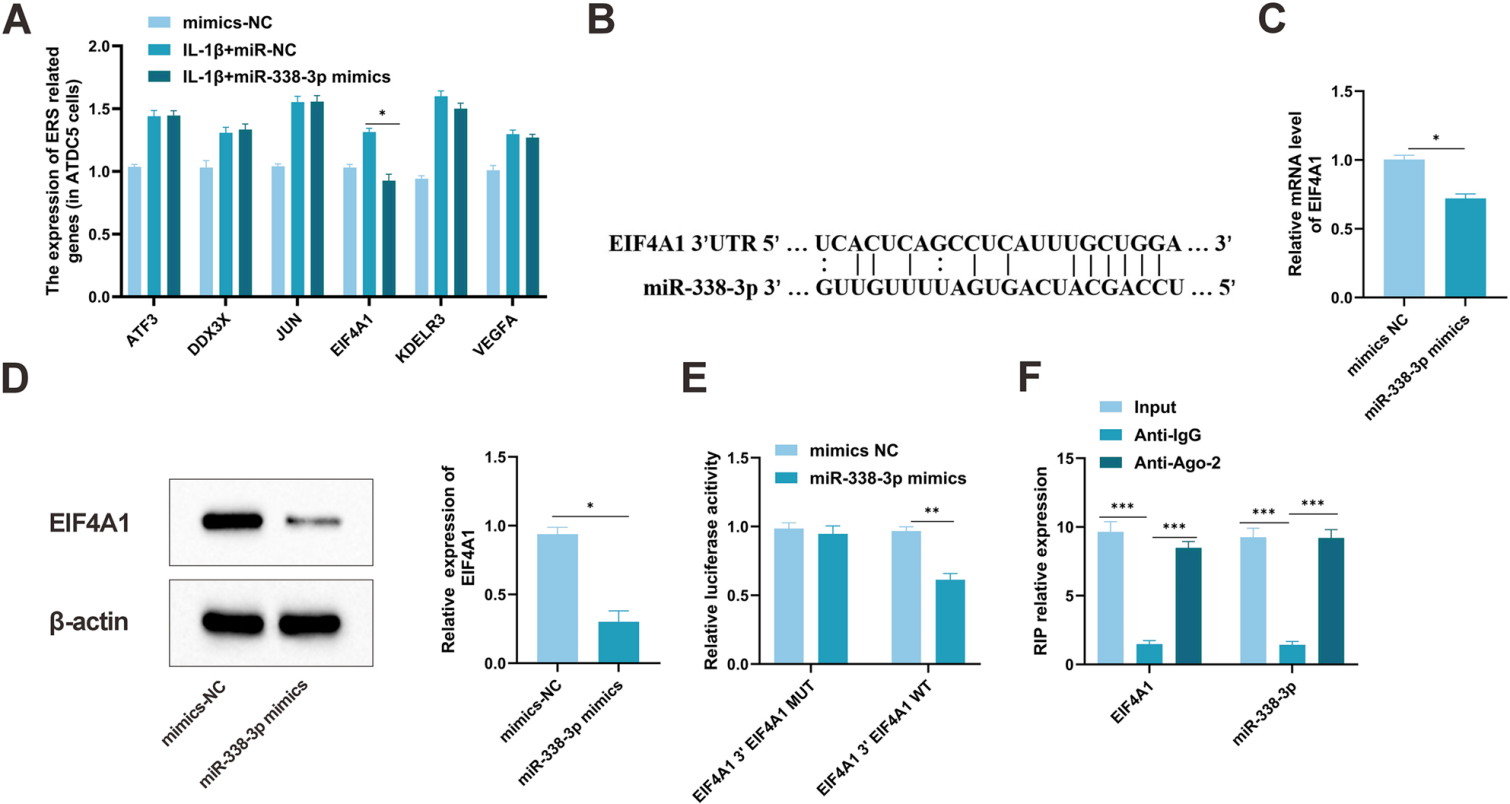
EIF4A1 is a target of miR-338-3p, and miR-338-3p negatively regulates EIF4A1. **A** Expression levels of potential OA-related genes (ATF3, DDX3X, JUN, EIF4A1, KDELR3, VEGFA) in chondrocytes (ATDC5) and miR-338-3p-overexpressing chondrocytes detected by qRT-PCR. **B** Predicted binding site between miR-338-3p and EIF4A1 by TargetScan. **C** EIF4A1 expression in chondrocytes transfected with miR-338-3p mimic detected by qRT-PCR. **D** EIF4A1 expression in chondrocytes transfected with miR-338-3p mimic detected by Western blot. **E** Luciferase activity in chondrocytes co-transfected with miR-338-3p mimic and WT or MT reporter vectors (* *P* < 0.05 vs. miR-NC). **F** Binding interaction between miR-338-3p and EIF4A1 detected by RIP assay. Data are presented as mean ± SD (*n* = 6). * *P* < 0.05, ** *P* < 0.001, *** *P* < 0.0001

Through expression profiling analysis of samples from OA patients and healthy controls in the Gene Expression Omnibus database, six candidate genes associated with endoplasmic reticulum stress were previously identified: Activating Transcription Factor 3, DEAD-Box Helicase 3 X-Linked, AP-1 Transcription Factor Subunit, EIF4A1, KDEL Endoplasmic Reticulum Protein Retention Receptor 3, and Vascular Endothelial Growth Factor A (VEGFA) [35]. To examine whether these genes are regulated by miR-338-3p, qRT-PCR was conducted in IL-1β-stimulated chondrocytes overexpressing miR-338-3p. The data indicated that elevated miR-338-3p specifically reduced EIF4A1 mRNA levels (*P* < 0.01), without affecting the expression of the other five genes (Fig. 4A), supporting EIF4A1 as a selective target of miR-338-3p.

Bioinformatic prediction using StarBase revealed a putative binding site for miR-338-3p within the 3′UTR of EIF4A1 (Fig. 4B). Dual-luciferase reporter assays further confirmed that miR-338-3p overexpression markedly suppressed luciferase activity from the wild-type EIF4A1 reporter construct (*P* < 0.01), while no significant effect was observed with the mutant version (Fig. 4C–E). RIP assays showed specific enrichment of both miR-338-3p and EIF4A1 mRNA by Ago2 antibody immunoprecipitation (Fig. 4F), suggesting their direct association within the RNA-induced silencing complex. These results indicate that miR-338-3p directly binds to EIF4A1 and represses its expression.

### EIF4A1 Is a key downstream effector in the chondroprotective effects of curcumin

**Fig. 5:**
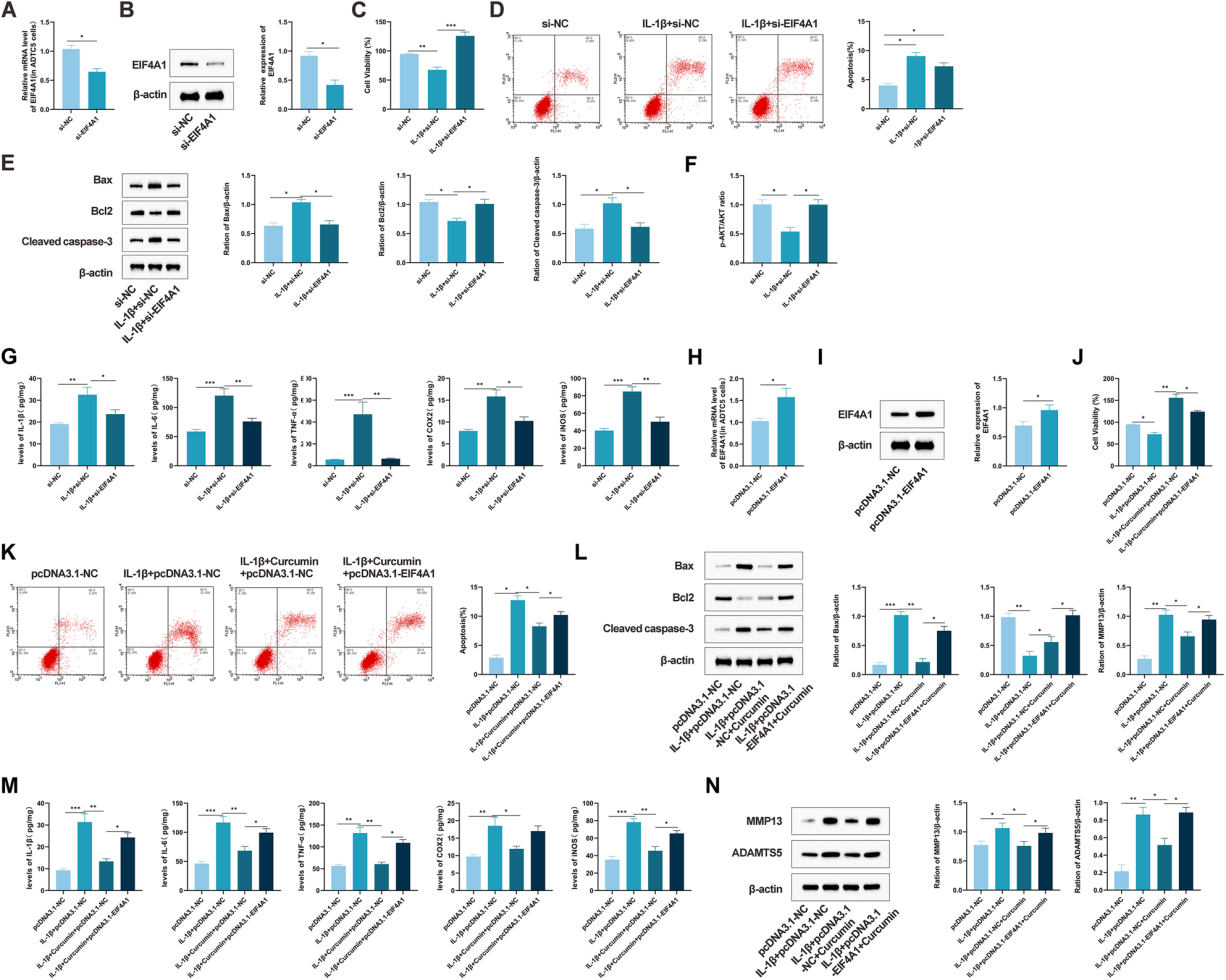
EIF4A1 may function as a key downstream effector in the chondroprotective mechanism of curcumin. **A**, **B** Transfection efficiency of EIF4A1 knockdown in chondrocytes (ATDC5) detected by qRT-PCR and Western blot. **C** Proliferation of EIF4A1-knockdown chondrocytes detected by CCK-8 assay. **D** Apoptosis of EIF4A1-knockdown chondrocytes detected by flow cytometry. **E** Expression of apoptosis-related proteins cleaved caspase-3, Bax, and Bcl-2 in chondrocytes detected by Western blot. **F** Western blot analysis of the effect of EIF4A1 knockdown on the phosphorylation level of AKT (p-AKT/AKT ratio) induced by IL-1β. **G** Changes in inflammatory cytokine levels in chondrocytes detected by ELISA. **H** EIF4A1 protein levels in chondrocytes detected by Western blot. **I** EIF4A1 expression in chondrocytes detected by qRT-PCR. **J** Proliferation of EIF4A1-overexpressing chondrocytes detected by CCK-8 assay. **K** Apoptosis of EIF4A1-overexpressing chondrocytes detected by flow cytometry. **L** Expression of apoptosis-related proteins cleaved caspase-3, Bax, and Bcl-2 in EIF4A1-overexpressing chondrocytes detected by Western blot. **M** Changes in inflammatory cytokine levels in chondrocytes detected by ELISA. **N** Expression levels of the hydrolase MMP-13 (collagenase-3) and disaggregase ADAMTS5 in chondrocytes detected by Western blot. All multiple group comparisons were performed using One-way analysis of variance (One-way ANOVA) followed by Tukey’s post hoc test. Connecting lines in the figure indicate the specific groups being compared. Data are presented as mean ± SD (*n* = 6). * *P* < 0.05, ** *P* < 0.001, *** *P* < 0.0001

To investigate the functional involvement of EIF4A1 in the chondroprotective actions of curcumin, EIF4A1 expression was silenced in chondrocytes using siRNA (Fig. 5A, B). According to CCK-8 assay results, silencing of EIF4A1 promoted the proliferation of IL-1β-stimulated chondrocytes (Fig. 5C). Flow cytometric analysis further indicated that EIF4A1 silencing reduced IL-1β-induced apoptosis (Fig. 5D). Western blot revealed that EIF4A1 knockdown led to decreased expression of cleaved caspase-3 and Bax, along with increased Bcl-2 levels in IL-1β-treated cells (Fig. 5E). Given that previous studies suggest EIF4A1 facilitates Caspase-3 activation via suppression of AKT phosphorylation, we further examined the status of the PI3K/AKT pathway. As shown in Fig. 5E, EIF4A1 knockdown significantly restored the phosphorylation level of AKT (p-AKT/AKT ratio) which had been suppressed by IL-1β, mimicking the effect of curcumin treatment (Fig. 5F). ELISA results also showed that inhibition of EIF4A1 significantly reduced the secretion of inflammatory mediators—including IL-1β, IL-6, TNF-α, COX-2, and iNOS—triggered by IL-1β (Fig. 5G, *P* < 0.01). These effects were consistent with those observed following curcumin treatment. Conversely, an EIF4A1 overexpression model was generated by transfecting chondrocytes with the pcDNA3.1-EIF4A1 plasmid (Fig. 5H, I). Under these conditions, upregulation of EIF4A1 completely reversed the pro-proliferative effect of curcumin in IL-1β-stimulated chondrocytes (Fig. 5J). Flow cytometry analysis indicated that EIF4A1 overexpression increased apoptosis and counteracted the anti-apoptotic benefits of curcumin (Fig. 5K). Western blot analysis confirmed that ectopic EIF4A1 expression abolished the regulatory influence of curcumin on apoptosis-related proteins (Fig. 5L). Similarly, although curcumin suppressed inflammatory cytokine release, overexpression of EIF4A1 significantly attenuated this anti-inflammatory effect (Fig. 5M). Furthermore, Western blot results showed that elevated EIF4A1 expression restored the activities of MMP-13 and ADAMTS5—key enzymes responsible for cartilage matrix degradation—thereby reducing the therapeutic efficacy of curcumin (Fig. 5N). Collectively, these findings indicate that EIF4A1 serves as an essential downstream mediator in the chondroprotective mechanisms of curcumin.

### Curcumin alleviates cartilage tissue damage in OA mice via the miR-338-3p/EIF4A1 axis

**Fig. 6:**
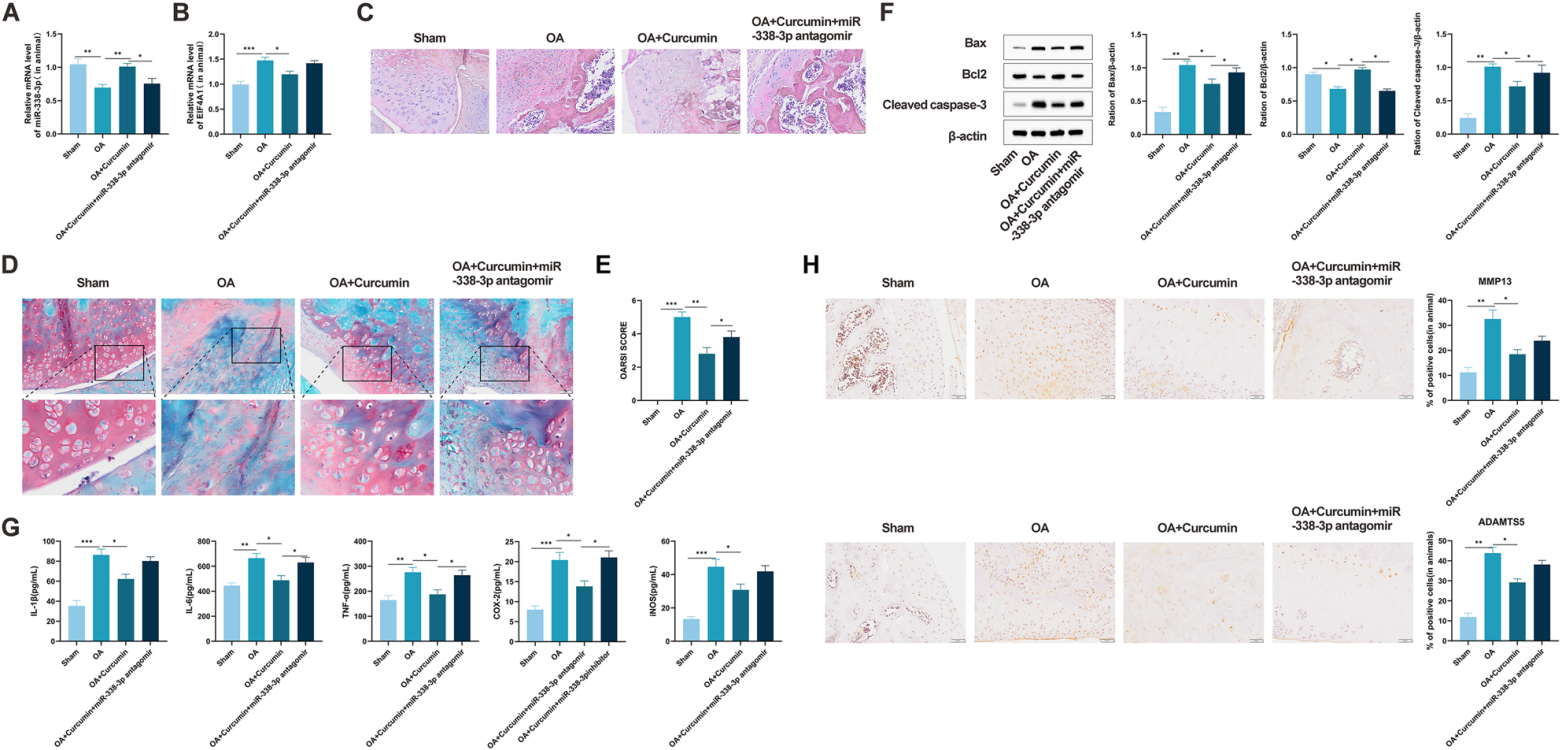
Curcumin alleviates cartilage tissue damage in OA mice via the miR-338-3p/EIF4A1 axis. **A** miR-338-3p expression in cartilage tissue detected by qRT-PCR (*n* = 5). **B** EIF4A1 expression in cartilage tissue detected by qRT-PCR (*n* = 5). **C** Cartilage damage in OA mice observed by H&E staining (*n* = 5). **D** Cartilage damage in OA mice observed by Safranin-O staining. **E** OARSI scores (*n* = 5). **F** Synovitis scores based on H&E staining evaluating synovial lining hyperplasia and inflammatory infiltration (*n* = 5). **G** Expression of apoptosis-related proteins cleaved caspase-3, Bax, and Bcl-2 in cartilage tissue detected by Western blot (*n* = 3). **H** Changes in inflammatory cytokine levels in cartilage tissue detected by ELISA (*n* = 6). **I** Expression levels of the hydrolase MMP-13 (collagenase-3) and disaggregase ADAMTS5 in cartilage tissue detected by immunohistochemistry (*n* = 3). All multiple group comparisons were performed using One-way analysis of variance (One-way ANOVA) followed by Tukey’s post hoc test. Connecting lines in the figure indicate the specific groups being compared. Data are presented as mean ± SD. * *P* < 0.05, ** *P* < 0.001, *** *P* < 0.0001

Based on in vitro findings indicating that curcumin protects chondrocytes from IL-1β-induced injury through the miR-338-3p/EIF4A1 pathway, this mechanism was further examined in a murine OA model. Systematic evaluation of the miR-338-3p/EIF4A1 axis revealed reduced miR-338-3p expression and elevated EIF4A1 levels in OA cartilage. Curcumin administration increased miR-338-3p and decreased EIF4A1, effects that were abolished by miR-338-3p antagomir co-treatment (Fig. 6A, B). To assess the therapeutic influence of curcumin on OA, cartilage pathology was evaluated using H&E and Safranin-O staining. Compared with Sham controls, OA model mice displayed substantial cartilage surface erosion, disorganized cellular architecture, and irregular matrix staining (Fig. 6C). Curcumin treatment significantly improved these pathological alterations, attenuating surface damage and enhancing Safranin-O intensity, whereas the OA + Curcumin + miR-338-3p antagomir group exhibited exacerbated cartilage degradation (Fig. 6D). OARSI scoring further validated that curcumin mitigated OA progression, and this beneficial outcome was impeded by miR-338-3p antagomir (Fig. 6E). Notably, curcumin administration significantly reduced the synovitis score, indicating a suppression of synovial lining hyperplasia and inflammatory infiltration. However, this anti-synovitic effect was significantly reversed in mice co-treated with the miR-338-3p antagomir (Fig. 6F). Western blot analysis indicated that curcumin considerably inhibited apoptosis in OA cartilage, shown by reduced cleaved caspase-3 and Bax, and elevated Bcl-2 expression. These changes were reversed upon miR-338-3p antagomir administration (Fig. 6G). ELISA results demonstrated that the suppressive effect of curcumin on inflammatory mediators—including IL-1β, IL-6, TNF-α, COX-2, and iNOS—was significantly attenuated by miR-338-3p antagomir (Fig. 6H). Immunohistochemical staining revealed that curcumin downregulated the expression of the cartilage-degrading enzymes ADAMTS5 and MMP-13, an effect that was likewise negated by miR-338-3p antagomir (Fig. 6I). Thus, curcumin attenuates cartilage damage in OA mice by modulating the miR-338-3p/EIF4A1 pathway.

## Discussion

OA represents a prevalent degenerative joint disorder, pathologically marked by the gradual degradation of articular cartilage and increased apoptosis of chondrocytes [36]. Current therapeutic strategies in the clinic mainly focus on alleviating symptoms, with no effective interventions available to arrest the progression of the disease [37]. In recent years, natural products have garnered significant research interest due to their multi-target actions and high biocompatibility. Curcumin, in particular, has attracted attention for its notable anti-inflammatory and antioxidative properties [38, 39]. Previous studies have indicated that curcumin alleviates OA progression through multiple mechanisms, including inhibition of NF-κB signaling to reduce the expression of inflammatory cytokines such as IL-1β and TNF-α [8, 12, 13]; activation of the Nrf2/HO-1 pathway to attenuate oxidative damage [40, 41]; and modulation of Wnt/β-catenin signaling to maintain cartilage homeostasis. However, most existing research has focused on protein-centric mechanisms, leaving the role of miRNA-mediated epigenetic regulation relatively unclear. Notably, curcumin has been recognized as a potential Pan-Assay Interference Compound (PAINS) due to its ability to interact with a broad range of proteins and signaling pathways. Therefore, its chondroprotective effects are unlikely to rely on a single molecular route. The current study demonstrates, for the first time, that curcumin reduces chondrocyte and cartilage apoptosis, inhibits the release of inflammatory mediators (IL-1β, IL-6, TNF-α) and matrix-degrading enzymes (MMP13, ADAMTS5), and ameliorates OA pathology—as reflected by decreased OARSI scores in a murine model—in part via the miR-338-3p/EIF4A1 regulatory axis. These findings align with earlier reports by Chen et al.., which described the protective effects of curcumin through suppression of oxidative and apoptotic mechanisms [42]. The present study further expands this understanding by uncovering a novel epigenetic mechanism centered on miR-338-3p/EIF4A1 signaling.

miRNAs are small non-coding RNA molecules, typically 17–25 nucleotides in length, that function as post-transcriptional regulators of gene expression. They bind to complementary sequences within the 3′ UTRs of target mRNAs, resulting in either translational inhibition or mRNA degradation [43]. Recent studies have shown that miRNAs participate in regulating inflammatory pathways activated in various joint diseases, regardless of whether they are induced by crystal deposition [44–46]. For instance, miR-322 and miR-671-3p inhibit cartilage matrix degradation and alleviate OA cartilage damage by suppressing TRAF3 [47, 48]. MicroRNA-145 reduces TNF-α-driven cartilage matrix breakdown and mitigates OA cartilage destruction by directly inhibiting MKK4 [49]. Moreover, miRNAs may target TLRs, IL-1β, chemokines, and NLRP3 to ameliorate gouty arthritis [50]. Eukaryotic initiation factor 4 A (eIF4A) is critically involved in the initiation of translation through its interaction with the 5′ untranslated region (5′ UTR) of mRNA transcripts [51]. The two primary isoforms, eIF4A1 and eIF4A2, exhibit a high degree of sequence homology. According to Gene Ontology and Kyoto Encyclopedia of Genes and Genomes enrichment analyses, EIF4A1 has been further implicated in the pathogenic mechanisms underlying OA [35]. However, few studies have explored the relationship between EIF4A1 and OA. Therefore, this study focuses on miRNA and EIF4A1 as key points to investigate the roles of miR-338-3p and EIF4A1 in OA.

This investigation reveals that curcumin alleviates OA in part via the miR-338-3p/EIF4A1 pathway. In murine models, osteoarthritic cartilage exhibited substantially reduced miR-338-3p expression and elevated EIF4A1 levels. Curcumin intervention effectively reversed these changes, upregulating miR-338-3p and downregulating EIF4A1. In vitro, curcumin exerted dose-dependent anti-apoptotic effects on ATDC5 chondrocytes, with higher concentrations yielding more pronounced cytoprotection. Luciferase reporter assays verified that miR-338-3p directly binds to and inhibits EIF4A1. Both miR-338-3p overexpression and EIF4A1 knockdown enhanced chondrocyte proliferation and suppressed apoptosis. In OA model mice, curcumin administration and miR-338-3p agomir injection attenuated oxidative stress, improved histological scores assessed by H&E and Safranin O staining, and reduced cartilage degradation. These observations align with prior studies indicating miR-338-3p protects against OA by reducing oxidative damage and apoptosis [17], while the present work further establishes a novel association with curcumin. Moreover, miR-338-3p overexpression acted synergistically with curcumin to ameliorate OA, a finding consistently supported by in vitro results. Collectively, these data indicate that curcumin suppresses chondrocyte apoptosis, enhances proliferation, and reduces the expression of inflammatory mediators (IL-6, TNF-α) and matrix-degrading enzymes (MMP-13, ADAMTS5) at least partially through activation of miR-338-3p and inhibition of EIF4A1, ultimately mitigating OA progression. In contrast to previous reports, this study is the first to identify curcumin-mediated upregulation of miR-338-3p and delineate the functional miR-338-3p/EIF4A1 axis using complementary in vivo and in vitro approaches. While consistent with Zhang et al..’s report on the chondroprotective function of miR-338-3p [52] the current findings provide mechanistic insight into one important downstream signaling through which curcumin exerts its therapeutic effects.

It is critical to emphasize that curcumin is a pleiotropic agent with well-documented inhibitory effects on multiple inflammatory and catabolic pathways, including direct inhibition of COX-2 activity and suppression of NF-κB signaling, both of which are strongly implicated in OA pathogenesis [53, 54]. These established mechanisms likely act in parallel and/or cooperatively with the miR-338-3p/EIF4A1 axis identified in this study. The present work does not claim that the miR-338-3p/EIF4A1 pathway is the sole or dominant mediator of curcumin’s activity, but rather identifies it as a novel and functionally relevant epigenetic route that contributes to its overall chondroprotective efficacy.

Although our study clearly establishes that curcumin treatment leads to increased levels of miR-338-3p, the precise upstream mechanism governing this upregulation warrants further discussion. Curcumin is a well-known pleiotropic molecule capable of modulating various transcription factors, including NF-κB, Nrf2, and p53, which are critical regulators of gene expression. It is plausible that curcumin promotes the transcription of the host gene of miR-338-3p by activating specific transcription factors that bind to its promoter region. For instance, given curcumin’s established role in inhibiting NF-κB and activating Nrf2 [55], these factors might directly or indirectly enhance the transcriptional activity of the miR-338 locus. Alternatively, curcumin could influence the post-transcriptional processing of miR-338-3p, potentially by modulating the activity of Drosha or Dicer enzymes [56], thereby increasing the maturation efficiency of pre-miR-338-3p into its functional form. While our current data confirm the functional outcome of miR-338-3p elevation, future experiments employing promoter activity assays and chromatin immunoprecipitation (ChIP) will be essential to pinpoint the exact transcription factors involved. Additionally, investigating the expression levels of primary miR-338-3p transcripts and processing enzymes under curcumin treatment would help distinguish between transcriptional activation and processing enhancement. Addressing these upstream mechanisms will provide a more comprehensive understanding of how curcumin orchestrates the miR-338-3p/EIF4A1 axis.

A notable discrepancy exists between the curcumin concentrations used in our in vitro and in vivo models, which warrants careful consideration. In our in vitro experiments, an IC₅₀ of 16.5 µM was determined, and subsequent mechanistic studies utilized concentrations within this micromolar range (e.g., 20 µM) to achieve significant cytoprotective effects. In contrast, the in vivo study administered curcumin orally at 50 mg/kg. It is well-documented that curcumin suffers from poor oral bioavailability due to low absorption, rapid metabolism, and systemic elimination, making it highly unlikely that oral administration alone achieves sustained micromolar concentrations in the synovial fluid or serum comparable to those used in cell culture [57]. This pharmacokinetic barrier presents a challenge in directly translating in vitro efficacy to in vivo outcomes. While we observed robust therapeutic effects in the murine OA model, including the modulation of the miR-338-3p/EIF4A1 axis and improvement in OARSI scores, the actual concentration of curcumin or its active metabolites reaching the joint cavity remains unquantified in this study. It is possible that the local accumulation of curcumin in inflamed tissues, the synergistic effects of its metabolites, or a heightened sensitivity of the miR-338-3p pathway to lower physiological concentrations may contribute to the observed in vivo efficacy despite low systemic bioavailability. Nevertheless, the lack of direct measurement of curcumin levels in mouse serum or synovial fluid is a limitation of this work. Future studies employing advanced delivery systems (e.g., nanoparticles, liposomes) to enhance bioavailability, coupled with pharmacokinetic profiling to quantify intra-articular curcumin concentrations, will be crucial to definitively bridge the gap between the micromolar doses effective in vitro and the achievable levels in vivo.

One potential concern regarding targeting EIF4A1 is its role as a general translation initiation factor; theoretically, its suppression could impair the synthesis of essential matrix proteins like Collagen II and Aggrecan [58]. However, emerging evidence suggests that mRNA dependence on EIF4A1 is heterogeneous. Transcripts with long, GC-rich, and highly structured 5’ UTRs—common in pro-apoptotic (e.g., Caspase-3, Bax) and pro-inflammatory genes—are disproportionately sensitive to EIF4A1 levels [59, 60]. In contrast, many structural matrix genes likely possess simpler 5’ UTRs, rendering them less dependent on high EIF4A1 activity. Our data supports this selectivity: while EIF4A1 knockdown significantly reduced pro-apoptotic proteins and catabolic enzymes, it concurrently enhanced chondrocyte proliferation and, crucially, led to significant preservation of cartilage matrix and proteoglycans in vivo (Fig. 6D, E). This indicates that the partial modulation of EIF4A1 achieved by curcumin/miR-338-3p preferentially suppresses pathogenic factors without compromising the global protein synthesis required for matrix maintenance.

Current medical research suggests that OA involves not only mechanical wear but also catabolic enzyme-mediated degradation of the cartilage matrix—primarily through MMPs and ADAMTS—as well as elevated levels of inflammatory cytokines including IL-1β, IL-6, and TNF-α [61, 62]. Pathological evaluation in OA mouse models demonstrated that both curcumin administration and miR-338-3p overexpression led to reduced OARSI scores, enhanced proteoglycan preservation (as observed via Safranin O staining), downregulation of MMP13, ADAMTS5, cleaved caspase-3, Bax, and EIF4A1, and upregulation of Bcl-2. ELISA analysis indicated higher concentrations of IL-1β, IL-6, and TNF-α in the OA model group, which were significantly suppressed following treatment with curcumin and miR-338-3p agomir. These changes correlated with diminished cartilage damage and ameliorated OA symptoms. In vitro studies using an IL-1β-induced chondrocyte injury model further supported these findings, illustrating that curcumin elevates miR-338-3p expression, thereby inhibiting EIF4A1, attenuating cytotoxicity, and enhancing chondrocyte viability. In summary, these results imply that curcumin mitigates OA pathology via multiple complementary pathways, of which the miR-338-3p/EIF4A1 signaling axis represents a key contributor.

The consistency between animal and cellular experimental results enhances the reliability of the conclusions. This study supports the development of therapeutic strategies based on miR-338-3p mimics or EIF4A1 inhibitors. We confirm that all in vitro experiments were conducted using the ATDC5 chondrocyte cell line. The validation of the key findings (particularly the curcumin/miR-338-3p/EIF4A1 axis) in primary mouse chondrocytes will further enhance their clinical relevance. Regarding this limitation, we plan to verify our core conclusions in future studies by isolating primary articular chondrocytes from mouse models. However, the phosphorylation status of key proteins in the Wnt/β-catenin pathway (β-catenin, GSK-3β) was not thoroughly investigated. Additionally, as highlighted above, future research must prioritize optimizing curcumin delivery systems to overcome its poor bioavailability and include rigorous pharmacokinetic assessments to correlate intra-articular drug concentrations with the observed epigenetic modifications. Furthermore, as discussed above, elucidating the specific transcriptional or post-transcriptional mechanisms by which curcumin upregulates miR-338-3p remains a critical avenue for future research. Further investigations are warranted.

## Conclusion

In summary, curcumin intervention significantly upregulates miR-338-3p expression and downregulates EIF4A1 in an OA context. Curcumin treatment, overexpression of miR-338-3p, and silencing of EIF4A1 collaboratively inhibit chondrocyte apoptosis and promote cell proliferation. miR-338-3p acts as a negative regulator of EIF4A1, and curcumin modulates inflammatory factor secretion in both cartilage tissue and chondrocytes through the miR-338-3p/EIF4A1 pathway, leading to reduced OARSI scores and mitigation of OA progression. These results enhance the understanding of OA pathogenesis and highlight the miR-338-3p/EIF4A1 axis as a promising therapeutic target, which can be pharmacologically influenced by curcumin.

## Supplementary Information

Supplementary Material 1.

## Data Availability

Data is available from the corresponding author on request.
