# Supplementary figures and images for: Curcumin inhibits chondrocyte apoptosis and inflammation in osteoarthritis via the miR-338-3p/EIF4A1 signaling axis
Source: Hereditas. 2026 Apr 9;163:63. doi: 10.1186/s41065-026-00674-x (PMC13181902; doi:10.1186/s41065-026-00674-x)

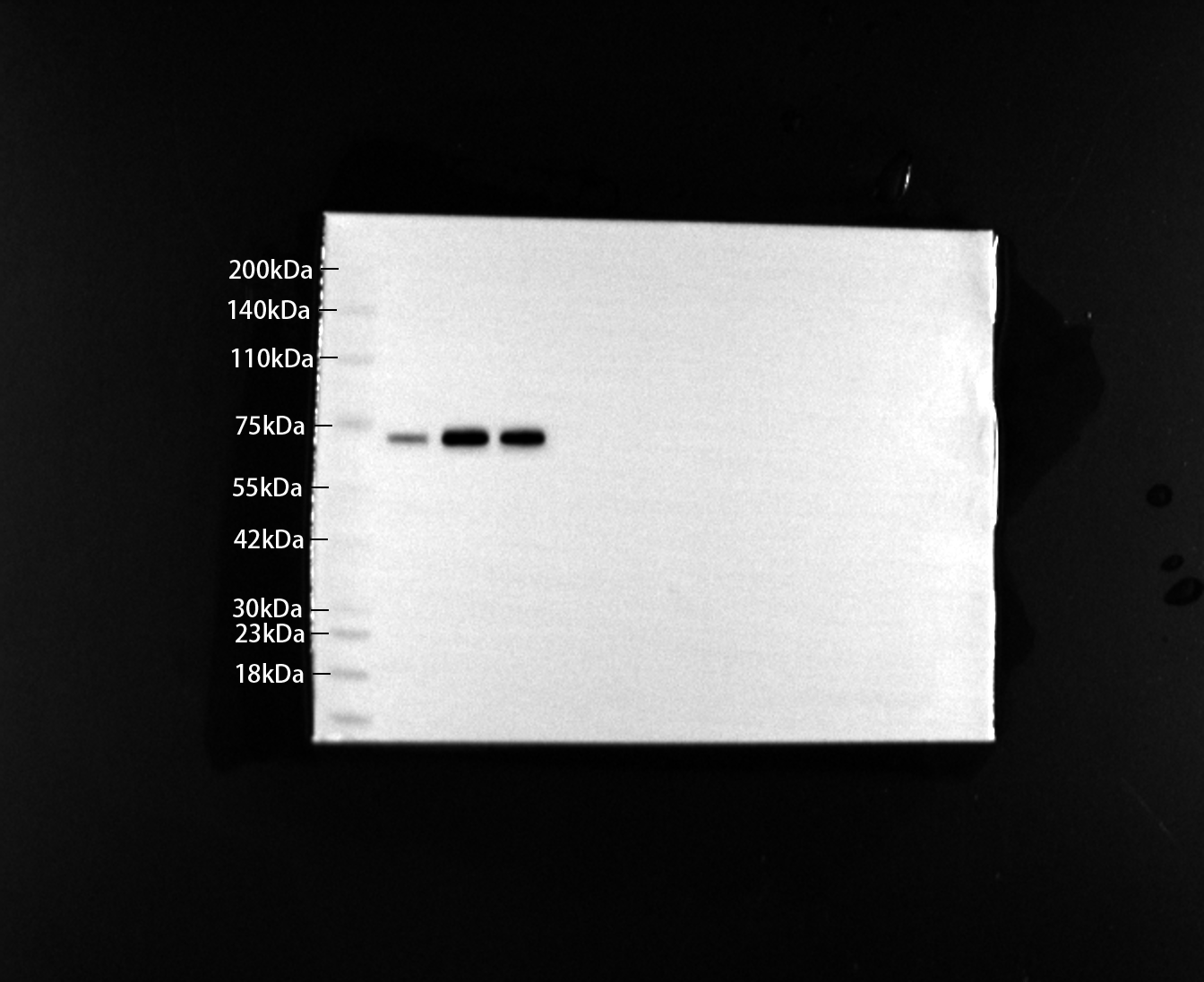

Supplement: Supplementary file 1 — Supplementary Material 1. [file 41065_2026_674_MOESM1_ESM.zip › Original image for western blot -marker/Original image Figure 1F/1F-ADAMTS5.tif]

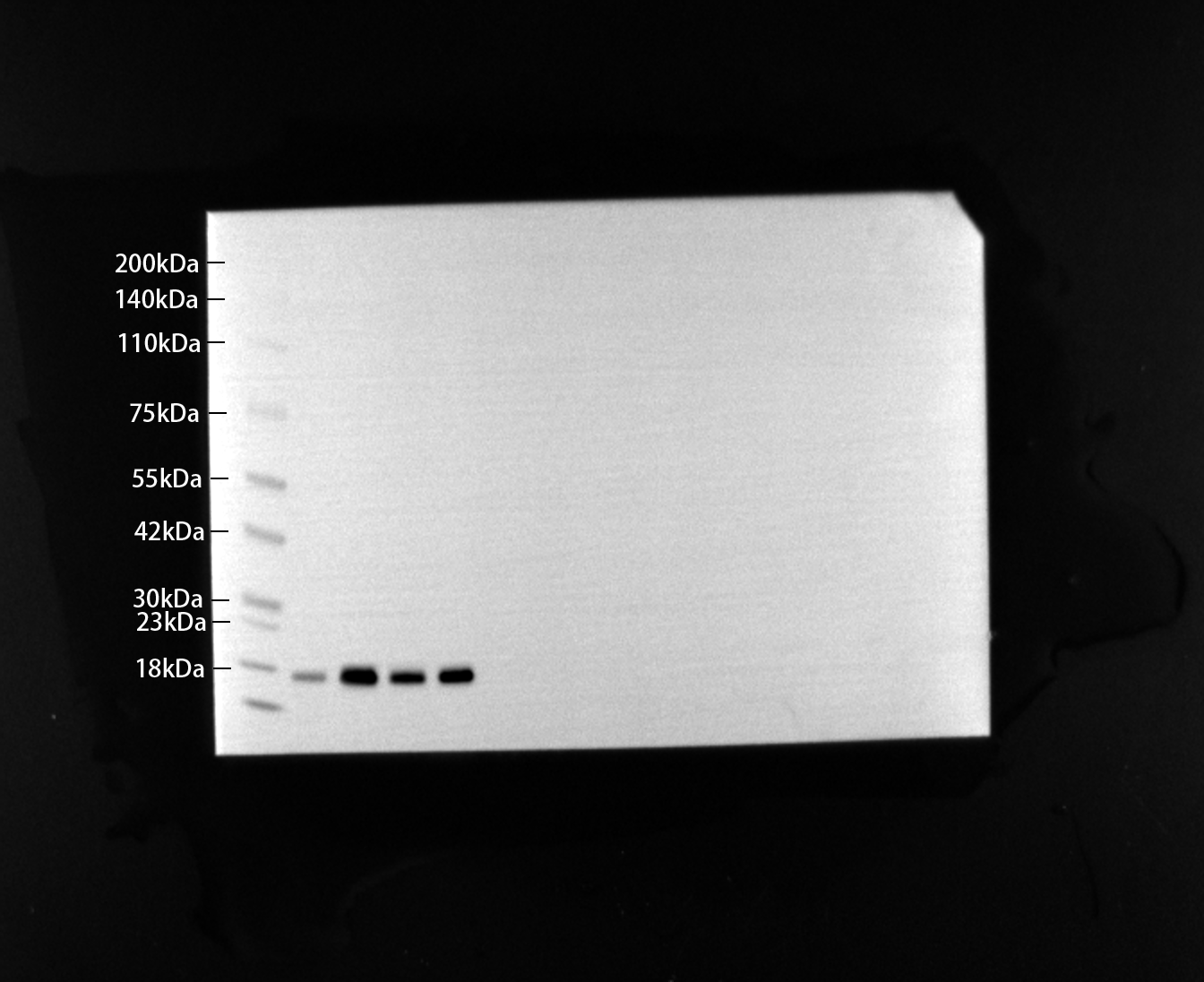

Supplement: Supplementary file 1 — Supplementary Material 1. [file 41065_2026_674_MOESM1_ESM.zip › Original image for western blot -marker/Original image Figure 6F/6F-Cleaved caspase-3.tif]

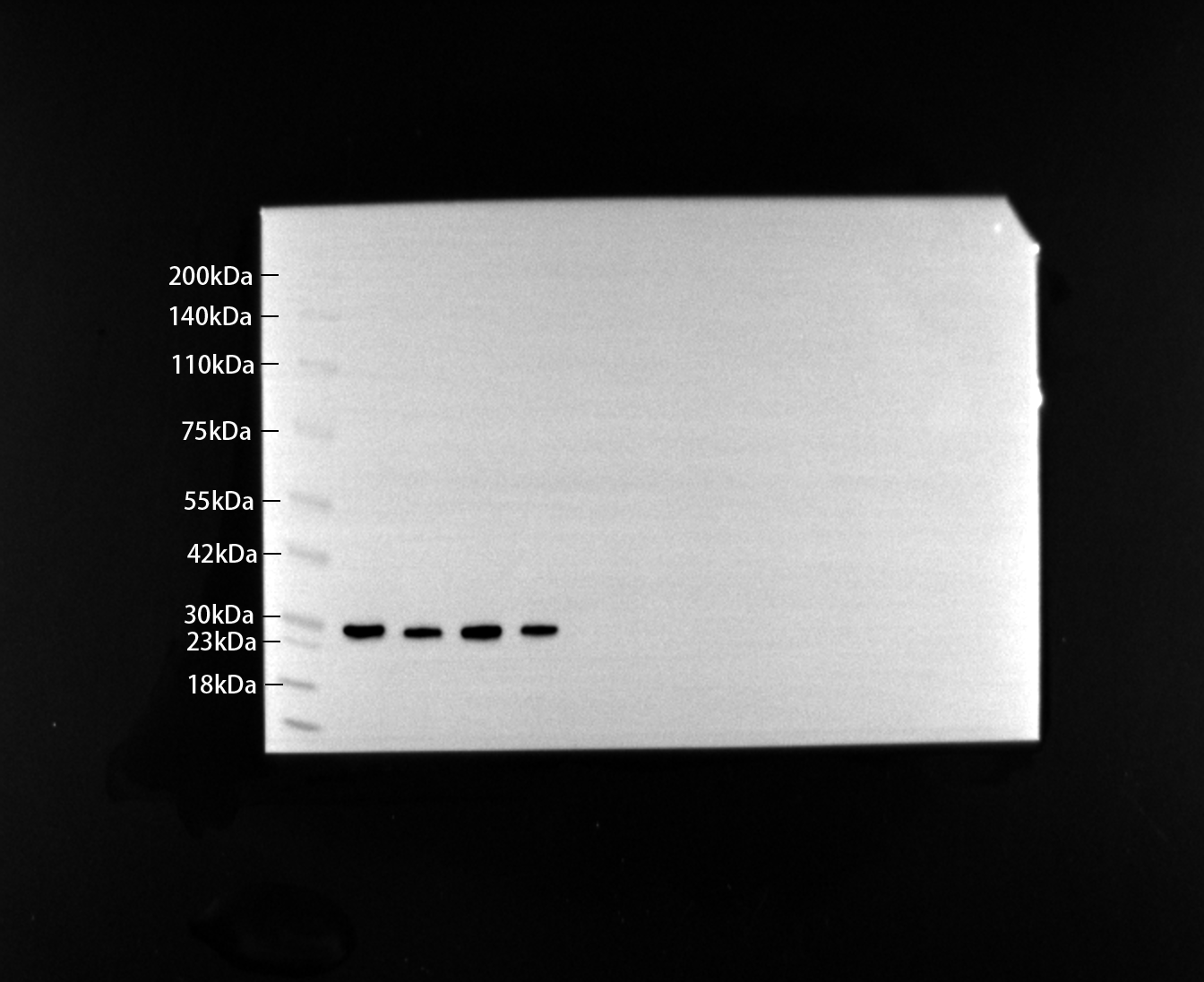

Supplement: Supplementary file 1 — Supplementary Material 1. [file 41065_2026_674_MOESM1_ESM.zip › Original image for western blot -marker/Original image Figure 6F/6F-BcI2.tif]

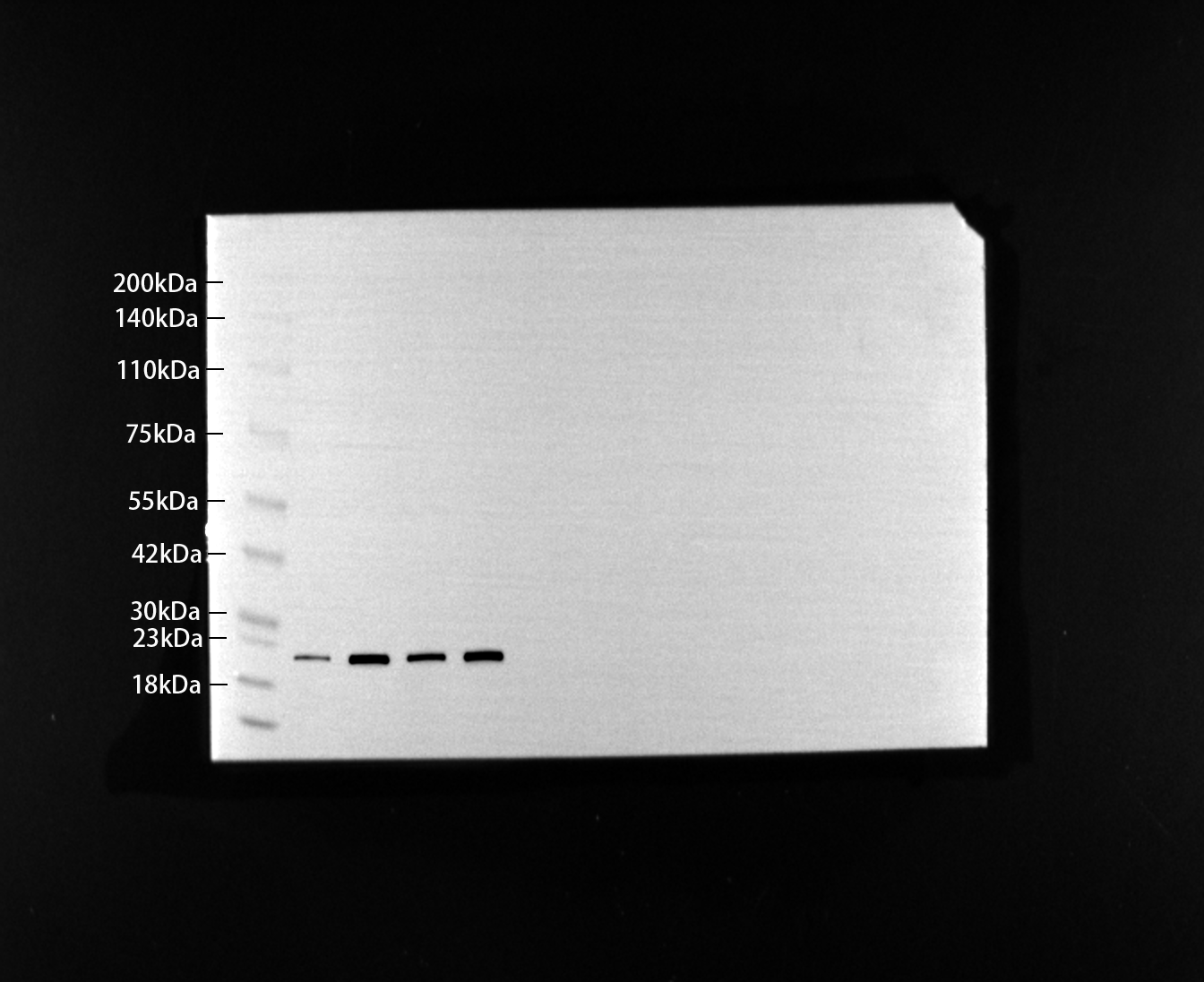

Supplement: Supplementary file 1 — Supplementary Material 1. [file 41065_2026_674_MOESM1_ESM.zip › Original image for western blot -marker/Original image Figure 6F/6F-Bax.tif]

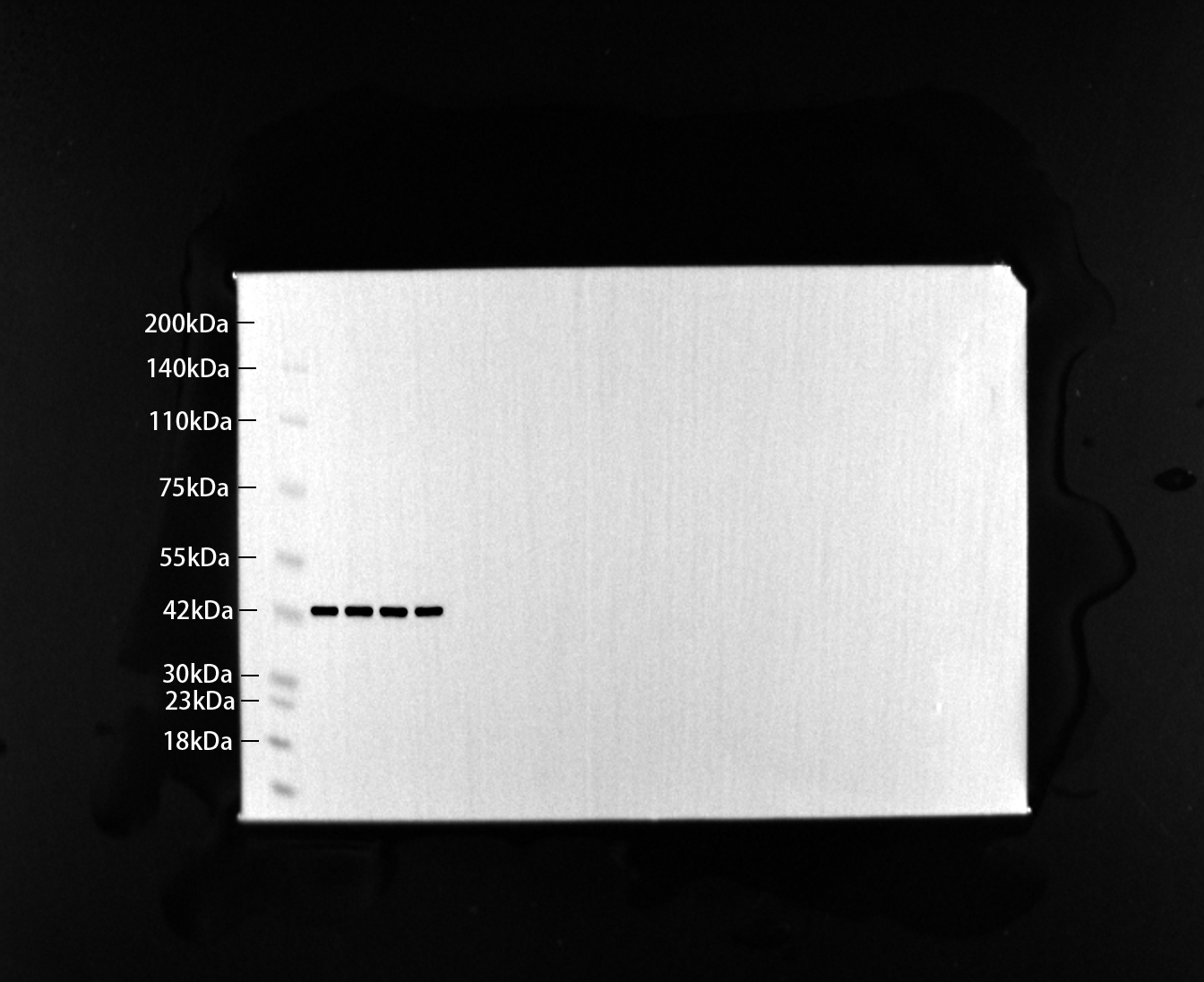

Supplement: Supplementary file 1 — Supplementary Material 1. [file 41065_2026_674_MOESM1_ESM.zip › Original image for western blot -marker/Original image Figure 5N/5N-β-actin.tif]

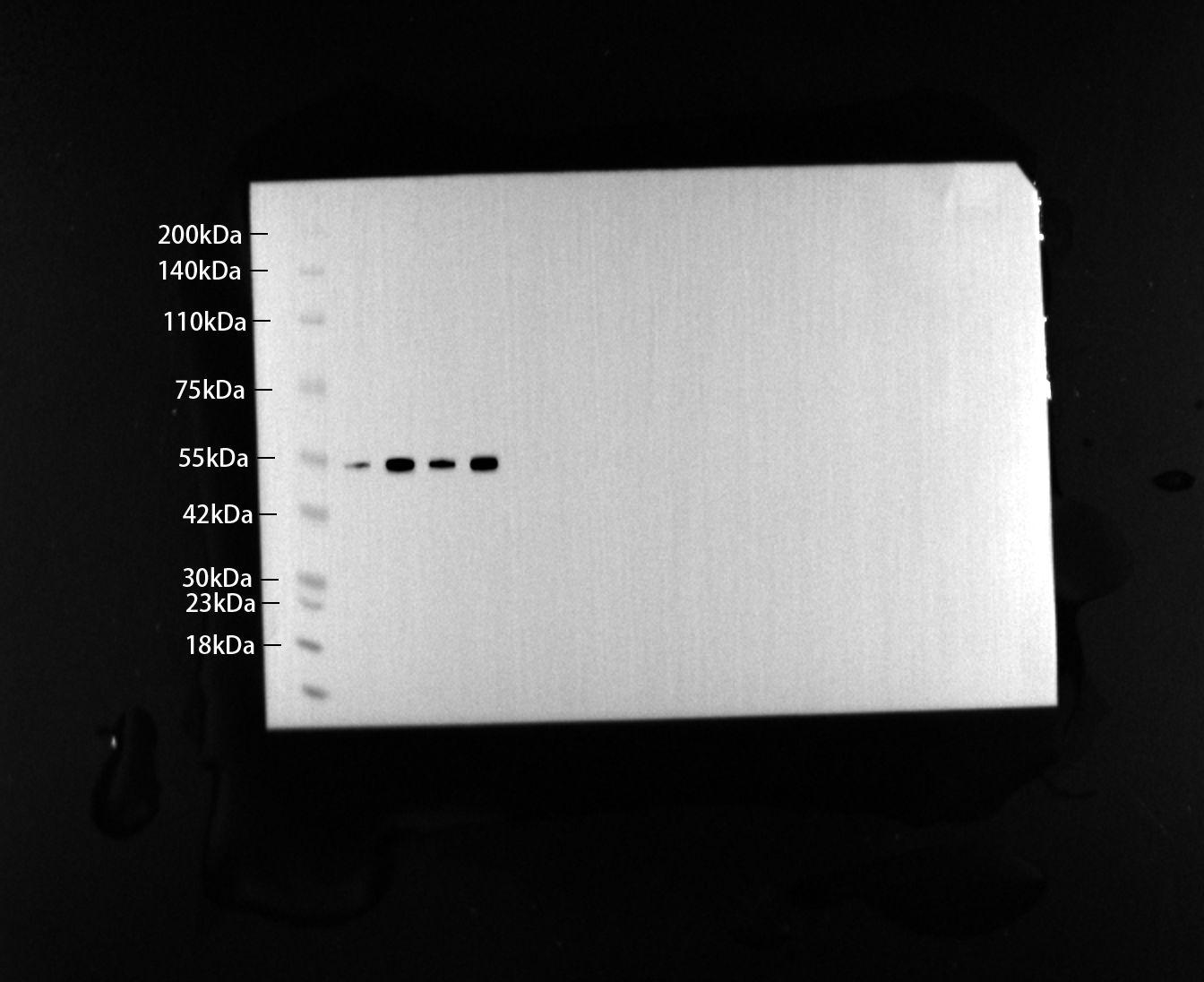

Supplement: Supplementary file 1 — Supplementary Material 1. [file 41065_2026_674_MOESM1_ESM.zip › Original image for western blot -marker/Original image Figure 5N/5N-MMP13.tif]

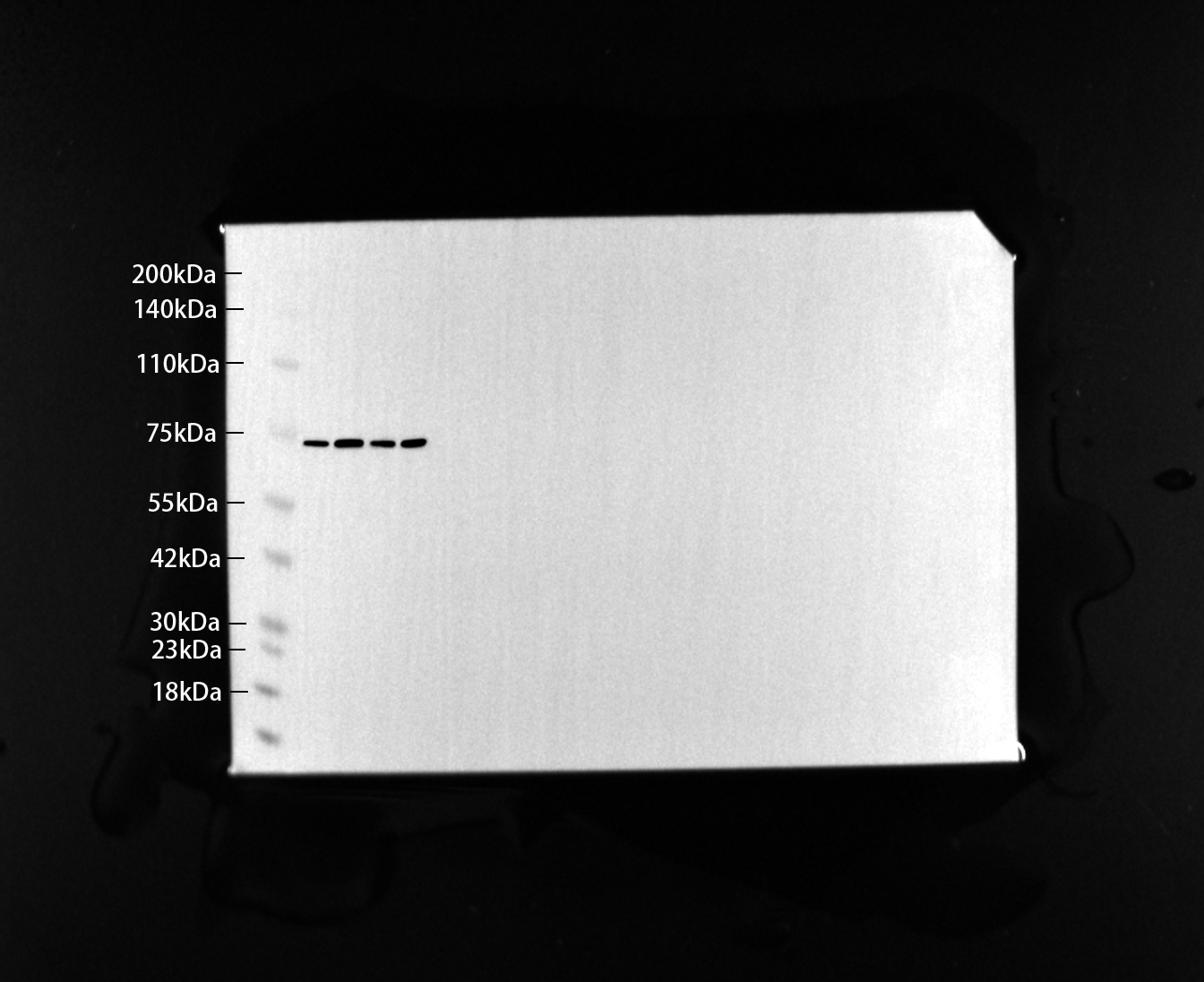

Supplement: Supplementary file 1 — Supplementary Material 1. [file 41065_2026_674_MOESM1_ESM.zip › Original image for western blot -marker/Original image Figure 5N/5N-ADAMTS5.tif]

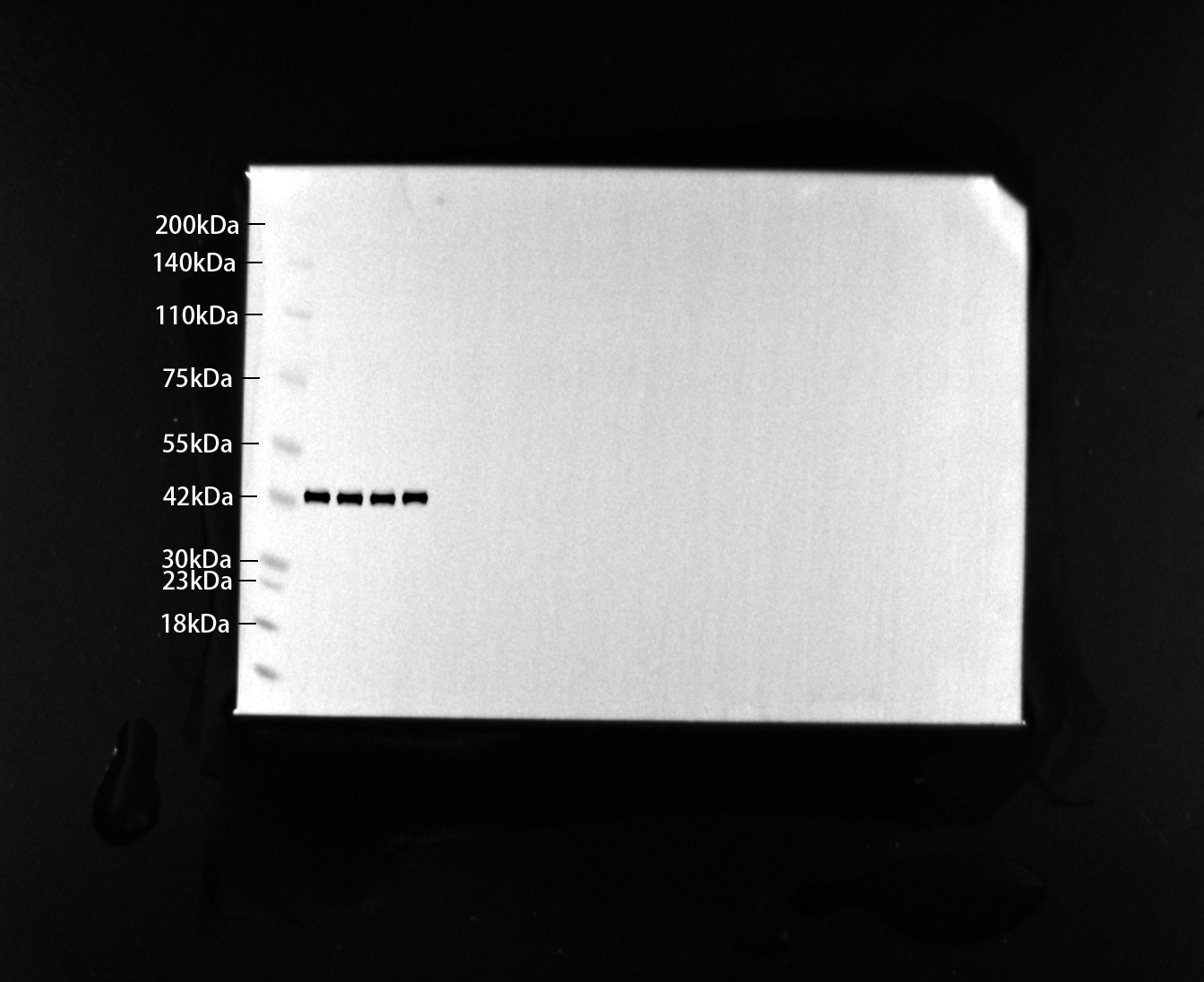

Supplement: Supplementary file 1 — Supplementary Material 1. [file 41065_2026_674_MOESM1_ESM.zip › Original image for western blot -marker/Original image Figure 5L/5L-β-actin.tif]

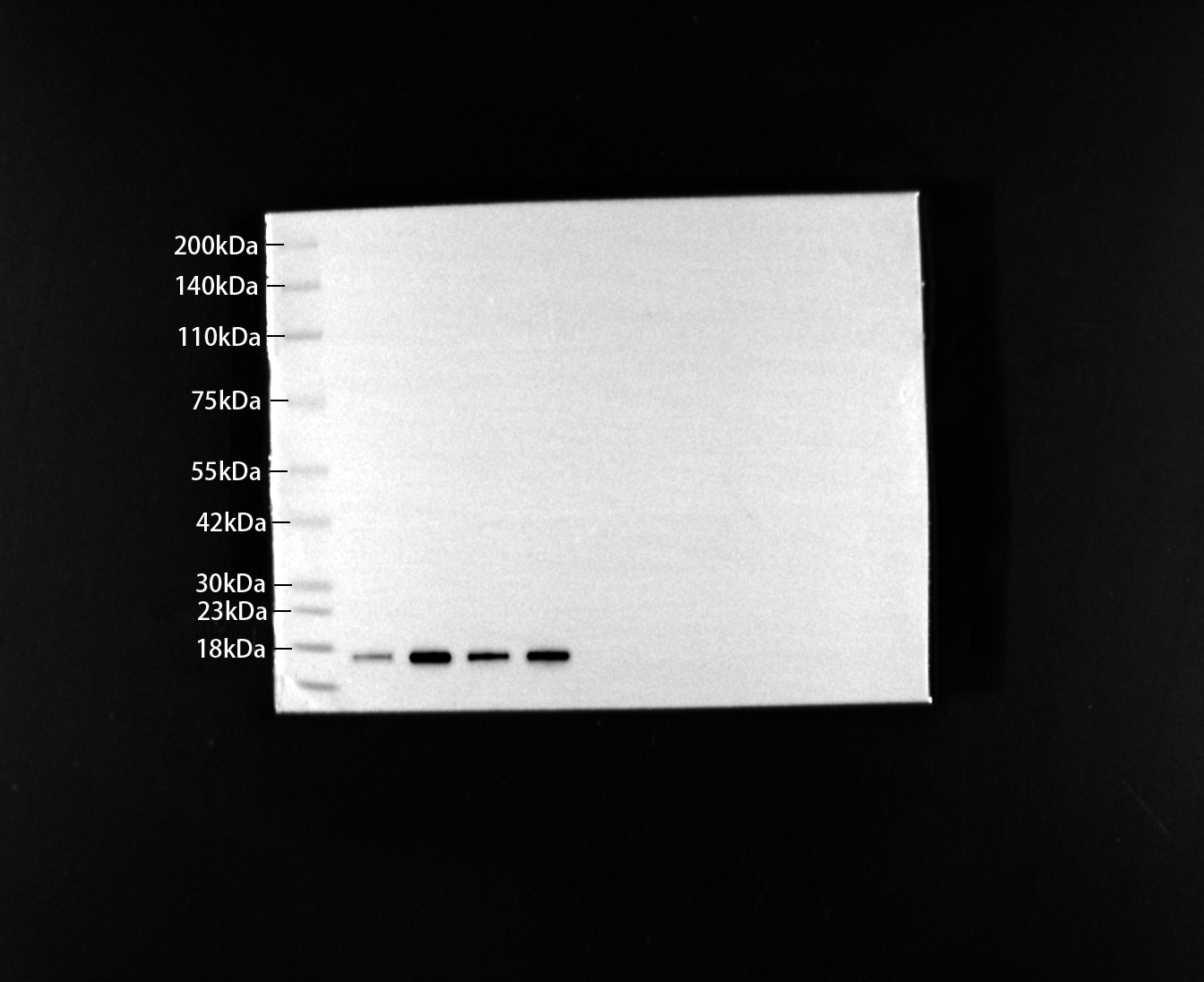

Supplement: Supplementary file 1 — Supplementary Material 1. [file 41065_2026_674_MOESM1_ESM.zip › Original image for western blot -marker/Original image Figure 5L/5L-Cleaved caspase-3.tif]

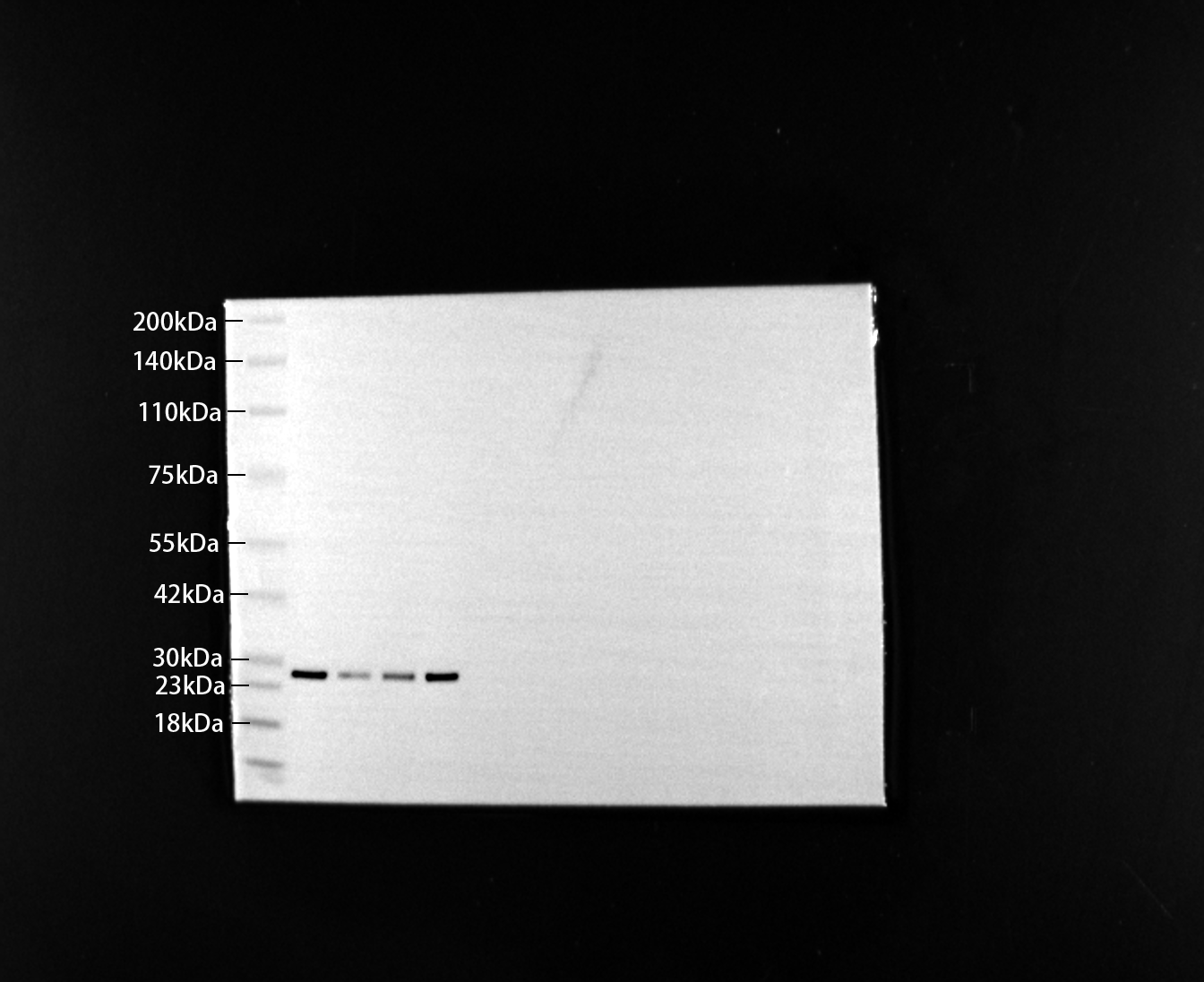

Supplement: Supplementary file 1 — Supplementary Material 1. [file 41065_2026_674_MOESM1_ESM.zip › Original image for western blot -marker/Original image Figure 5L/5L-BcI2.tif]

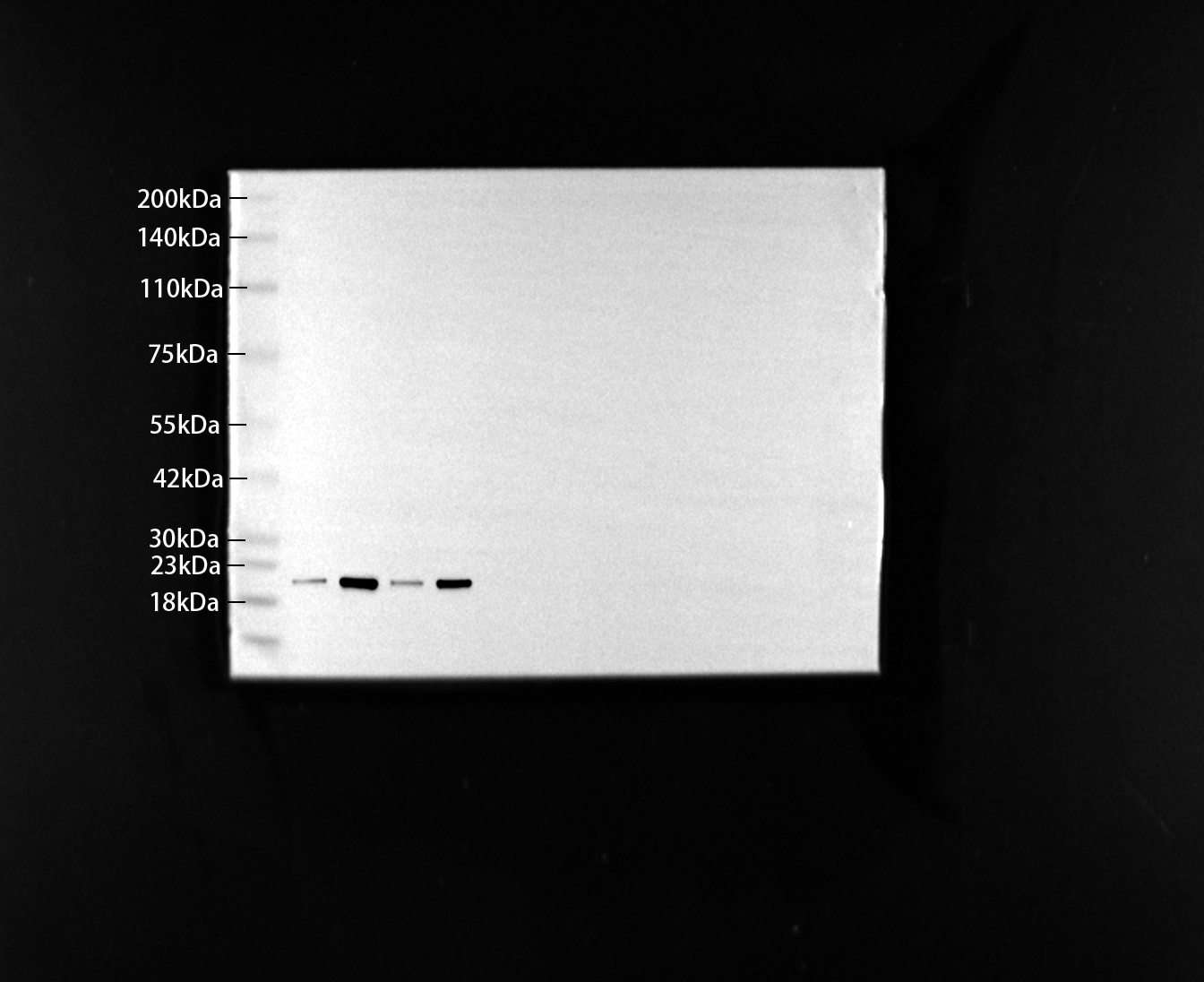

Supplement: Supplementary file 1 — Supplementary Material 1. [file 41065_2026_674_MOESM1_ESM.zip › Original image for western blot -marker/Original image Figure 5L/5L-Bax.tif]

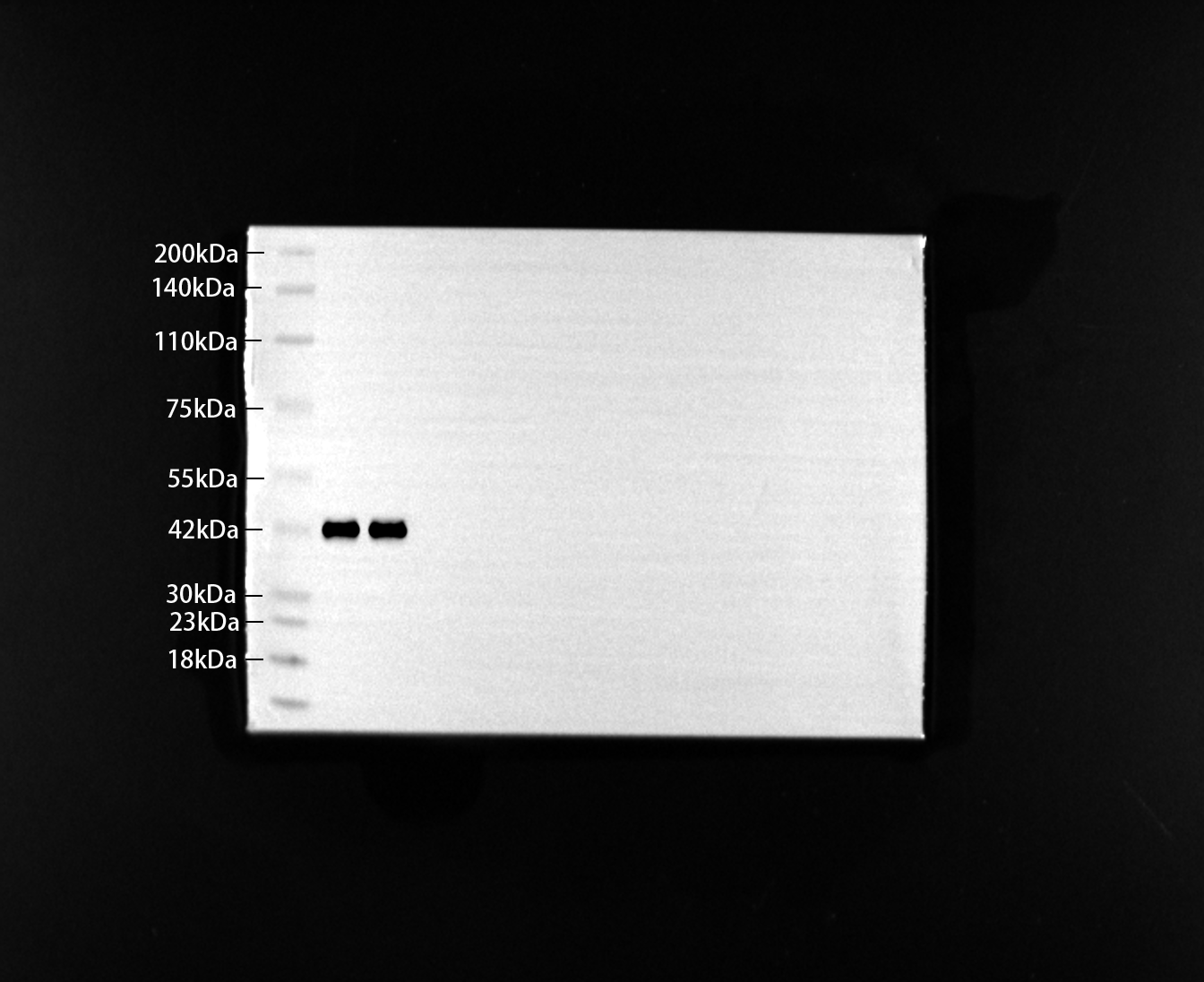

Supplement: Supplementary file 1 — Supplementary Material 1. [file 41065_2026_674_MOESM1_ESM.zip › Original image for western blot -marker/Original image Figure 5I/5I-β-actin.tif]

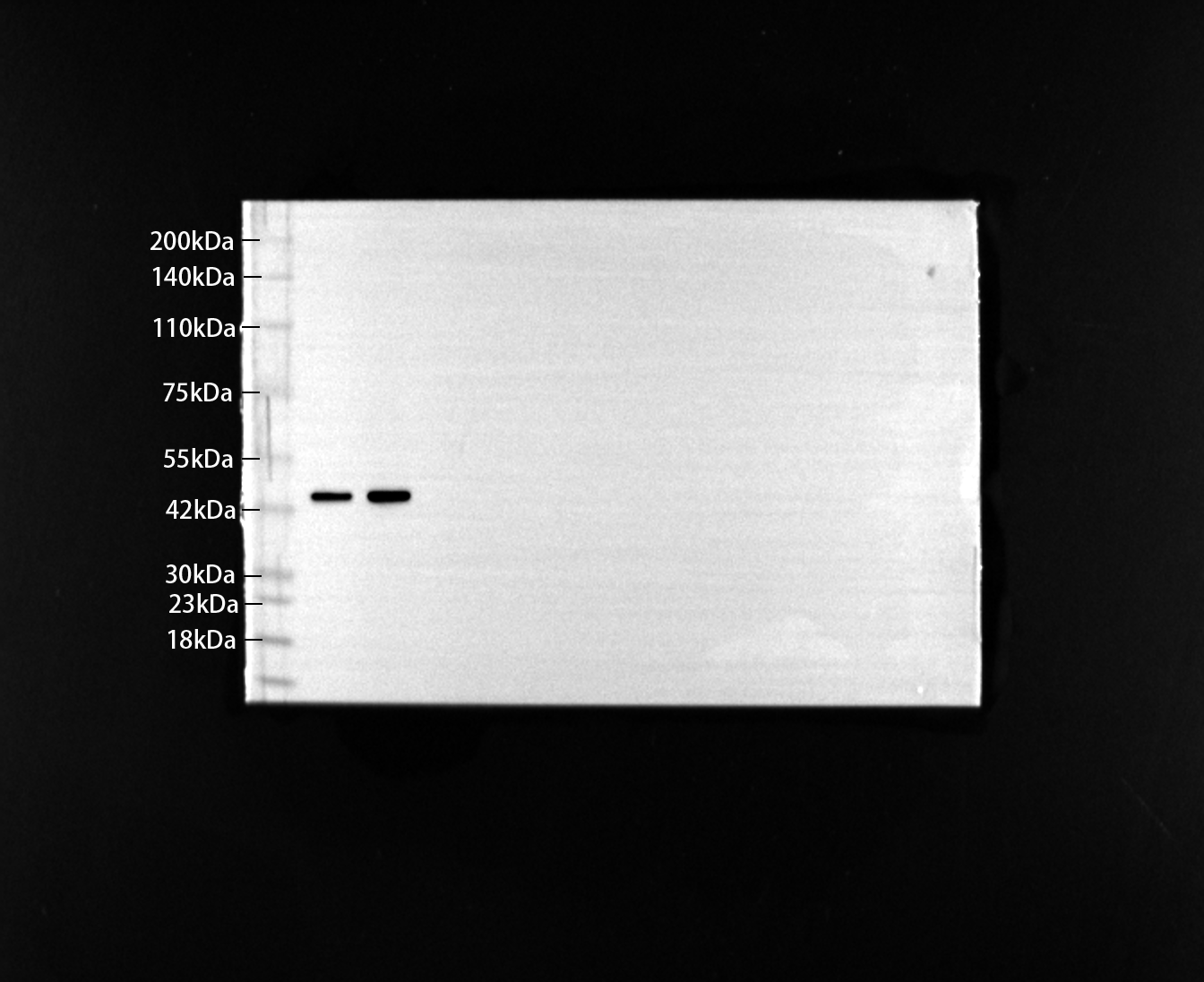

Supplement: Supplementary file 1 — Supplementary Material 1. [file 41065_2026_674_MOESM1_ESM.zip › Original image for western blot -marker/Original image Figure 5I/5I-EIF4A1.tif]

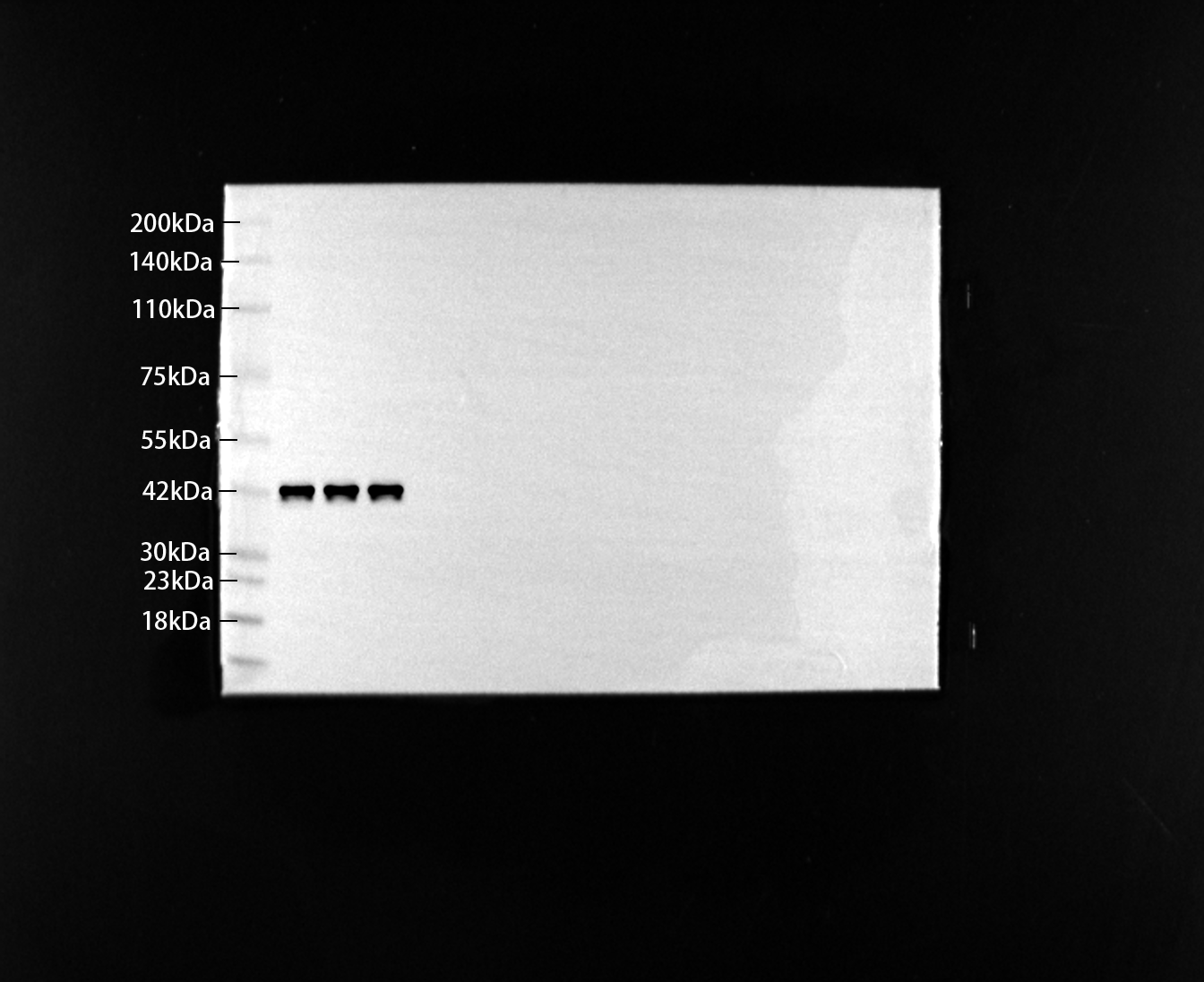

Supplement: Supplementary file 1 — Supplementary Material 1. [file 41065_2026_674_MOESM1_ESM.zip › Original image for western blot -marker/Original image Figure 5E/5E-β-actin.tif]

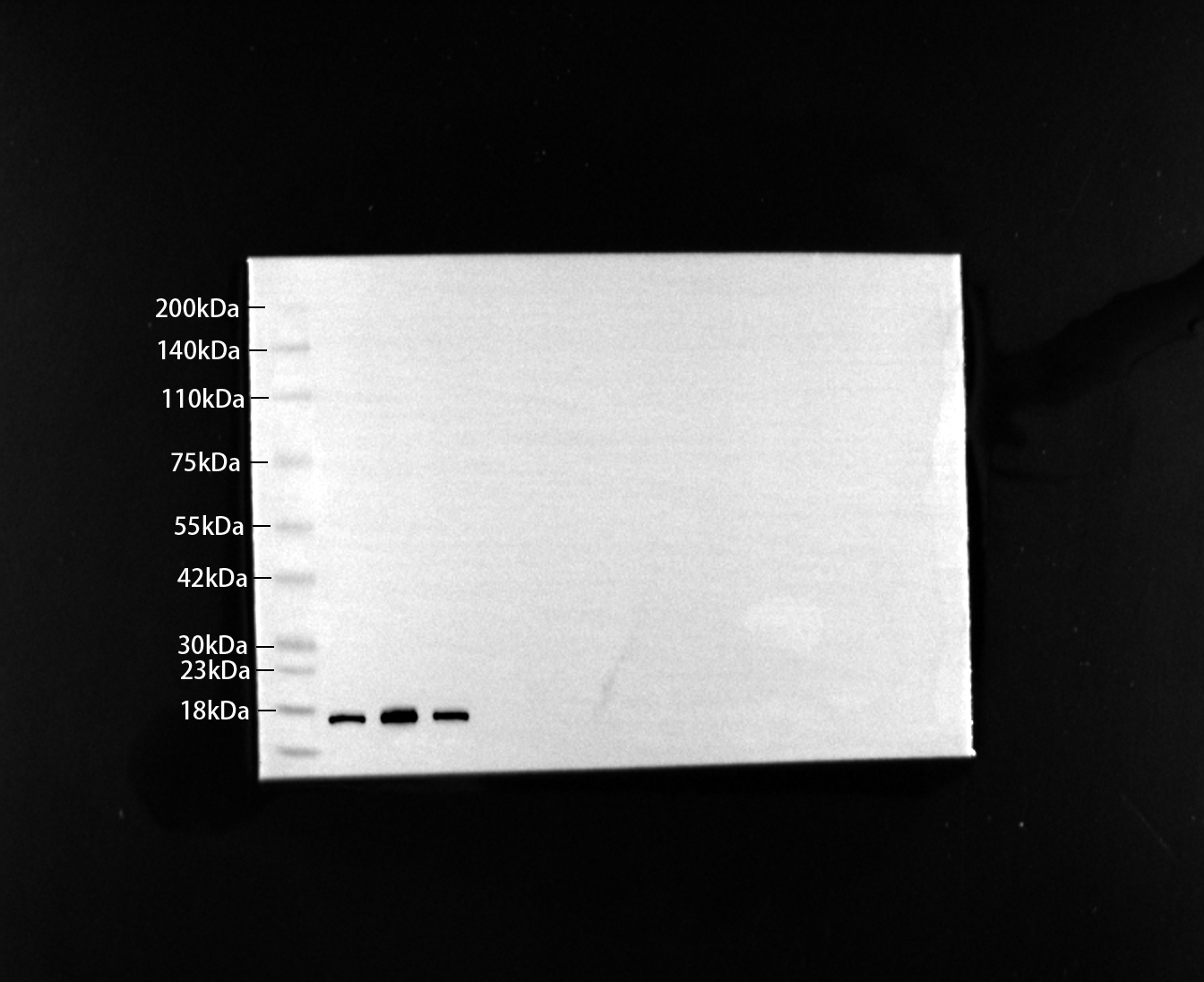

Supplement: Supplementary file 1 — Supplementary Material 1. [file 41065_2026_674_MOESM1_ESM.zip › Original image for western blot -marker/Original image Figure 5E/5E-Cleaved caspase-3.tif]

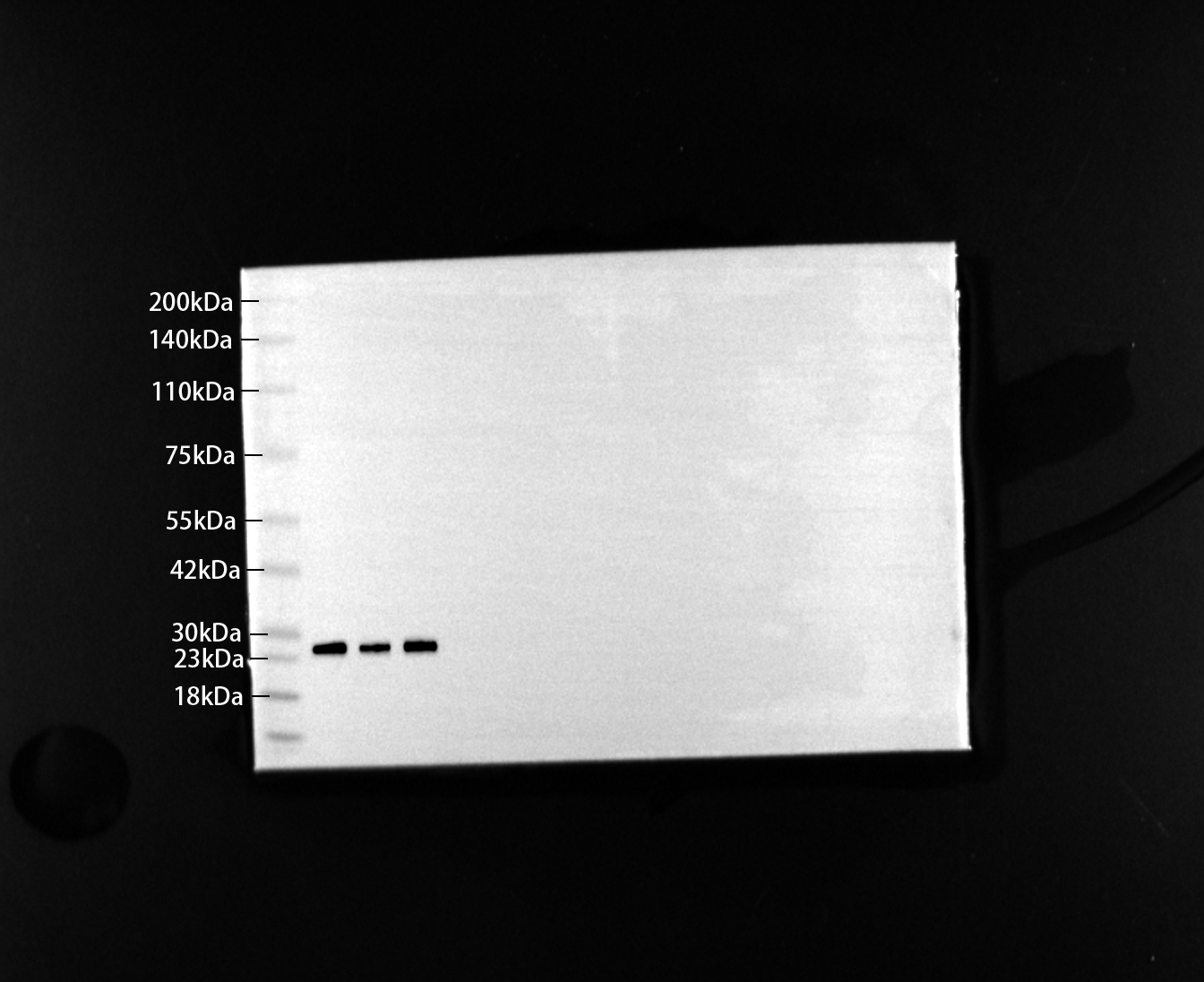

Supplement: Supplementary file 1 — Supplementary Material 1. [file 41065_2026_674_MOESM1_ESM.zip › Original image for western blot -marker/Original image Figure 5E/5E-BcI2.tif]

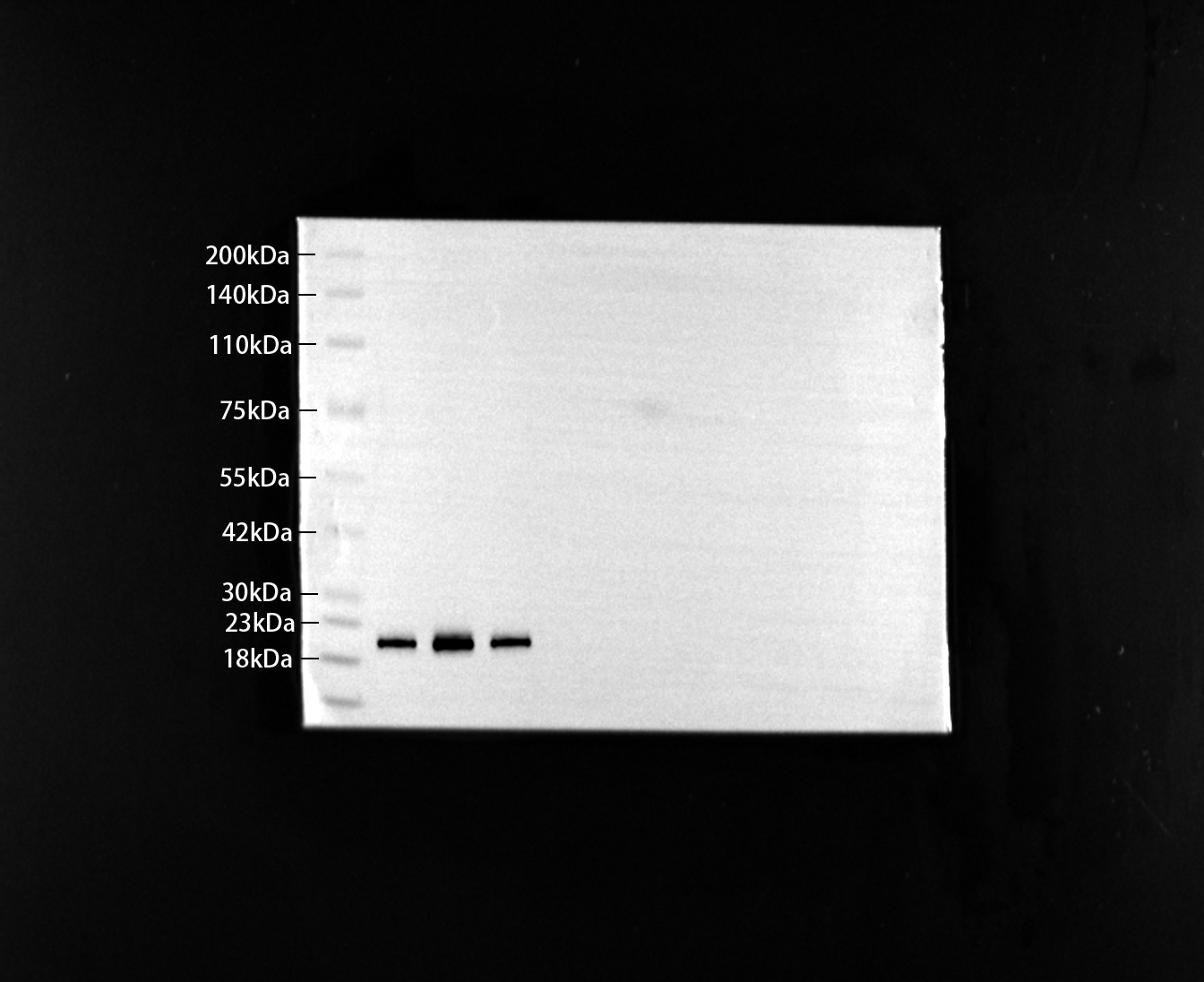

Supplement: Supplementary file 1 — Supplementary Material 1. [file 41065_2026_674_MOESM1_ESM.zip › Original image for western blot -marker/Original image Figure 5E/5E-Bax.tif]

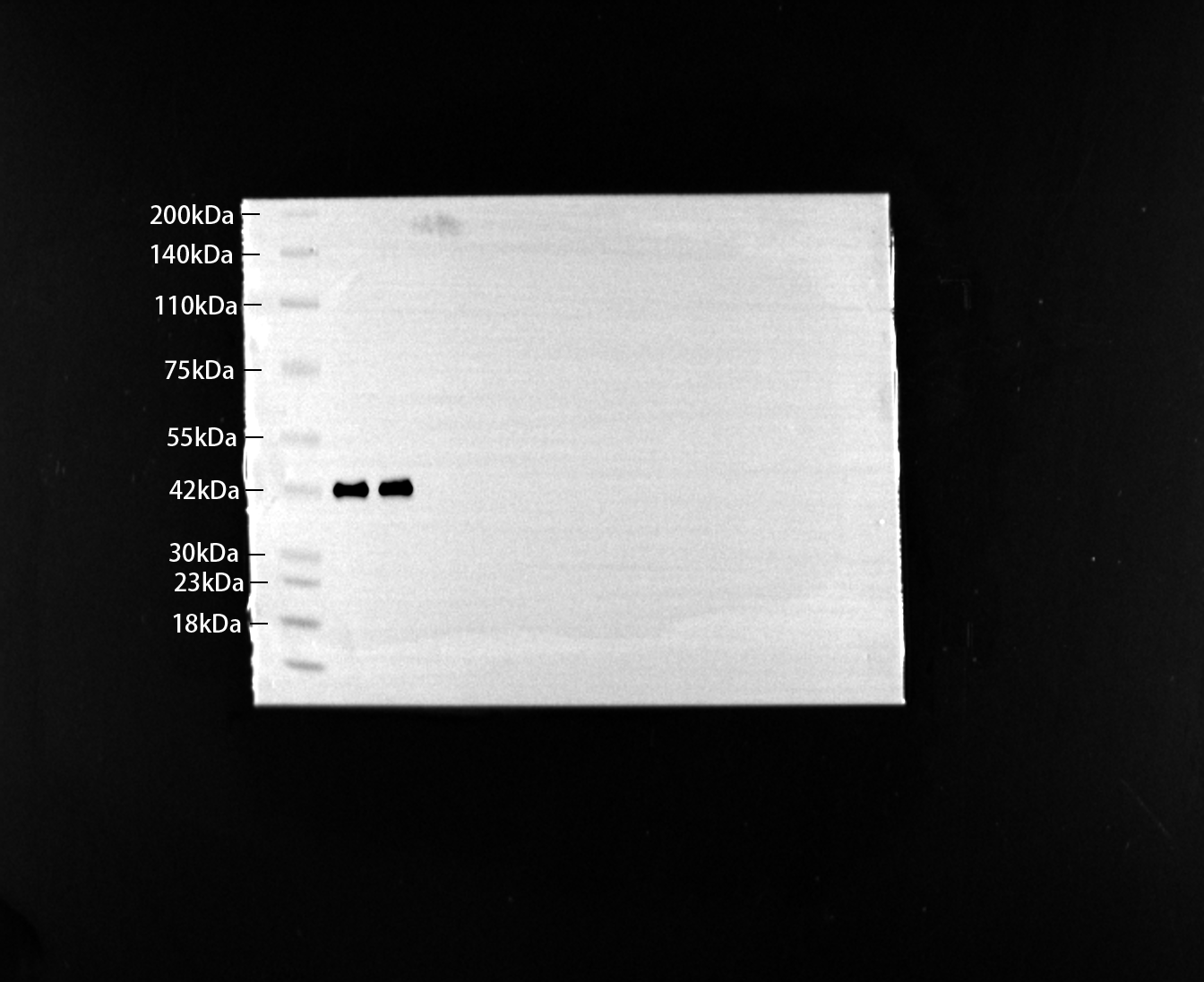

Supplement: Supplementary file 1 — Supplementary Material 1. [file 41065_2026_674_MOESM1_ESM.zip › Original image for western blot -marker/Original image Figure 5B/5B-β-actin.tif]

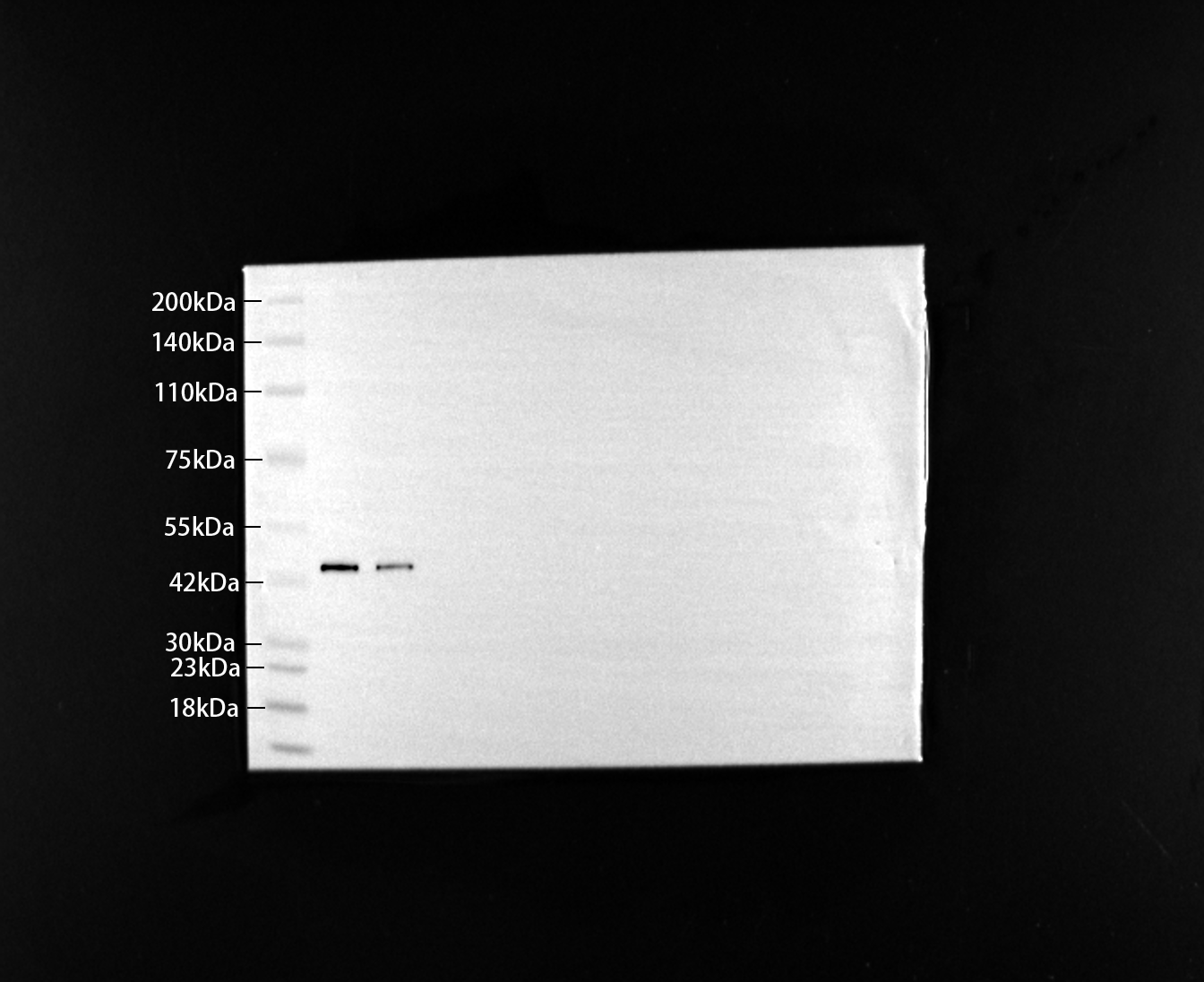

Supplement: Supplementary file 1 — Supplementary Material 1. [file 41065_2026_674_MOESM1_ESM.zip › Original image for western blot -marker/Original image Figure 5B/5B-EIF4A1.tif]

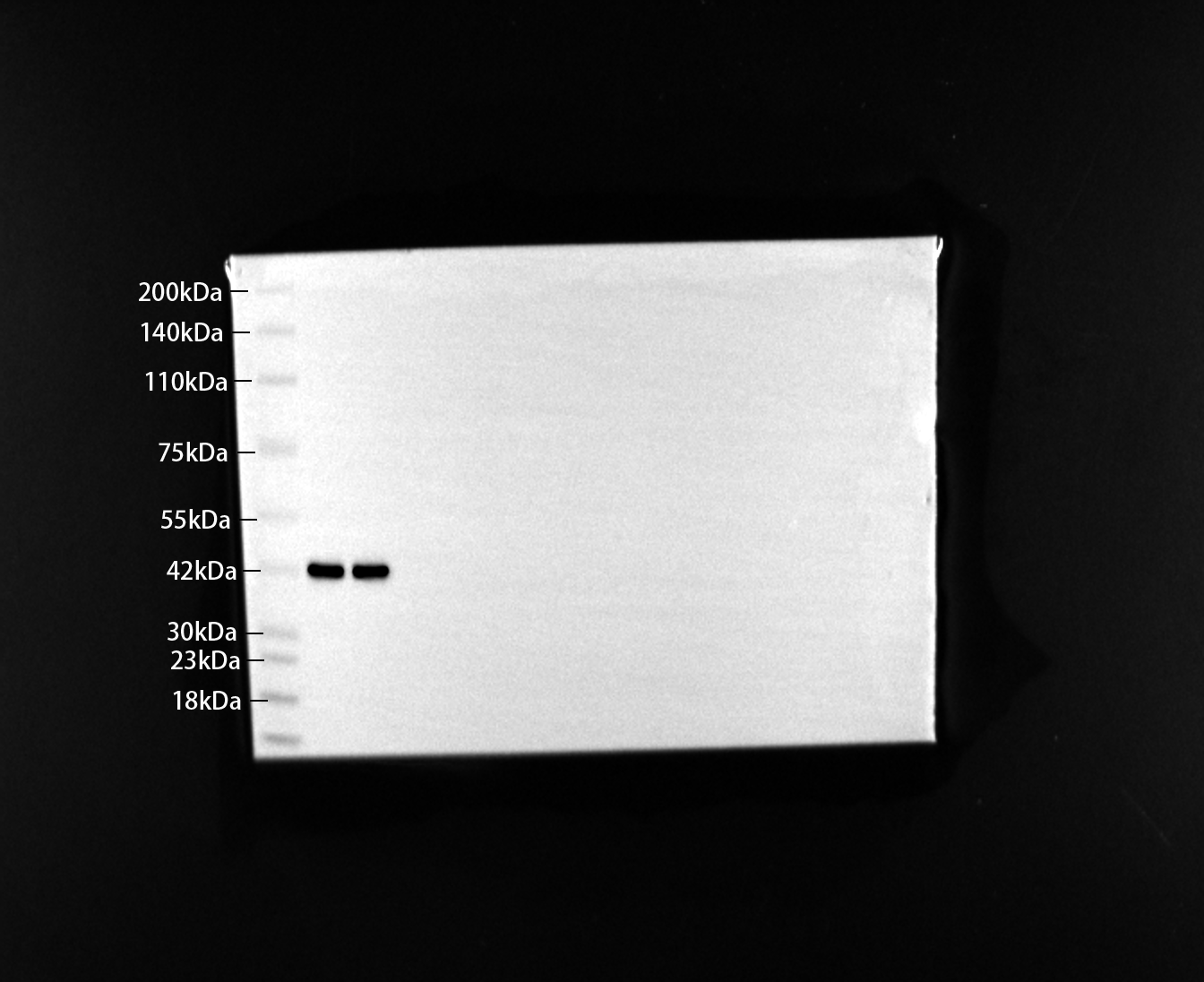

Supplement: Supplementary file 1 — Supplementary Material 1. [file 41065_2026_674_MOESM1_ESM.zip › Original image for western blot -marker/Original image Figure 4D/4D-β-actin.tif]

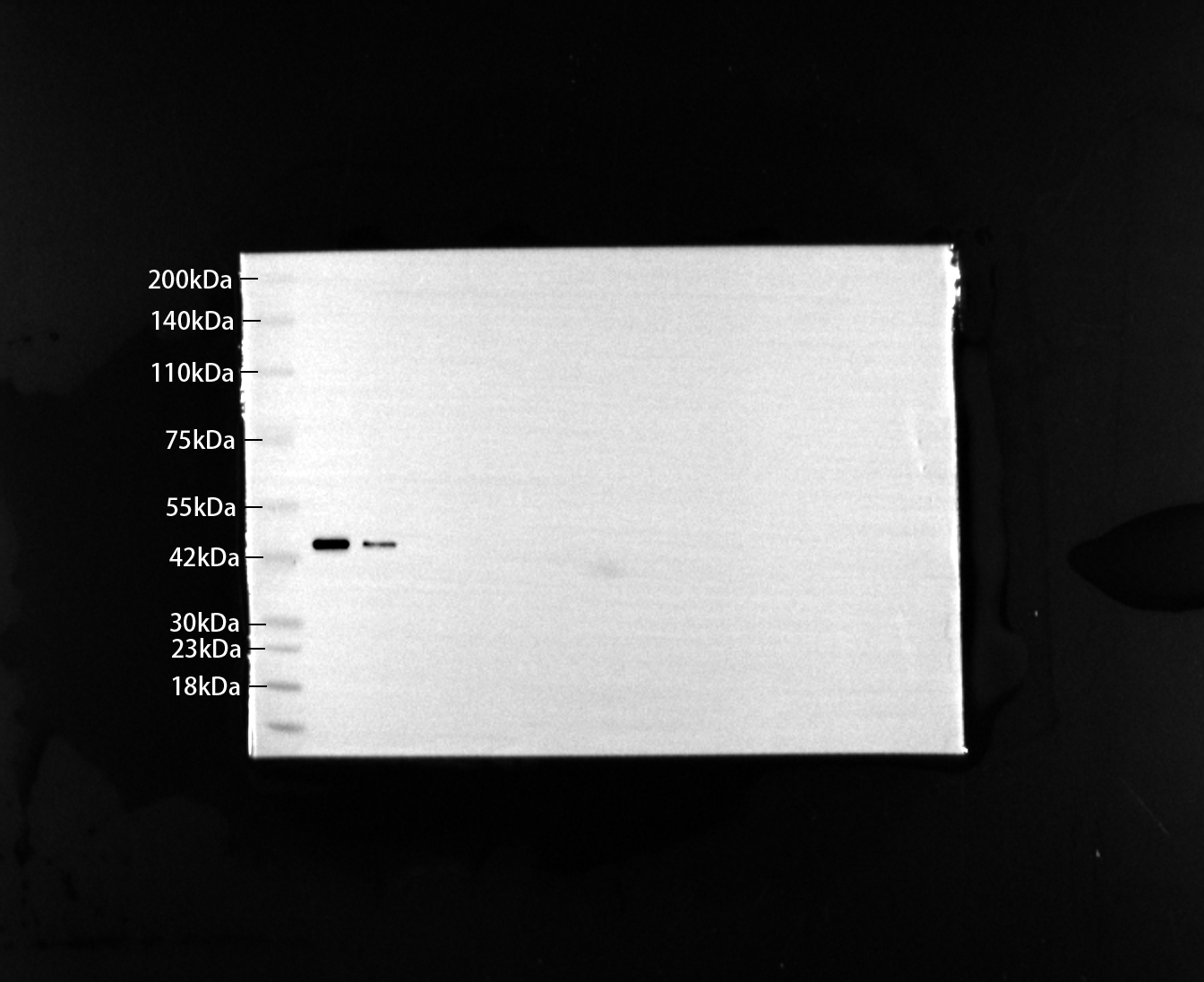

Supplement: Supplementary file 1 — Supplementary Material 1. [file 41065_2026_674_MOESM1_ESM.zip › Original image for western blot -marker/Original image Figure 4D/4D-EIF4A1.tif]

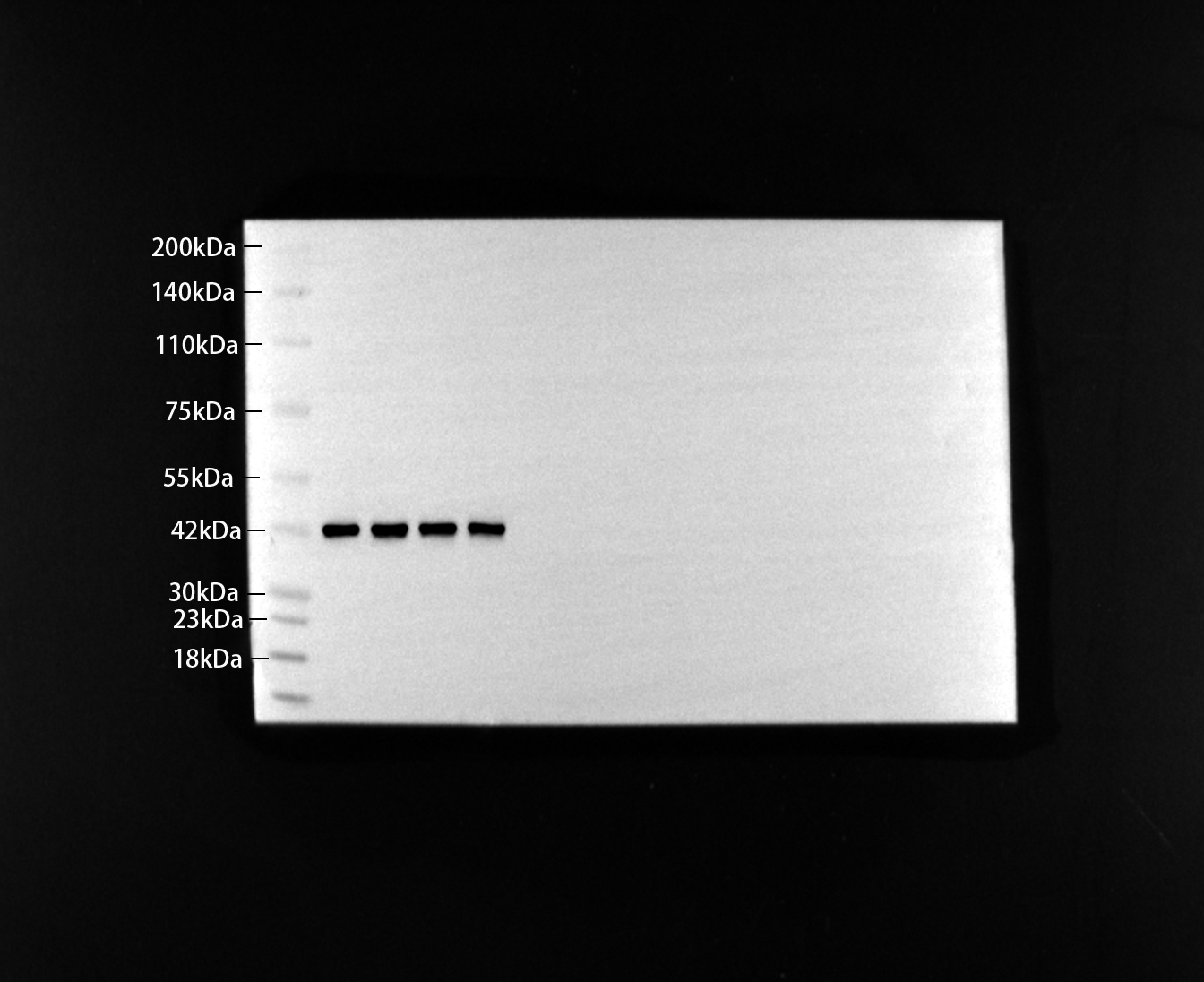

Supplement: Supplementary file 1 — Supplementary Material 1. [file 41065_2026_674_MOESM1_ESM.zip › Original image for western blot -marker/Original image Figure 3F/3F-β-actin.tif]

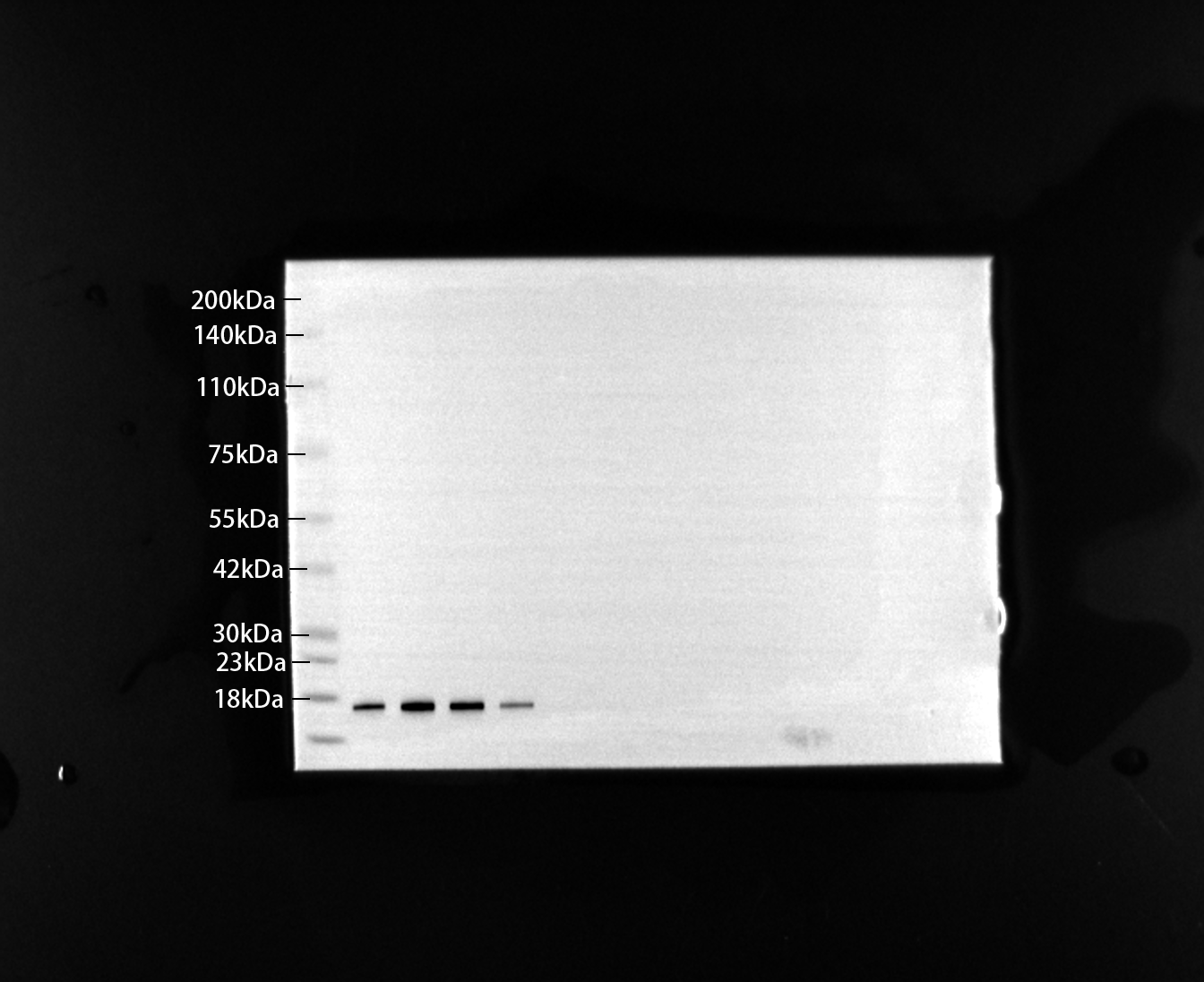

Supplement: Supplementary file 1 — Supplementary Material 1. [file 41065_2026_674_MOESM1_ESM.zip › Original image for western blot -marker/Original image Figure 3F/3F-Cleaved caspase-3.tif]

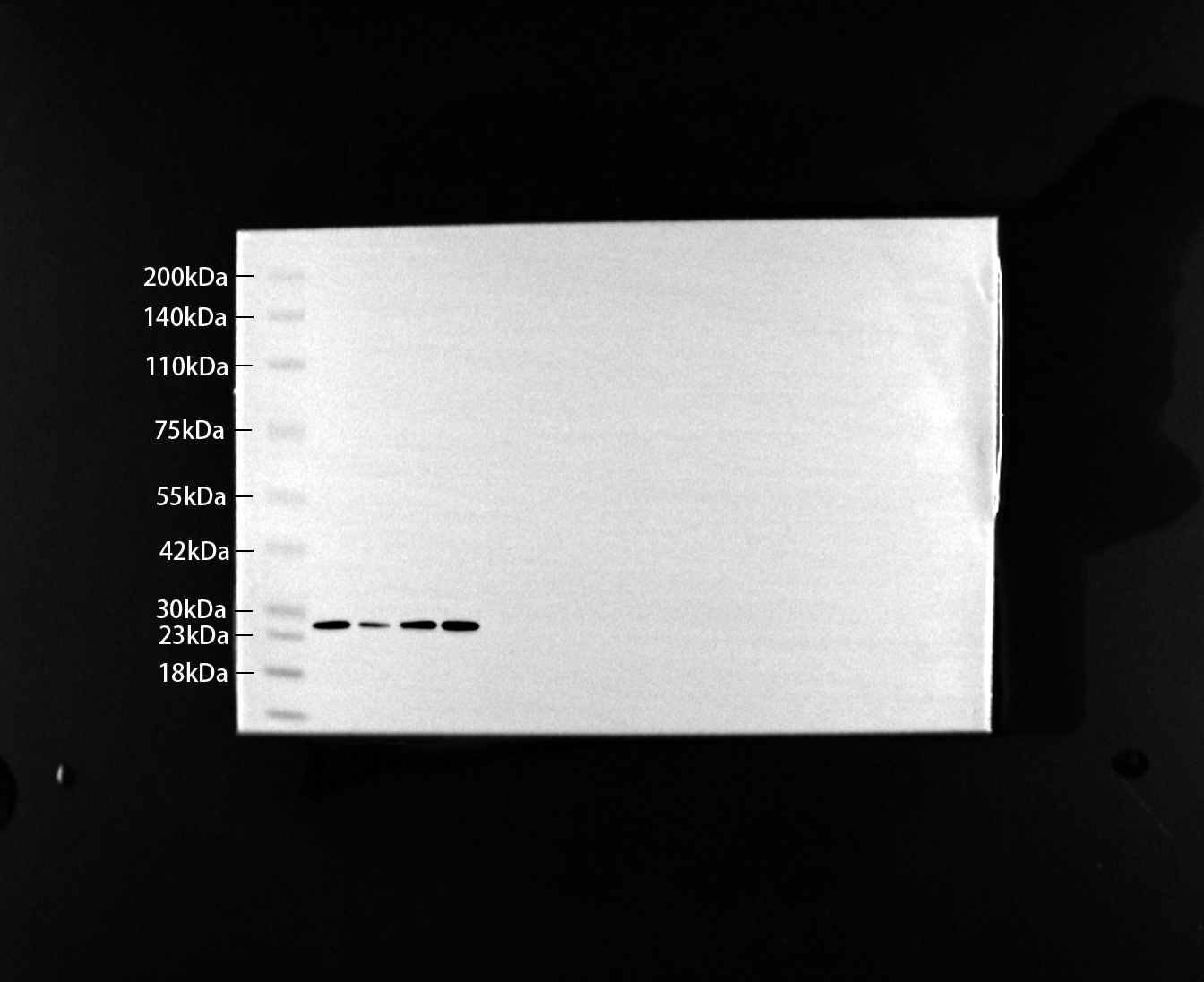

Supplement: Supplementary file 1 — Supplementary Material 1. [file 41065_2026_674_MOESM1_ESM.zip › Original image for western blot -marker/Original image Figure 3F/3F-BcI2.tif]

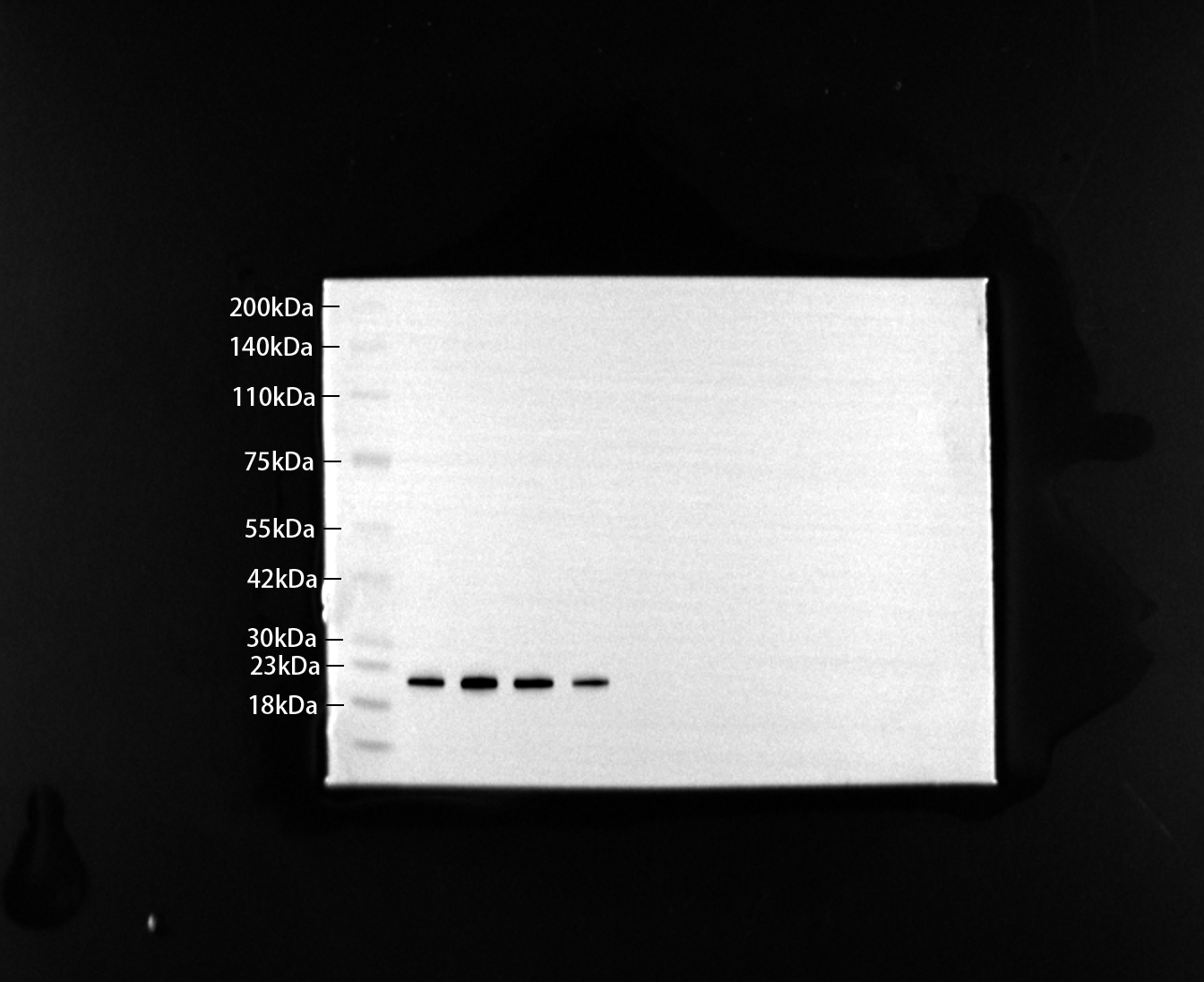

Supplement: Supplementary file 1 — Supplementary Material 1. [file 41065_2026_674_MOESM1_ESM.zip › Original image for western blot -marker/Original image Figure 3F/3F-Bax.tif]

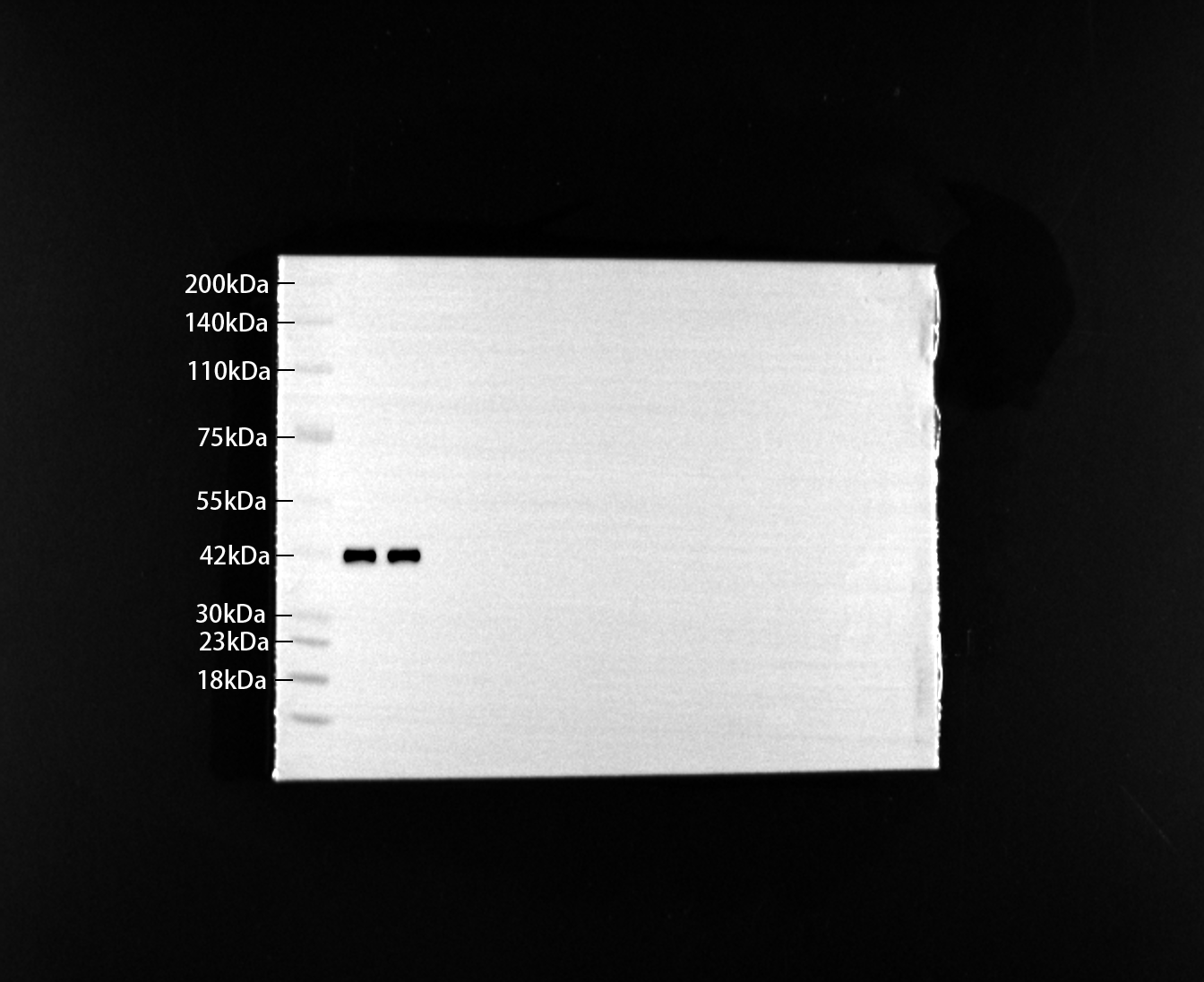

Supplement: Supplementary file 1 — Supplementary Material 1. [file 41065_2026_674_MOESM1_ESM.zip › Original image for western blot -marker/Original image Figure 3B/3B-β-actin.tif]

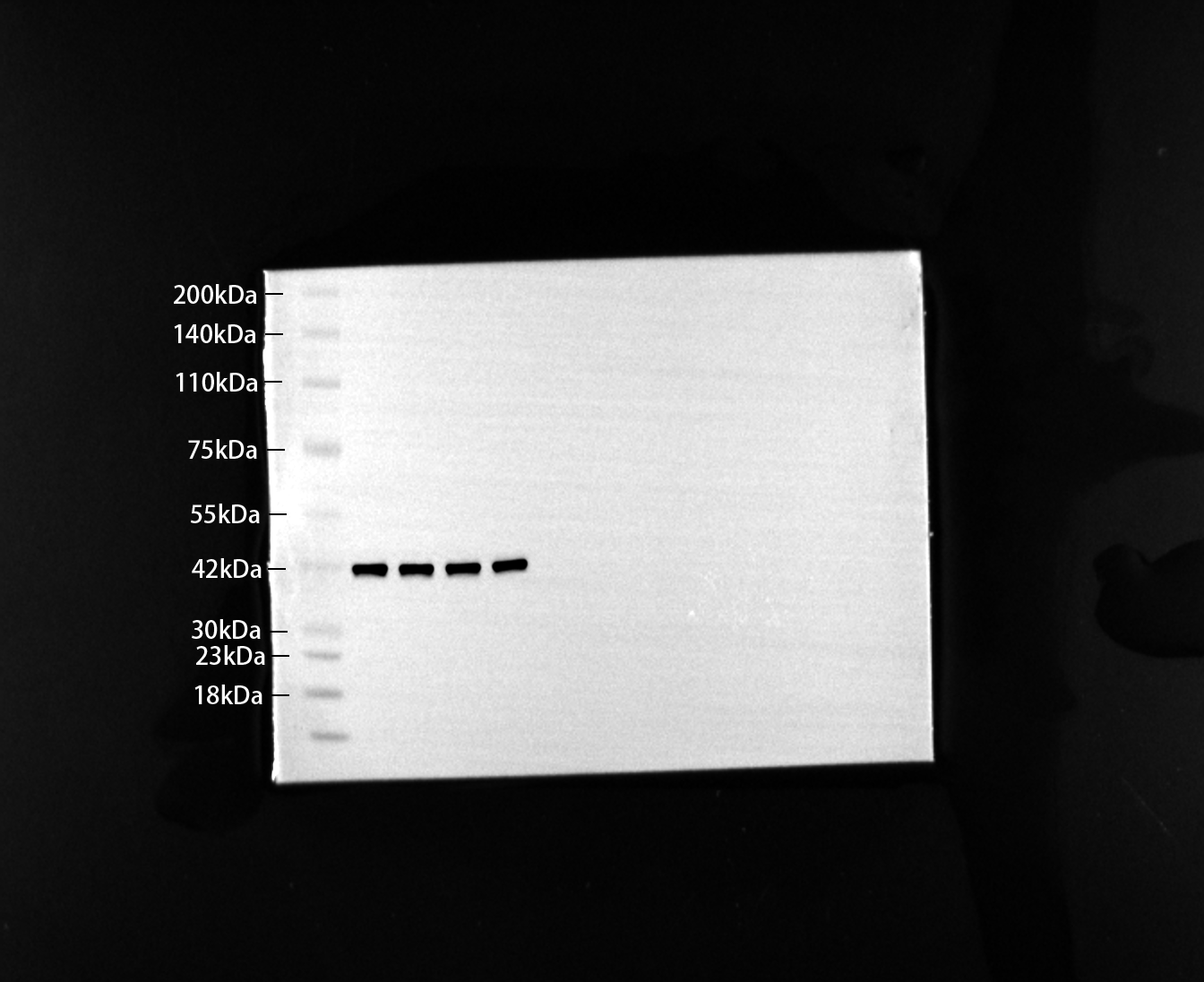

Supplement: Supplementary file 1 — Supplementary Material 1. [file 41065_2026_674_MOESM1_ESM.zip › Original image for western blot -marker/Original image Figure 2J/2J-β-actin.tif]

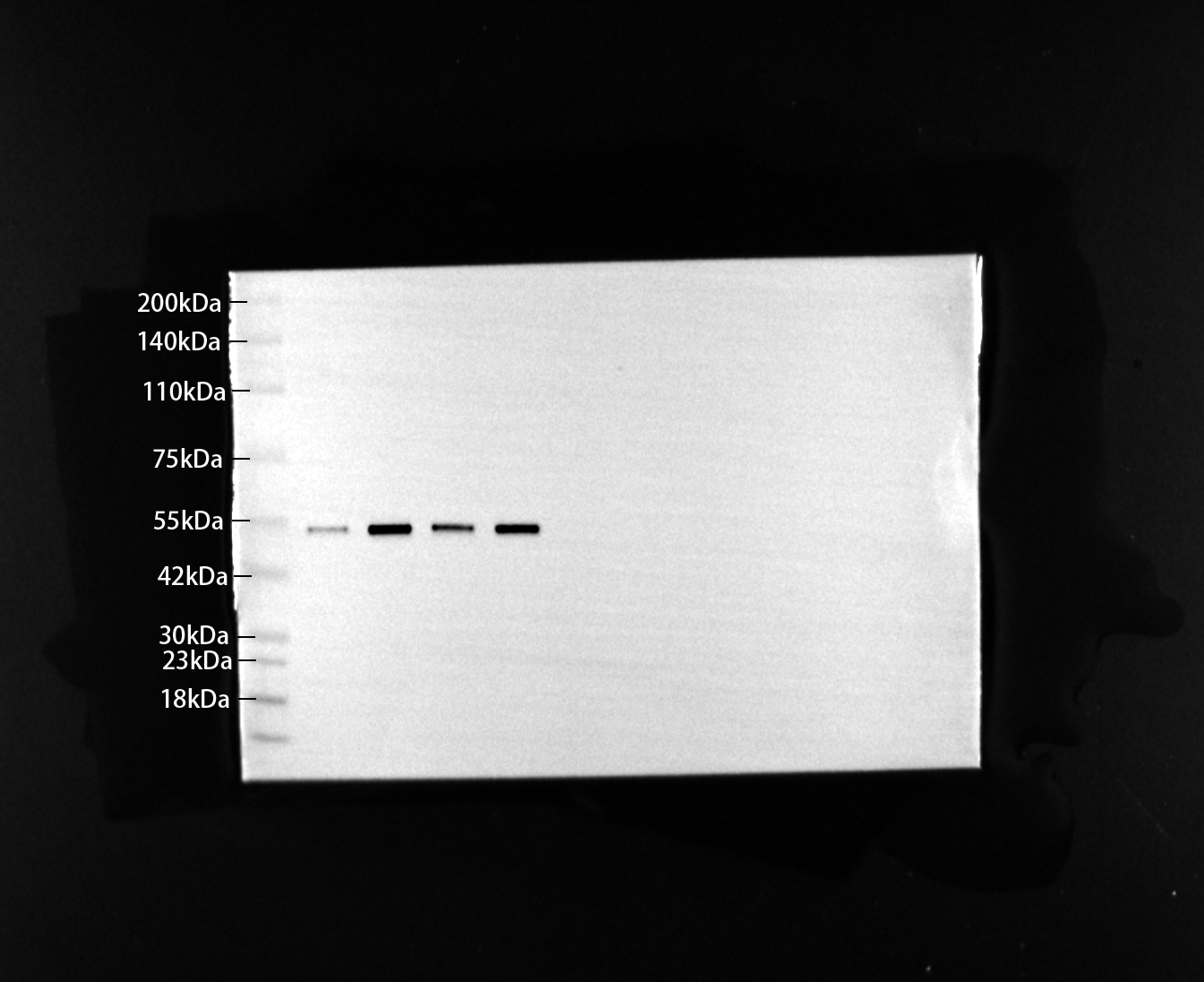

Supplement: Supplementary file 1 — Supplementary Material 1. [file 41065_2026_674_MOESM1_ESM.zip › Original image for western blot -marker/Original image Figure 2J/2J-MMP13.tif]

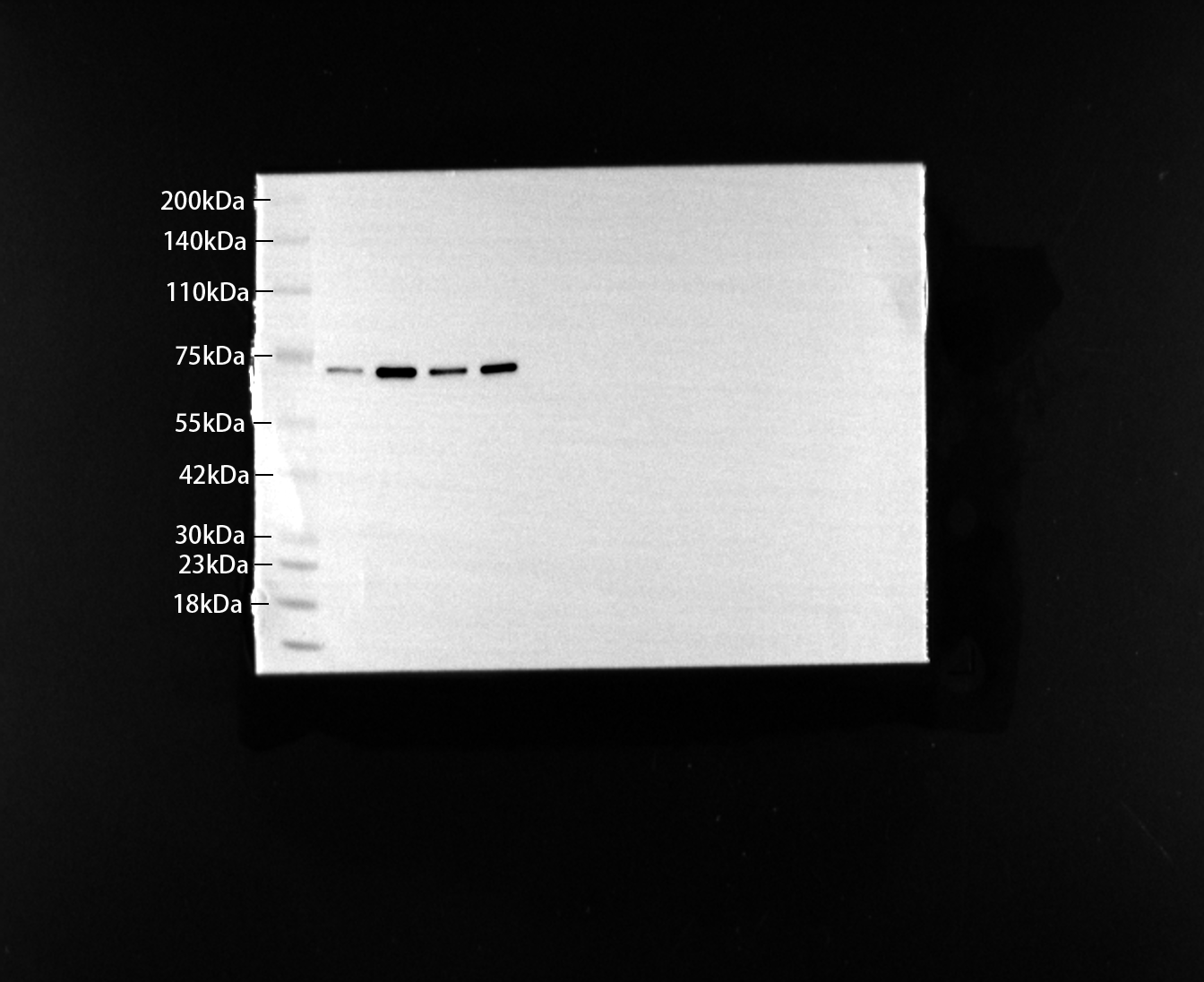

Supplement: Supplementary file 1 — Supplementary Material 1. [file 41065_2026_674_MOESM1_ESM.zip › Original image for western blot -marker/Original image Figure 2J/2J-ADAMTS5.tif]

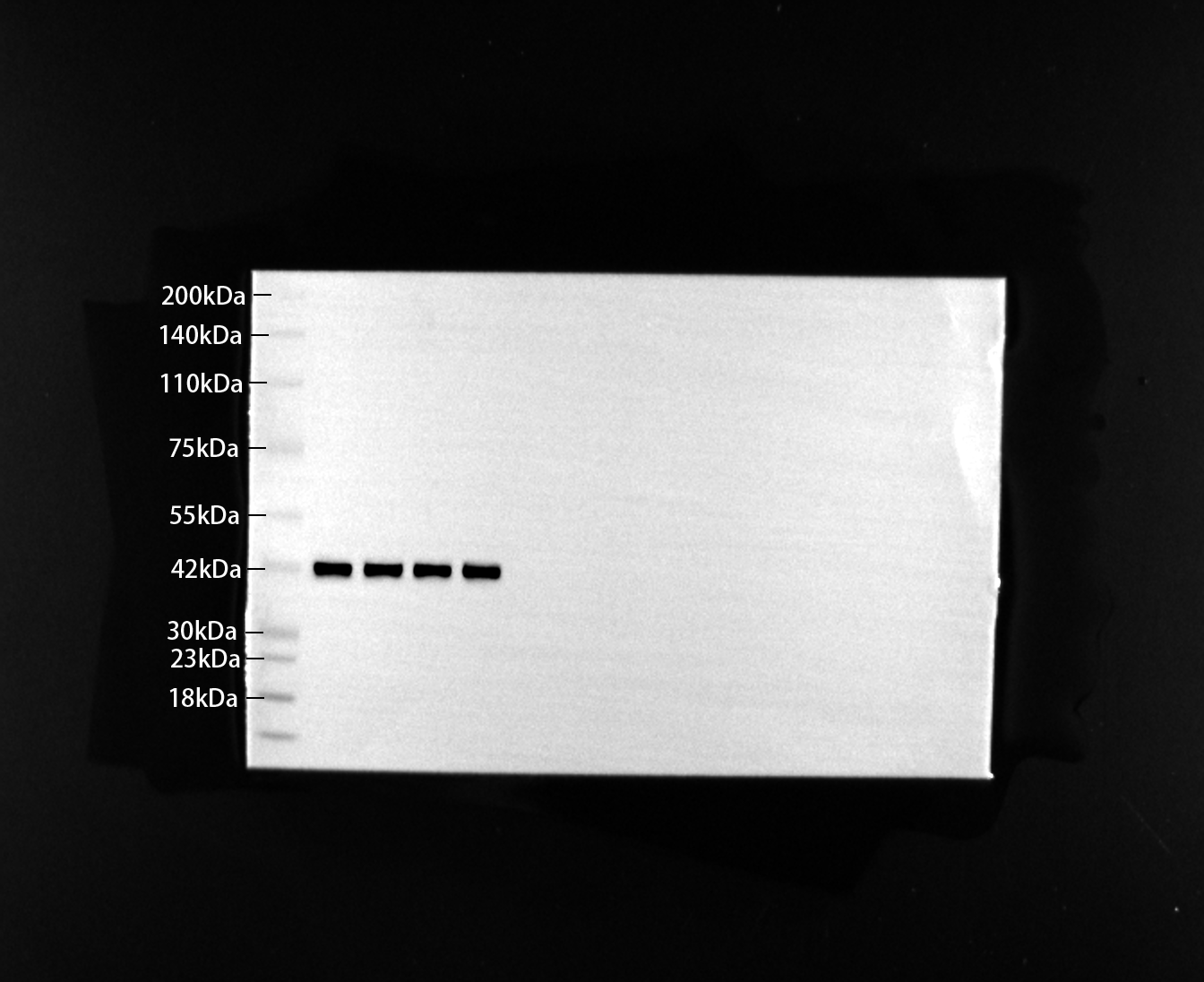

Supplement: Supplementary file 1 — Supplementary Material 1. [file 41065_2026_674_MOESM1_ESM.zip › Original image for western blot -marker/Original image Figure 2H/2H-β-actin.tif]

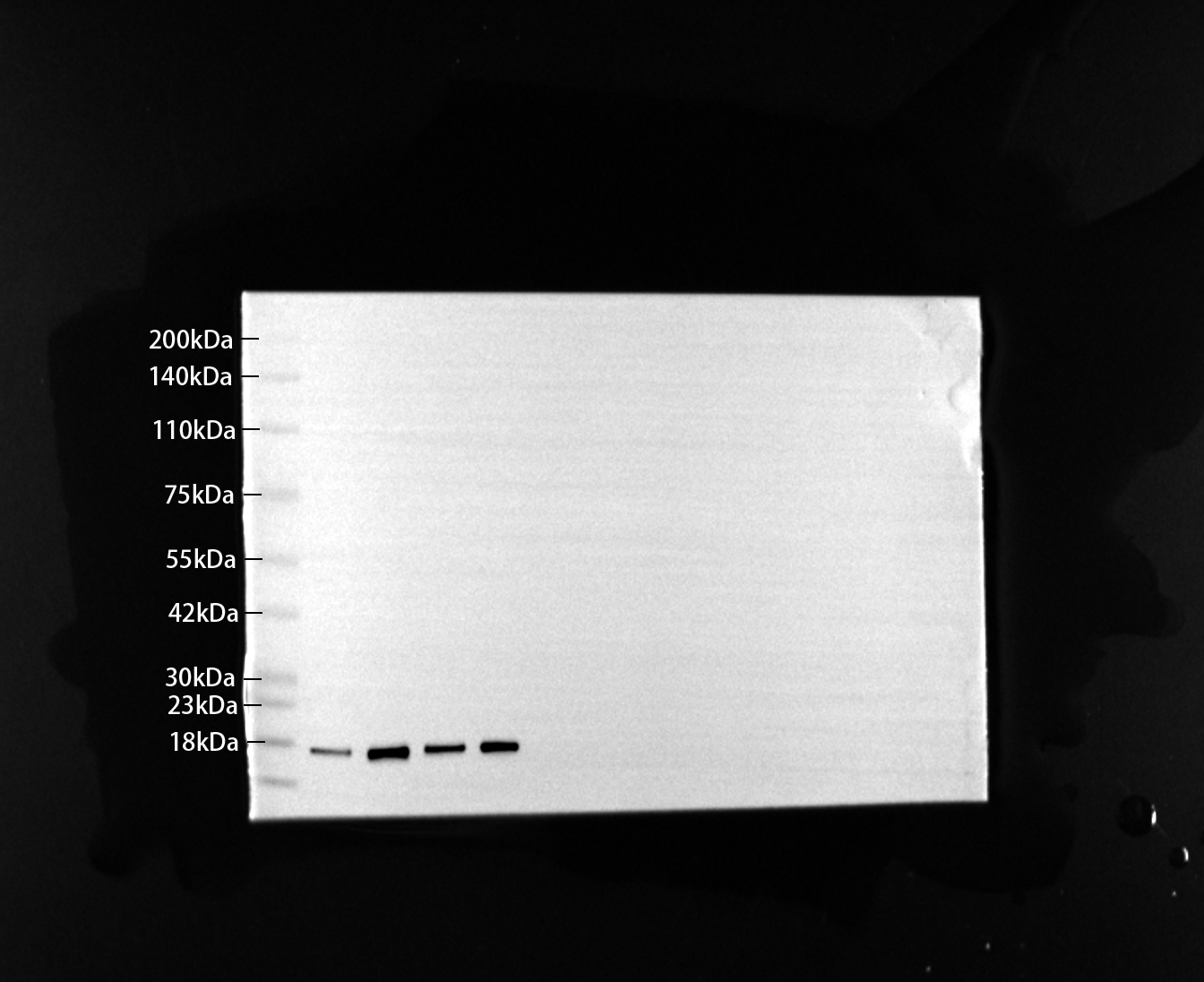

Supplement: Supplementary file 1 — Supplementary Material 1. [file 41065_2026_674_MOESM1_ESM.zip › Original image for western blot -marker/Original image Figure 2H/2H-Cleaved caspase-3.tif]

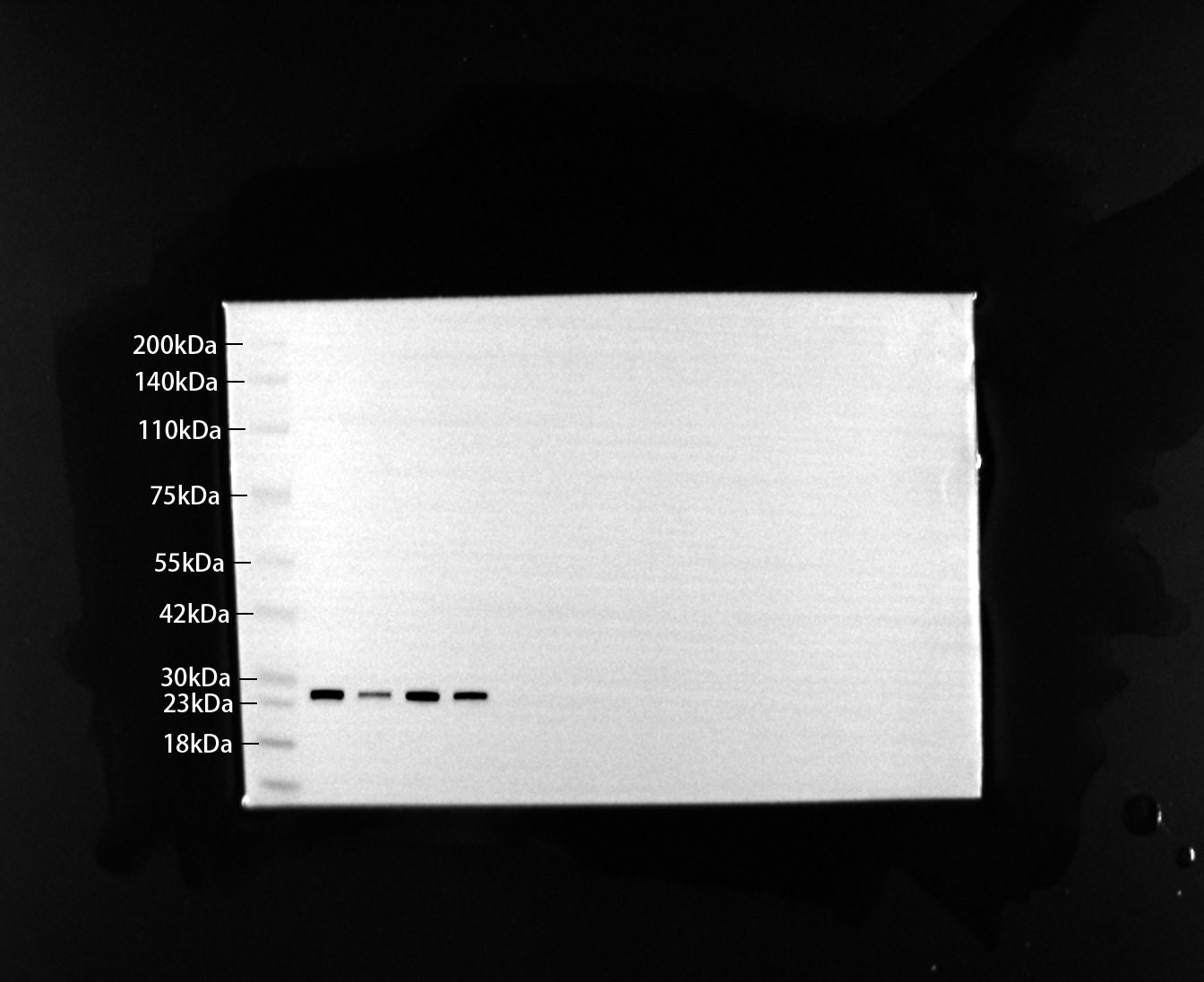

Supplement: Supplementary file 1 — Supplementary Material 1. [file 41065_2026_674_MOESM1_ESM.zip › Original image for western blot -marker/Original image Figure 2H/2H-BcI2.tif]

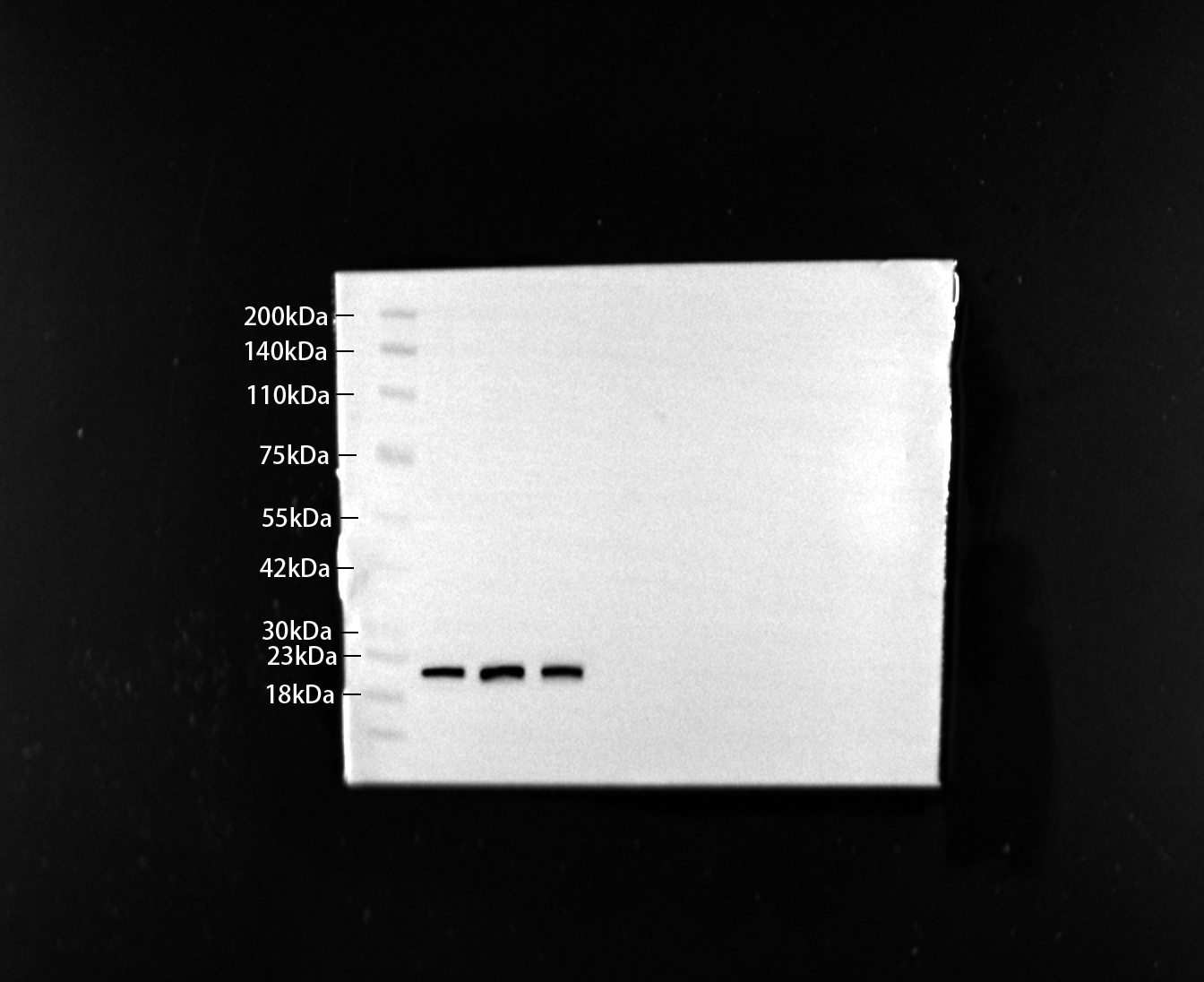

Supplement: Supplementary file 1 — Supplementary Material 1. [file 41065_2026_674_MOESM1_ESM.zip › Original image for western blot -marker/Original image Figure 1C/1C-Bax.tif]

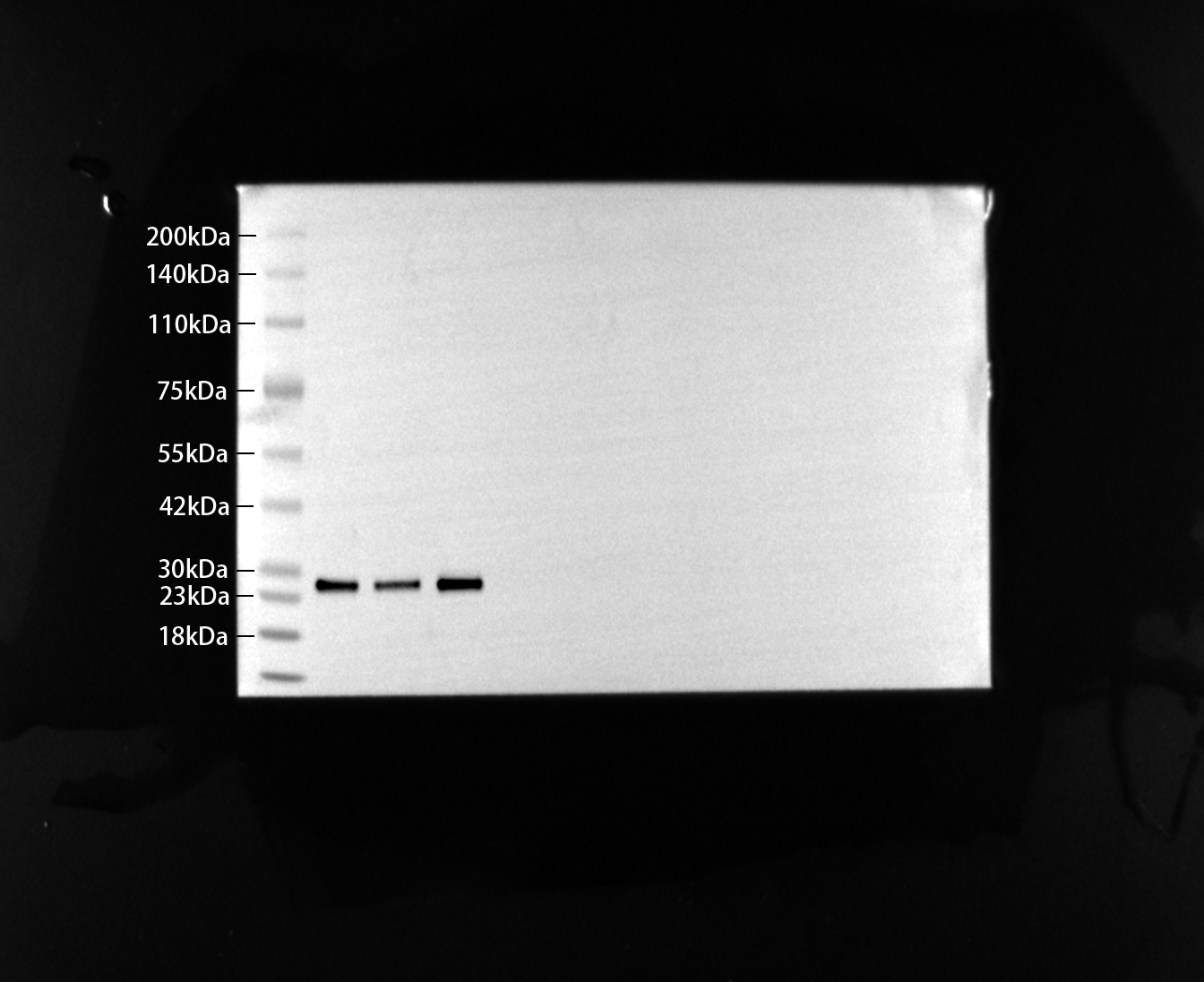

Supplement: Supplementary file 1 — Supplementary Material 1. [file 41065_2026_674_MOESM1_ESM.zip › Original image for western blot -marker/Original image Figure 1C/1C-BcI2.tif]

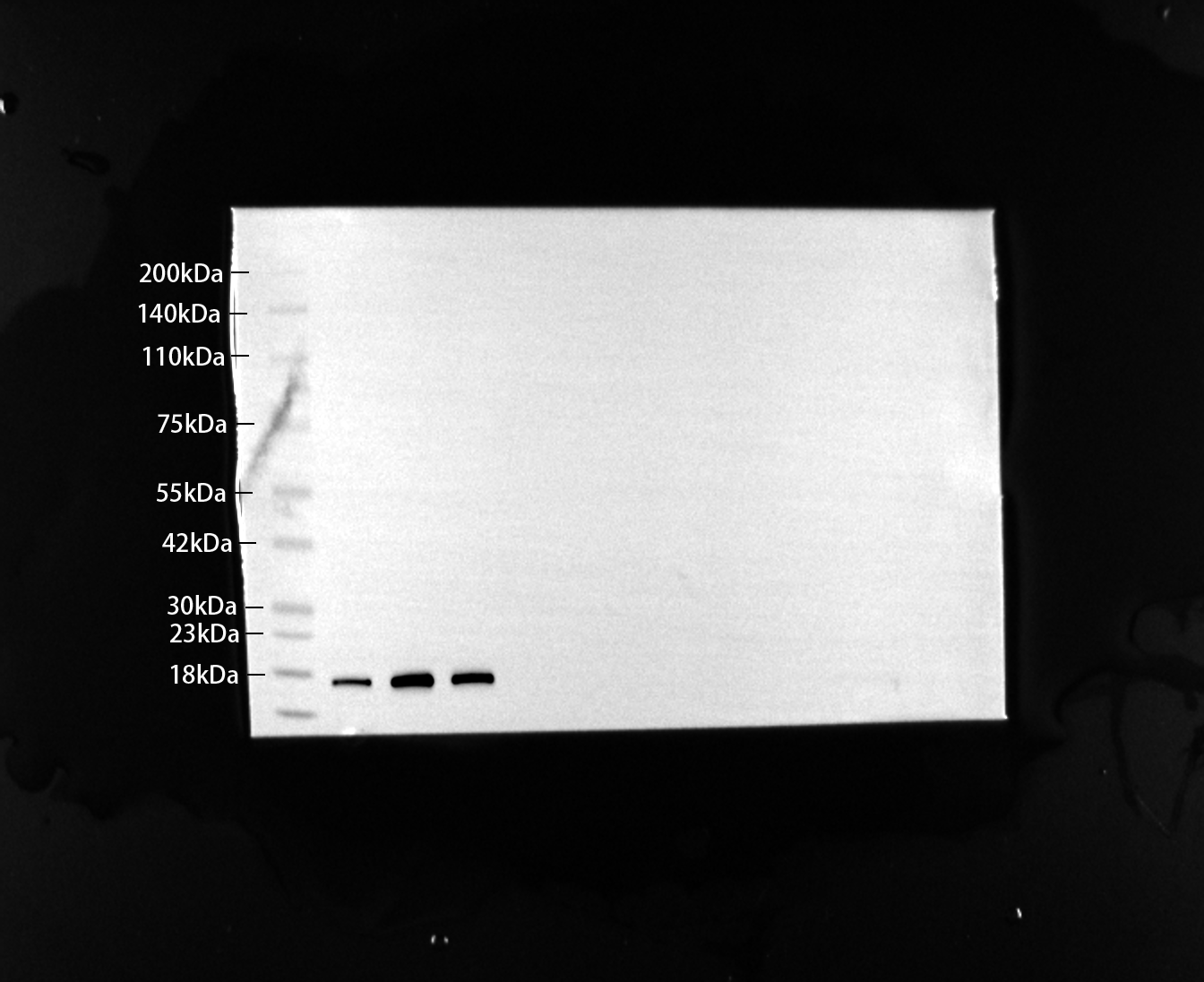

Supplement: Supplementary file 1 — Supplementary Material 1. [file 41065_2026_674_MOESM1_ESM.zip › Original image for western blot -marker/Original image Figure 1C/1C-Cleaved caspase-3.tif]

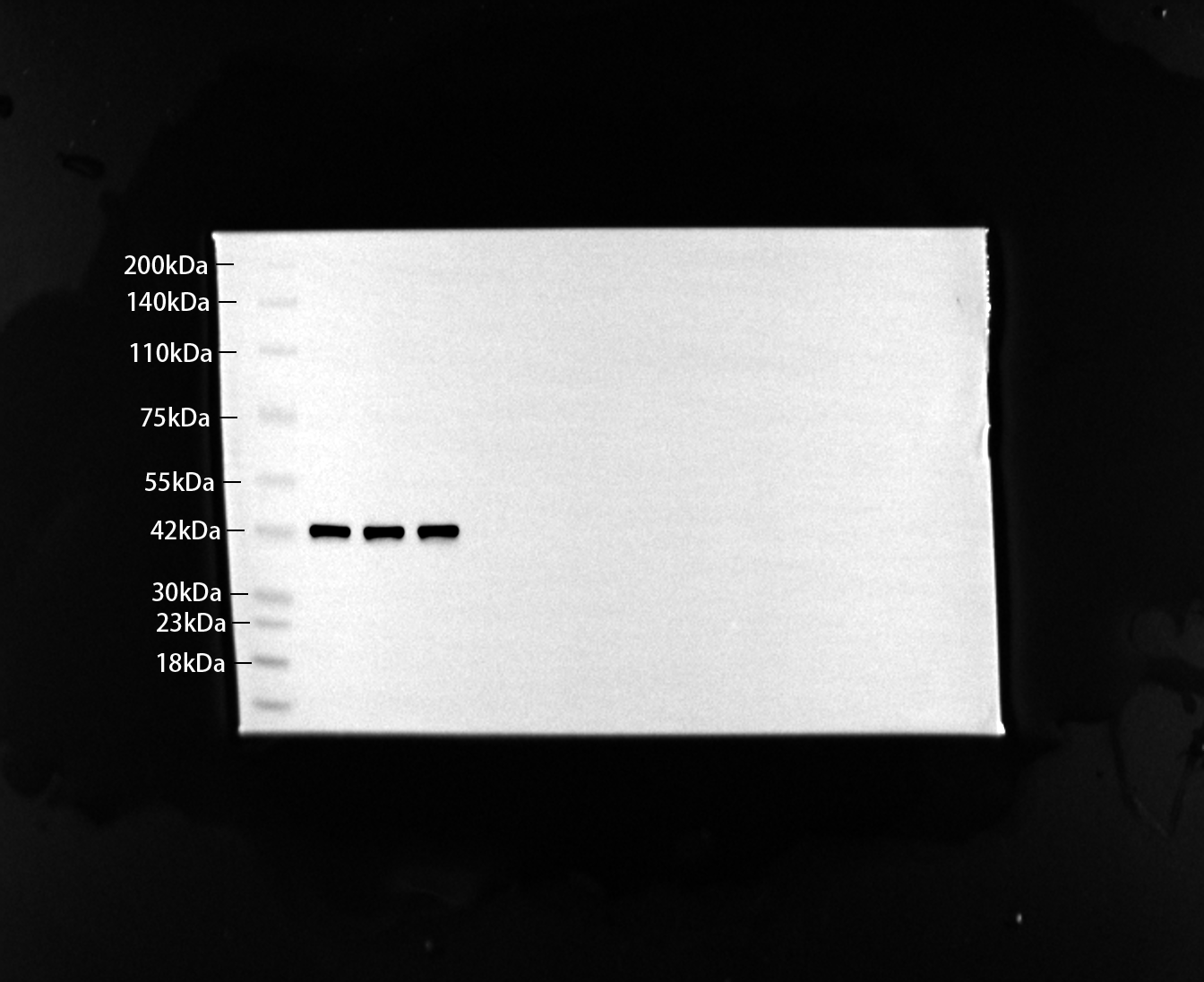

Supplement: Supplementary file 1 — Supplementary Material 1. [file 41065_2026_674_MOESM1_ESM.zip › Original image for western blot -marker/Original image Figure 1C/1C-β-actin.tif]

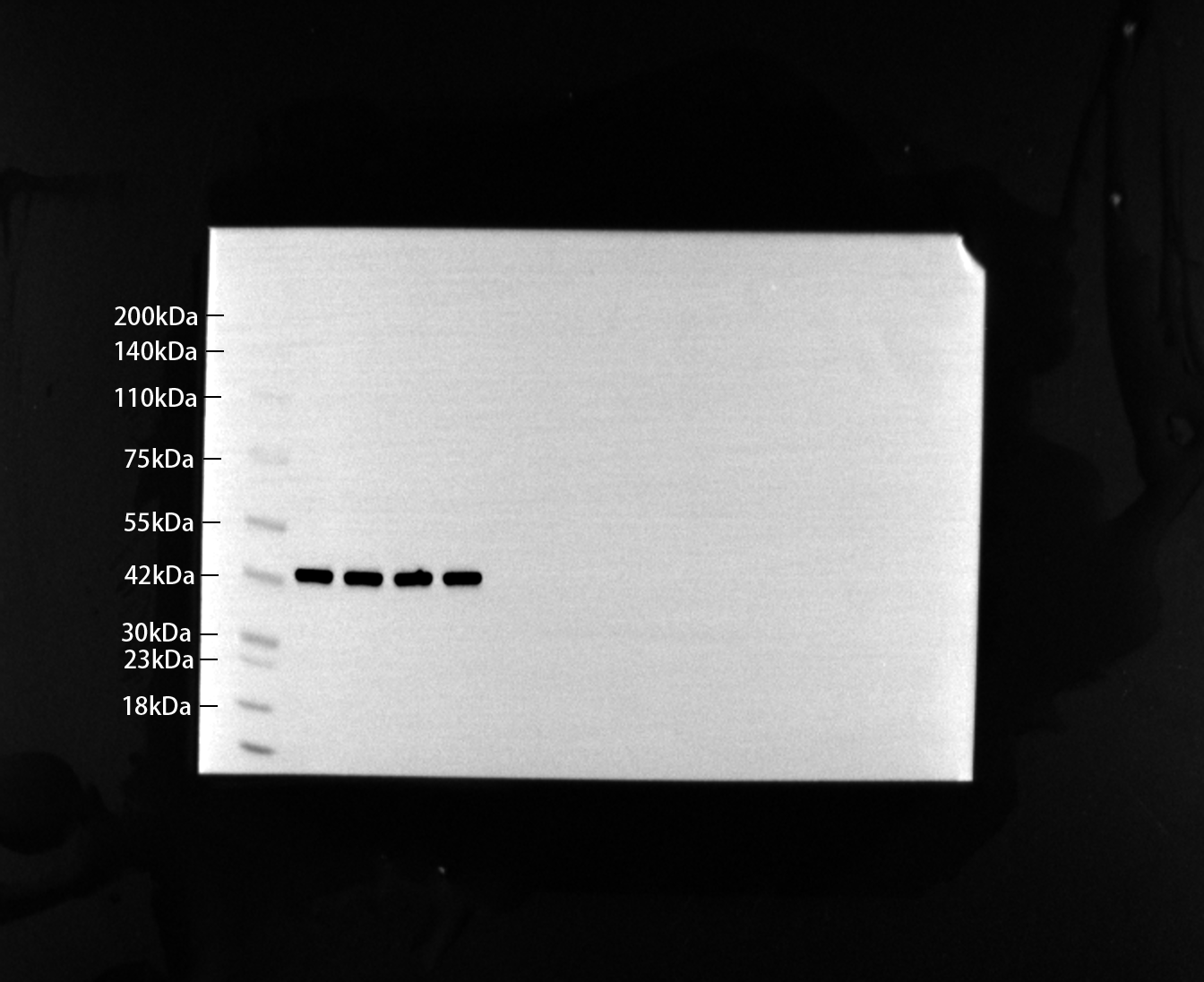

Supplement: Supplementary file 1 — Supplementary Material 1. [file 41065_2026_674_MOESM1_ESM.zip › Original image for western blot -marker/Original image Figure 6F/6F-β-actin.tif]

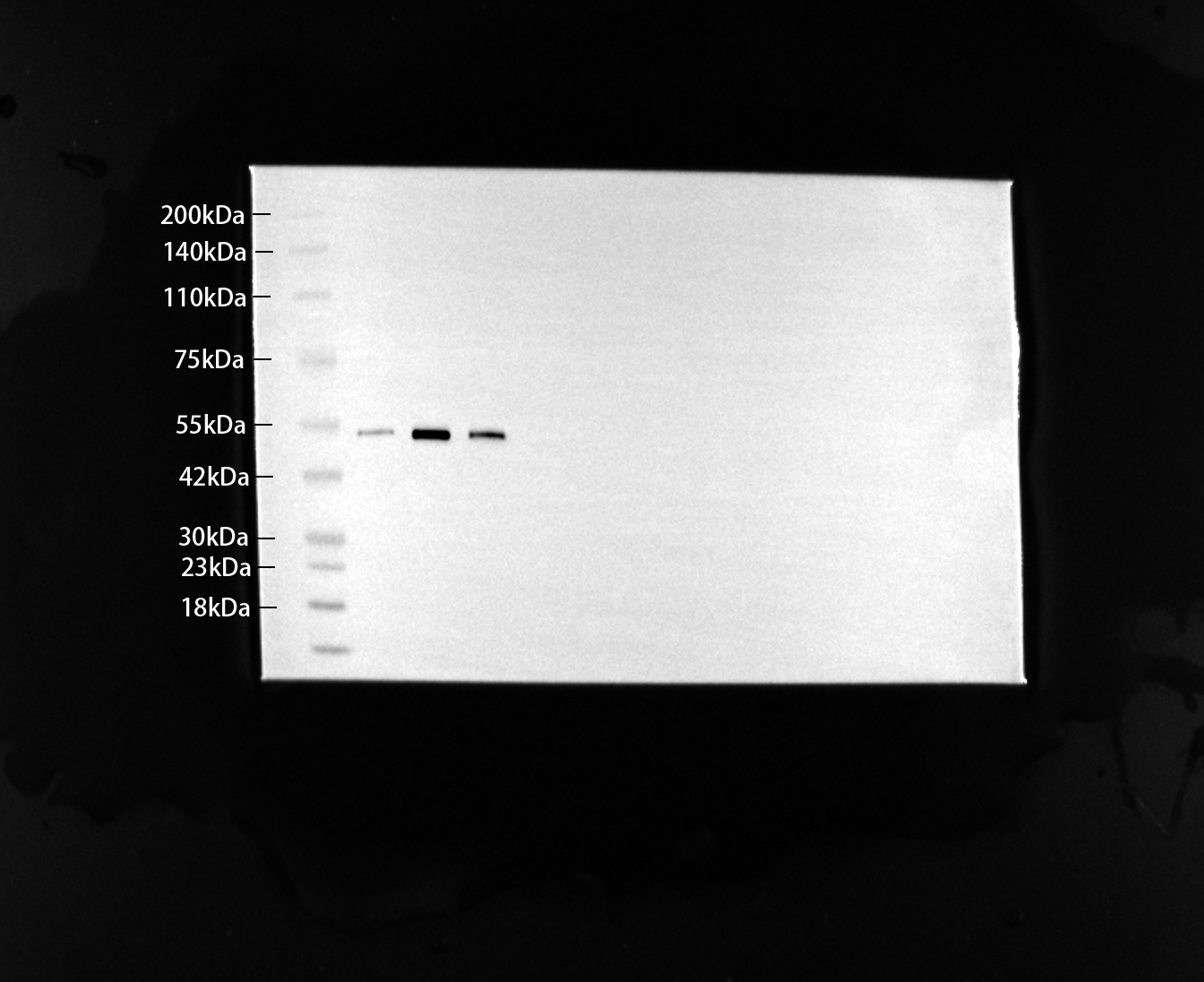

Supplement: Supplementary file 1 — Supplementary Material 1. [file 41065_2026_674_MOESM1_ESM.zip › Original image for western blot -marker/Original image Figure 1F/1F-MMP13.tif]

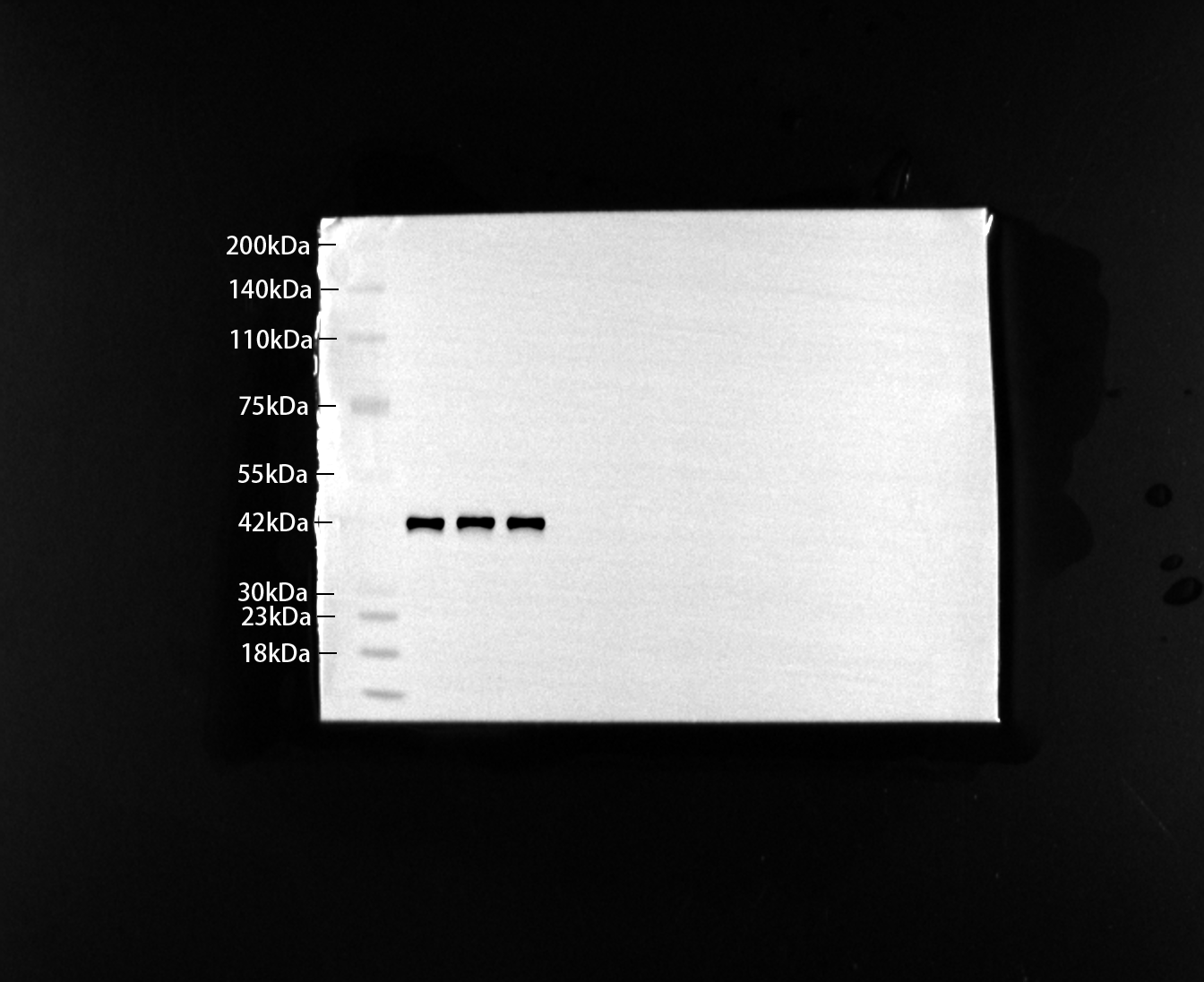

Supplement: Supplementary file 1 — Supplementary Material 1. [file 41065_2026_674_MOESM1_ESM.zip › Original image for western blot -marker/Original image Figure 1F/1F-β-actin.tif]

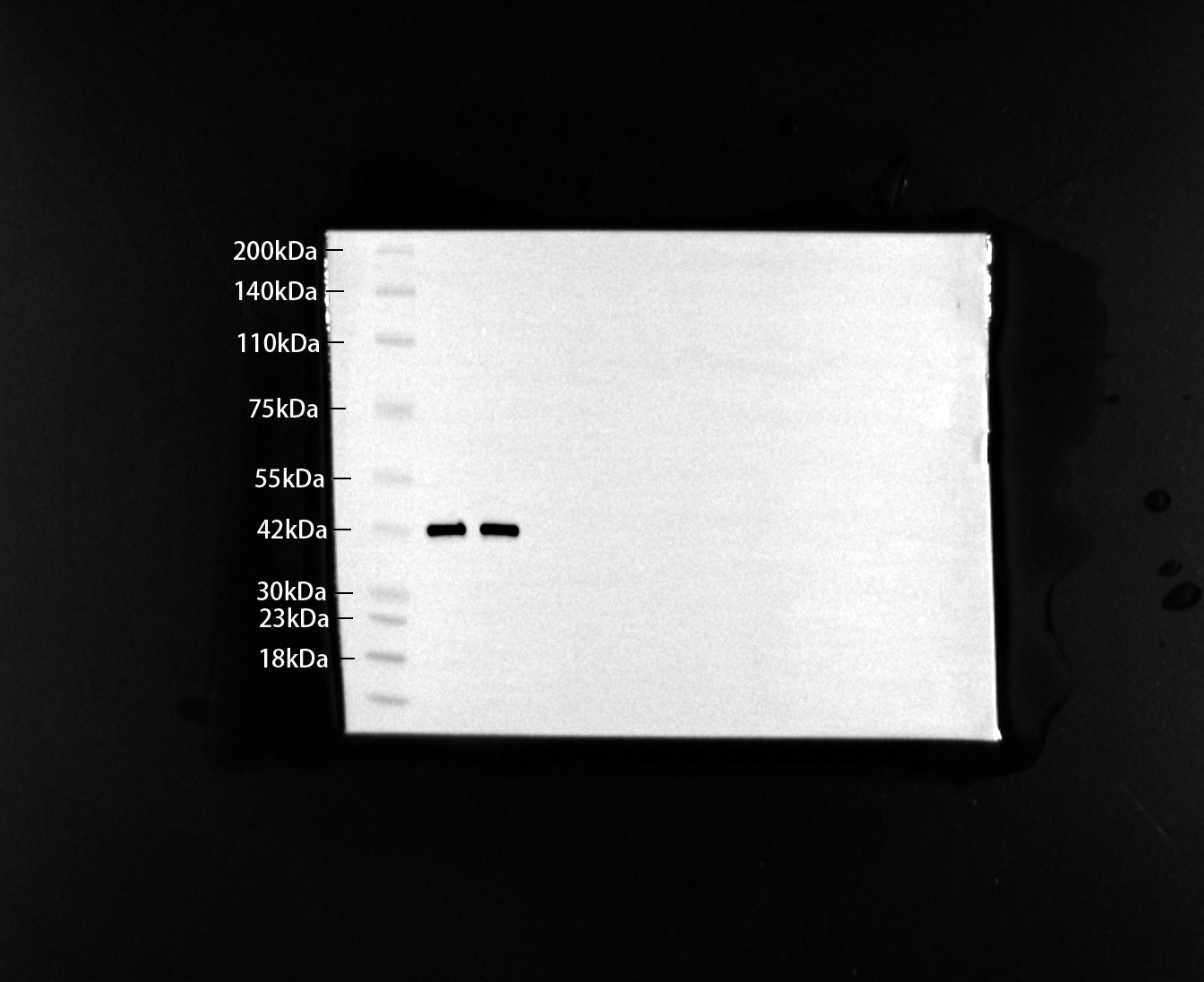

Supplement: Supplementary file 1 — Supplementary Material 1. [file 41065_2026_674_MOESM1_ESM.zip › Original image for western blot -marker/Original image Figure 2B/2B-β-actin.tif]

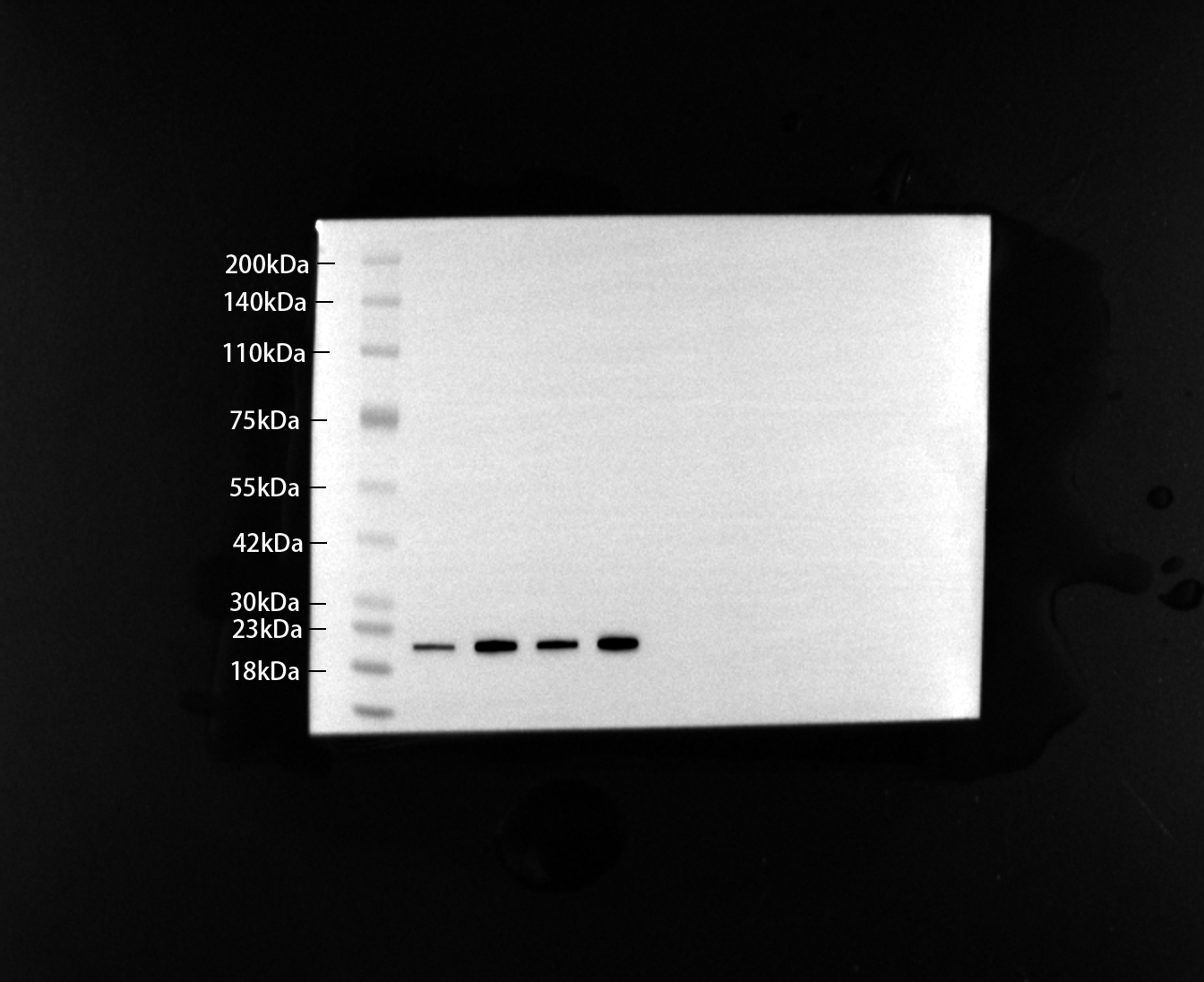

Supplement: Supplementary file 1 — Supplementary Material 1. [file 41065_2026_674_MOESM1_ESM.zip › Original image for western blot -marker/Original image Figure 2H/2H-Bax.tif]
